# Deubiquitinase USP24 activated by IL-6/STAT3 enhances PD-1 protein stability and suppresses T cell antitumor response

**DOI:** 10.1126/sciadv.adt4258

**Published:** 2025-04-16

**Authors:** Hung-Chia Hsieh, Ming-Jer Young, Kuan-Yu Chen, Wu-Chou Su, Chien-Chung Lin, Yi-Ting Yen, Jan-Jong Hung, Yi-Ching Wang

**Affiliations:** ^1^ Institute of Basic Medical Sciences, College of Medicine, National Cheng Kung University, Tainan 70101, Taiwan.; ^2^ Department of Biotechnology and Bioindustry Sciences, College of Bioscience and Biotechnology, National Cheng Kung University, Tainan 70101, Taiwan.; ^3^ Department of Pharmacology, College of Medicine, National Cheng Kung University, Tainan 70101, Taiwan.; ^4^ Department of Internal Medicine, National Cheng Kung University Hospital, College of Medicine, National Cheng Kung University, Tainan 70101, Taiwan.; ^5^ Department of Surgery, National Cheng Kung University Hospital, College of Medical College, National Cheng Kung University, Tainan 70101, Taiwan.

## Abstract

Persisting programmed cell death-1 (PD-1) signaling impairs T cell effector function, which is highly associated with T cell exhaustion and immunotherapy failure. However, the mechanism responsible for PD-1 deubiquitination and T cell dysfunction remains unclear. Here, we show that ubiquitin-specific peptidase 24 (USP24) promotes PD-1 protein stability by removing K48-linked polyubiquitin. Increased interleukin-6 level transcriptionally activates the USP24 expression, which leads to PD-1 stabilization. Furthermore, USP24 deficiency reduces PD-1 levels in CD8^+^ T cells and attenuates *Egfr^L858R^*-driven lung tumorigenesis in *Usp24^C1695A^* catalytic deficient mice. Targeting PD-1 stability with the USP24-specific inhibitor USP24-i-101 boosts cytotoxic T cell activity, restrains lung tumor growth, and achieves superior therapeutic effects when combined with anti-CTLA4 immunotherapy. Clinically, patients with lung cancer exhibiting high USP24 expression in tumor-infiltrating CD8^+^ T cells display exhausted features and show unfavorable responses to immunotherapy. Our findings dissect the mechanism for regulating enhanced PD-1 stability in tumor-infiltrating CD8^+^ T cells and reveal USP24 as a potential target of antitumor immunotherapy.

## Teaser

Cytokine IL-6 drives STAT3/NF-κB signaling to increase USP24 expression, which promotes PD-1 deubiquitination and stabilization.

## INTRODUCTION

The clinical success of programmed cell death-1 (PD-1) blockade highlights the importance of exploring its regulatory machinery. The PD-1 expression is induced in activated T cells to control abnormal T cell activation upon engagement with its ligand programmed cell death-ligand 1 (PD-L1) or PD-L2 (*1*). However, persistent PD-1 expression in chronic inflammation and cancers dampens T cell effector functions and facilitates tumor immune escapes (*2*, *3*). Excessive PD-1 expression on tumor-infiltrating leukocytes (TILs) is often coexpressed with other inhibitory receptors such as T cell immunoglobulin and mucin domain–containing protein 3 (Tim-3), lymphocyte-activation gene 3 (Lag-3), and cytotoxic T lymphocyte–associated protein 4 (CTLA-4) and drives T cell exhaustion (Tex), especially terminal exhaustion (*4*, *5*). The hyporesponsiveness of exhausted T cells to immune checkpoint agents is a major obstacle to cancer immunotherapy (*6*). Therefore, down-regulation of PD-1 levels on TILs could be a potential approach for restoring antitumor function and even reversing exhausted T cells.

PD-1 expression is tightly regulated at many levels from epigenetic regulation, transcriptional regulation, and posttranscriptional modifications to posttranslational modifications (PTMs) (*7*, *8*). Several studies have revealed the critical role of PTMs, including glycosylation, phosphorylation, palmitoylation, and ubiquitination, in regulating PD-1 expression and the efficacy of immune checkpoint blockade (ICB). For instance, ubiquitination plays a dominant role in the modulation of PD-1 protein stability, activity, quality control, and cellular localization (*9*). F-box only protein 38 (FBXO38) ubiquitinates PD-1 after internalization and undergoes a proteasome degradation system (*10*). Kelch-like family member 22 (KLHL22) induces degradation of incompletely glycosylated PD-1 before being transported to the plasma membrane (*11*). Casitas B lineage lymphoma (c-Cbl) reinforces T cell’s antitumor immunity by destabilizing PD-1 at the plasma membrane, leading to its ubiquitination and subsequent proteasomal degradation (*12*). Recently, FBW7 has been revealed to regulate PD-1 protein stability in the nucleus (*13*). Notably, ubiquitin-mediated degradation could be reversed by deubiquitinating enzymes (DUBs) (*14*). Thus far, the only DUB reported for PD-1 is ubiquitin-specific peptidase 5 (USP5) (*15*). Therefore, identifying additional DUBs, along with their specific inhibitors, to regulate PD-1 deubiquitination and T cell functions is of great importance for developing further therapeutic strategies.

USP24 functions as a DUB that recognizes and removes ubiquitin from the target protein (*16*). USP24 is overexpressed in various cancers. Targeting USP24 as a potential therapeutic strategy is an emerging area of exploration (*17*). Our previous studies found that USP24 shapes the tumor microenvironment (TME) by promoting the secretion of interleukin-6 (IL-6) in tumor-associated macrophages (TAMs) and cancer cells (*18*, *19*). In addition, we developed a specific USP24 inhibitor to overcome drug resistance acquired during chemotherapy or targeted therapy in lung cancer (*20*). However, the role of USP24 in regulating T cell activity in the TME to enhance the clinical efficacy of ICB remains elusive.

In this study, we reveal USP24 as a novel PD-1 regulator. USP24 promotes PD-1 stability by directly deubiquitinating and preventing it from proteasome-mediated degradation. IL-6 in the TME drives nuclear factor κB (NF-κB)/signal transducers and activators of transcription 3 (STAT3) signaling to transcriptionally increase USP24. Enhanced PD-1 level stabilized by USP24 augments PD-L1/PD-1 signaling, ultimately leading to T cell dysfunction and poor benefit of ICB. The USP24-specific inhibitor, USP24-i-101 alone or in combination with immunotherapy anti-CTLA4, profoundly reactivates T cell functions in the TME and markedly induces tumor inhibition.

## RESULTS

### Usp24^C1695A^ catalytic mutation mice establish superior T cell antitumor activity

**Fig. 1.**
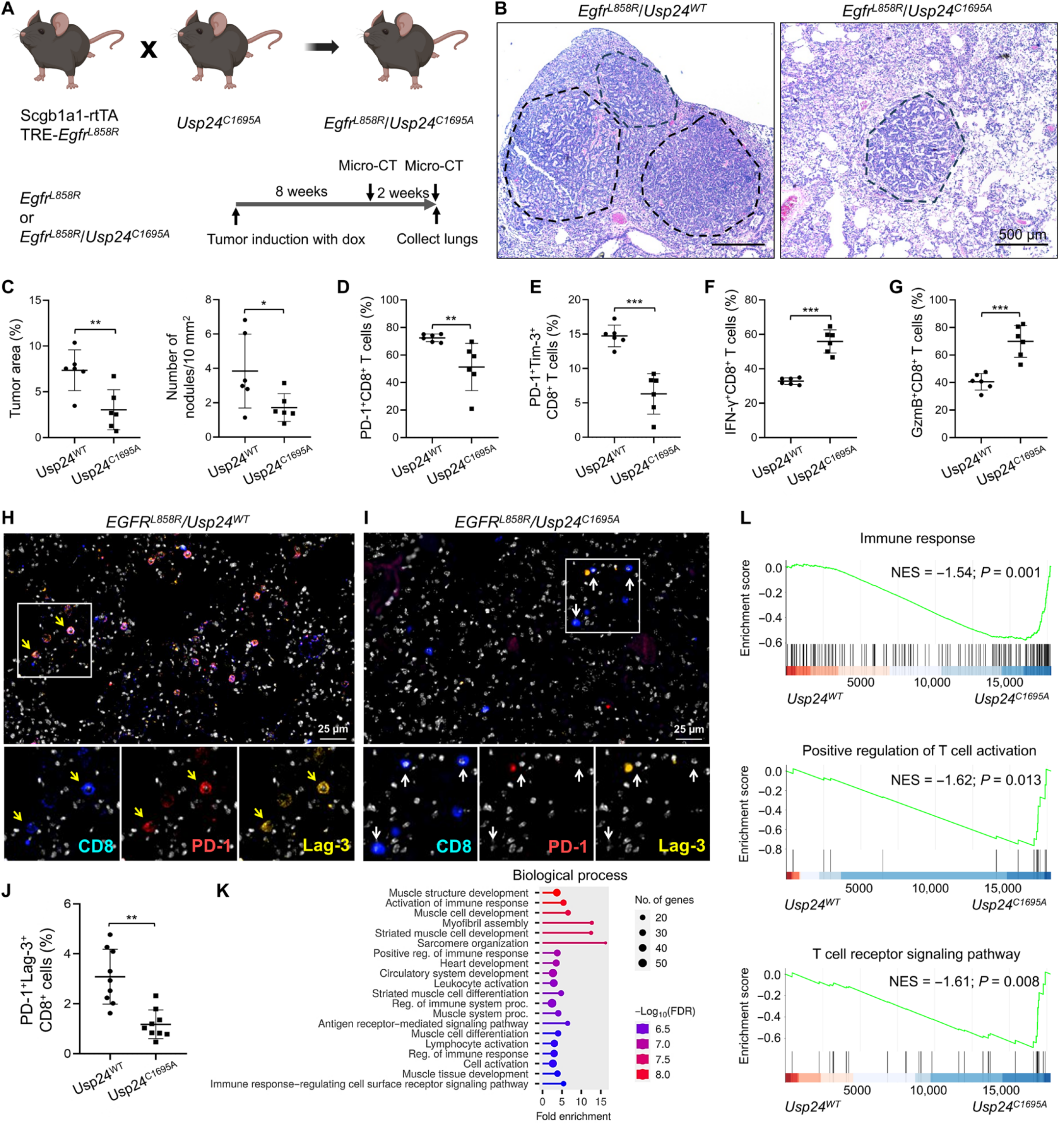
USP24 inactivation establishes an antitumor microenvironment. (**A**) Diagrams illustrating the strategy to generate *Egfr^L858R^/Usp24^C1695A^* mice and timeline of doxycycline (dox) induction. (**B**) Histopathology of the lung tissues at the end point of mouse experiments and representative H&E staining of lung tissue images are shown. (**C**) Quantification of lung tumor area and tumor nodule numbers. (**D** to **G**) Percentages of tumor-infiltrating PD-1^+^ (D), PD-1^+^Tim-3^+^ (E), IFN-γ^+^ (F), and GzmB^+^ (G) CD8^+^ T cells in *Egfr^L858R^/Usp24^WT^* or *Egfr^L858R^/Usp24^C1695A^* mice by flow cytometry. h, hours. (**H** and **I**) Multiplex IF-IHC staining of PD-1^+^Lag-3^+^CD8^+^ T cells (yellow arrows) in lung tumors from *Egfr^L858R^/Usp24^WT^* (H) or *Egfr^L858R^/Usp24^C1695A^* mice (I). Activated CD8^+^ T cells are indicated with white arrows. (**J**) Quantitative results of PD-1^+^Tim-3^+^CD8^+^ tumor-infiltrating CD8^+^ T cells of (H) and (I). Data are the means ± SEM. *P < 0.05; **P < 0.01; ***P < 0.001 (Student’s *t* test). (**K** and **L**) Total RNA isolated from the lung tumor of *Egfr^L858R^/Usp24^WT^* or *Egfr^L858R^/Usp24^C1695A^* mice was applied to RNA-seq to analyze the expression profile. (K) The top 20 enriched biological processes were analyzed by the ShinyGO 0.80 website. (L) GSEA showing up-regulation of immune response and T cell activation pathways in lung tumor of *Egfr^L858R^/Usp24^C1695A^* mice. FDR, false discovery rate; NES, normalized enrichment score.

We previously revealed that USP24 promotes lung tumor metastasis and enhances IL-6 secretion in TAMs to contribute to an immunosuppressive TME (*18*, *19*). To further investigate the immunomodulating effects of USP24, we generated triple transgenic mice (*Scgb1a1-rtTA*; TetO-*Egfr^L858R^*; *Usp24^C1695A/C1695A^*) in the C57BL/6J immune-competent mice, in which *Egfr^L858R^* mutation–driven lung tumor formation was developed in the condition of Usp24 with catalytic mutation (fig. S1A and Fig. 1A). After 10 weeks of doxycycline induction, mice expressing *Egfr^L858R^*/*Usp24^C1695A^* had reduced tumor nodules and areas compared to *Egfr^L858R^*/*Usp24^WT^* mice (Fig. 1, B and C, and fig. S1B). These results indicated that functional depletion of USP24 attenuates tumorigenesis in TetO-*Egfr^L858R^* mice, supporting its oncogenic role.

Next, we explored whether USP24 inactivation promotes antitumor immunity. We observed a significant decrease in the number of tumor-infiltrating dysfunctional CD8^+^ T cells defined by PD-1^+^ and PD-1^+^Tim-3^+^ populations. In contrast, the activity of CD8^+^ T cells evidenced by interferon-γ^+^ (IFN-γ^+^) or granzyme B^+^ (GzmB^+^) was apparently increased in *Usp24^C1695A^* mice compared to *Usp24^WT^* mice (Fig. 1, D to G). Notably, a reduced population of regulatory T cells (T_regs_) and PD-1^+^ TAMs was also observed in tumors from *Usp24^C1695A^* mice (fig. S1, C to E). Immunofluorescence (IF) staining demonstrated highly expressed Usp24 in tumor-infiltrating CD8^+^ T cells with PD-1^+^Tim-3^+^ exhaustion features from *Usp24^WT^* mice (Fig. 1, H and J), while it was less frequent in lung tumors from *Usp24^C1695A^* mice (Fig. 1, I and J). Mice with USP24 inactivation had a decreased population of dysfunctional T cells with increased activity of both CD8^+^ and CD4^+^ T cells from draining lymph nodes (fig. S1, F and G). There were no enlargement and aberrant pro-inflammatory cytokine deposition in the spleen of *Usp24^C1695A^* mice (fig. S1, H and I), suggesting that Usp24 inactivation did not induce an autoimmune response. These findings indicate that *Usp24^C1695A^* establishes a superior antitumor immunity and attenuates lung tumor formation.

To elucidate the underlying mechanism of increased antitumor activity owing to USP24 inactivation, we collected lung tumor specimens from *Usp24^WT^* and *Usp24^C1695A^* mice and performed RNA sequencing (RNA-seq) analysis (GSE281983). The Gene Ontology analysis demonstrated that genes related to activation of immune response and lymphocyte activation signal pathways were enriched when USP24 was inactivated (Fig. 1K and fig. S1J). Gene set enrichment analysis (GSEA) verified that the USP24 catalytic mutant up-regulated genes involved in immune response, T cell activation, and differentiation signaling pathways (Fig. 1L and fig. S1K). Together, USP24 enzyme inactivation promotes T cell activity to restrain cancer progression.

### USP24 promotes PD-1 protein stability, leading to T cell inactivation

Aberrant PD-1 expression on T cells correlates with T cell dysfunction and ICB failure (*21*). We found that USP24 showed a stronger positive correlation with Tex gene signatures including PD-1, CTLA-4, Lag-3, TIGIT, and BTLA in patients with lung adenocarcinoma (*n* = 515) by the database from the TIMER 2.0 websites (fig. S2A). Moreover, USP24 expression along with Tex gene signatures was up-regulated in tumor-infiltrating CD8^+^ T cells from ICB nonresponder (NR) patients (GSE111414) (fig. S2B), suggesting that USP24 is highly associated with T cell dysfunction. The knockdown screen of USP24 and other USPs showed that shUSP24 decreased PD-1 levels in Jurkat T cells, with shUSP47 and shUSPL1 showing a lesser effect (fig. S2C). Because the *Usp24*^C1695A^ catalytic mutant mice displayed a reduced PD-1 abundance in CD8^+^ and CD4^+^ T cells, we hypothesized that USP24 controlled PD-1 protein stability in T cells.

**Fig. 2.**
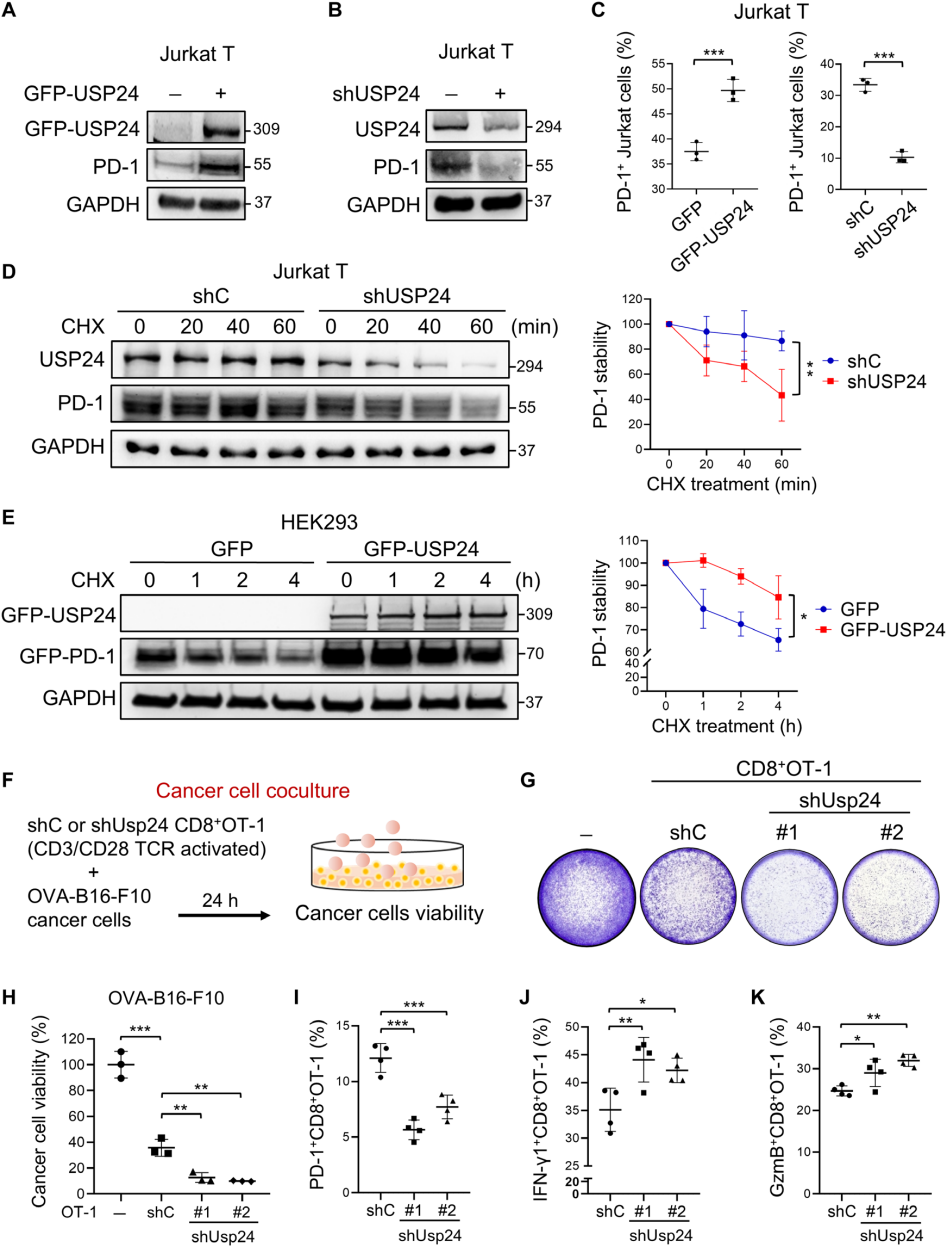
USP24 increases PD-1 protein stability and leads to T cell inactivation. (**A** and **B**) Jurkat T cells were transfected with GFP-USP24 plasmid (A) or transduced with shUSP24 (B) to analyze PD-1 expression by Western blotting. (**C**) Jurkat T cells with overexpression or knockdown of USP24 were stimulated with PMA (100 ng/ml) and ionomycin (1 μg/ml) for 6 hours and subjected to flow cytometry for PD-1 detection. (**D** and **E**) shC and shUSP24 Jurkat T cells stimulated with PMA/ionomycin for 6 hours (D) or HEK293 cells ectopically expressing GFP-USP24 and GFP-PD-1 (E) were treated with the protein synthesis inhibitor CHX (50 μg/ml) for the indicated times. GAPDH (glyceraldehyde-3-phosphate dehydrogenase) was used as an internal control. (**F** to **H**) In vitro T cell–mediated killing assays. (F) CD8^+^OT-1^+^ T cells were introduced with shC or shUsp24 lentiviruses and then cocultured with OVA-B16-F10 cancer cells (effector cell–to–target cell ratio, 5:1) for 24 hours. (G) Crystal violet staining for cancer cell viability measurement. (H) Quantification using ImageJ software. (**I** to **K**) Detection of PD-1^+^, IFN-γ^+^, and GzmB^+^CD8^+^OT-1^+^ T cells by flow cytometry after coculturing with OVA-B16-F10 cancer cells. Data are the means ± SEM. *P < 0.05; **P < 0.01; ***P < 0.001. Student’s *t* test [(D) and (E)] and one-way ANOVA [(H) to (K)].

First, we confirmed whether USP24 regulated PD-1 expression. Jurkat T and human embryonic kidney (HEK) 293 cells ectopically expressing wild-type USP24 (USP24-WT), but not the catalytic dead mutant construct at C1698A (USP24-C1698A, CA), notably up-regulated PD-1 protein expression (Fig. 2A and fig. S2, D and E). Notably, knockdown of USP24 (shUSP24) decreased PD-1 levels in Jurkat T cells (Fig. 2B). Moreover, ectopically expressing GFP-USP24 increased PD-1 membrane presentation, whereas knockdown of USP24 reduced the PD-1 level on the cell surface (Fig. 2C). USP24 deficiency decreased PD-1 protein expression without altering the *PDCD1* mRNA level in peripheral blood mononuclear cells (PBMCs) and mouse CD8^+^ splenic T cells (fig. S2, F to H), implicating that USP24 regulates PD-1 protein stability. Cycloheximide (CHX) chase assay demonstrated a decrease in PD-1 protein stability in USP24-knockdown Jurkat T cells (Fig. 2D). Conversely, overexpression of USP24-WT prolonged PD-1 protein half-life in HEK293 cells, whereas this effect was not observed in cells transduced with USP24-CA (Fig. 2E and fig. S2, I and J). These results suggested the positive regulation of PD-1 protein stability by USP24.

Notably, shUSP24 reduced PD-1 expression in Jurkat T cells, leading to decreased recombinant PD-L1 protein staining (fig. S2K). These results prompted us to examine whether USP24 inhibition blocks PD-1/PD-L1–mediated T cell inhibition. For in vitro T cell killing assays, activated OT-1 CD8^+^ T cells were infected with lentiviruses expressing shC or shUsp24 and cocultured with ovalbumin (OVA)–expressing B16-F10 (OVA-B16-F10) cancer cells (Fig. 2F). Usp24-deficient OT-1 CD8^+^ T cells induced higher cytotoxicity compared to the control group (Fig. 2, G and H). In addition, Usp24 depletion decreased PD-1 surface levels (Fig. 2I), resulting in increased OT-1 CD8^+^ T antitumor activity (Fig. 2, J and K). We further clarify whether enhanced T cell cytotoxicity observed with shUsp24 is directly attributed to the down-regulation of PD-1 levels on OT-1 T cells. We observed that the tumor-killing effects of Usp24-deficient OT-1 T cells were comparable to anti-PD-1 antibody (α-PD-1) treatment alone. Moreover, α-PD-1 treatment did not further improve OT-1 T cell tumor-killing effects mediated by shUSP24, suggesting that enhanced T cell cytotoxicity mediated by USP24 inhibition is directly attributed to the decreased PD-1 levels (fig. S2L). In line with the mouse T cell coculture system, activated human PBMCs with USP24 deficiency also induced stronger killing effects when cocultured with H1299 cancer cells (fig. S2M). Moreover, the activated IFN-γ^+^ and GzmB^+^CD8^+^ T cell populations were up-regulated in shUSP24-PBMCs after coculturing with cancer cells (fig. S2, N and O). Together, our findings suggested that USP24 enhances PD-1 stability and therefore leads to T cell immunosuppression.

### USP24 stabilizes PD-1 by deubiquitinating it in T cells

**Fig. 3.**
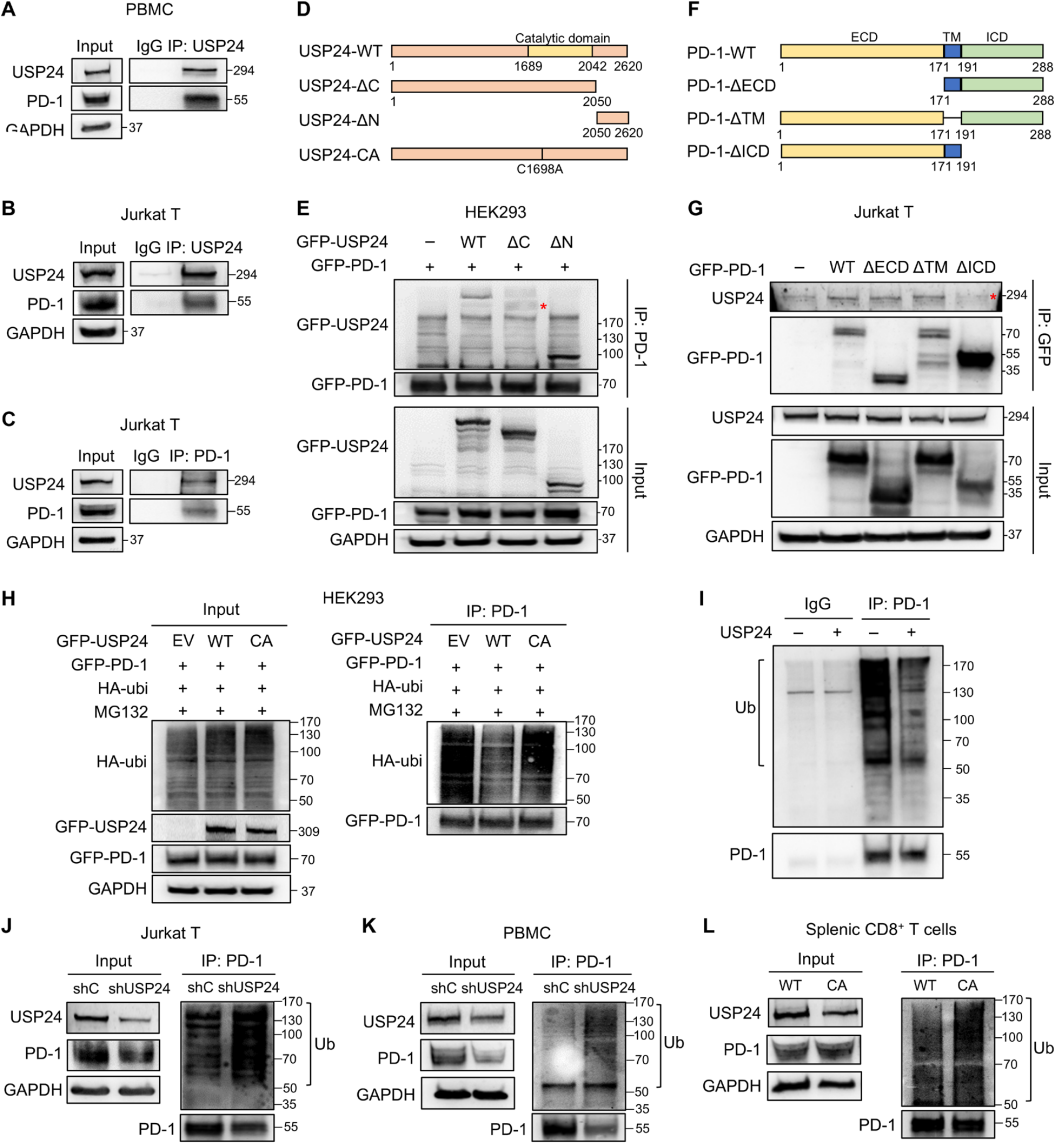
USP24 interacts with PD-1 for deubiquitination. (**A** to **C**) IP of endogenous USP24 in PBMCs stimulated with anti-CD3/CD28 (A) and Jurkat T cells stimulated with PMA/ionomycin for 6 hours (B) and endogenous PD-1 in Jurkat T cells (C). (**D** to **G**) Schematic diagram and domain interaction analysis of USP24 and PD-1. The maps illustrated the protein sequence of indicated USP24 (D) and PD-1 (F) domain deletion constructs. (E) IP of GFP-PD-1 in HEK293 cells transduced with WT-PD-1 along with the indicated USP24 constructs. (G) IP of GFP-PD-1 in Jurkat T cells transfected with the indicated PD-1 constructs. (**H**) HEK293 cells were transfected with PD-1 and hemagglutinin-ubiquitin (HA-ubi), together with EV, USP24-WT, or USP24-CA plasmid. Cell lysates were subjected to IP assay using anti-PD-1 antibody. (**I**) In vitro deubiquitination assays were conducted. Ubiquitinated PD-1 was immunoprecipitated in PMA/ionomycin–stimulated Jurkat T cells using an anti-PD-1 antibody followed by incubating with or without USP24 recombinant protein for 2 hours. Ub, ubiquitin. (**J** to **L**) shC and shUSP24 Jurkat T cells (J), PBMCs (K), or splenic CD8^+^ T cells from *Egfr^L858R^/Usp24^WT^* or *Egfr^L858R^/Usp24^C1695A^* mice (L) were stimulated with anti-CD3/CD28 for 24 hours and treated with MG132 for 6 hours before harvest. Lysates were immunoprecipitated with anti-PD-1 antibodies to detect PD-1 ubiquitination levels.

USP24 is a deubiquitinase that removes ubiquitin from its substrates and protects them from degradation (*18*). We hypothesized that USP24 directly interacts with PD-1 to cleave ubiquitin on PD-1. Immunoprecipitation (IP) and reverse IP-Western assays showed the interaction between endogenous USP24 and PD-1 proteins in PBMCs and Jurkat T cells (Fig. 3, A to C). The interaction was confirmed in Jurkat T cells and HEK293 cells ectopically transduced with GFP-USP24 (fig. S3, A and B). We previously revealed the substrate binding domain in the C-terminus of USP24 (*22*). To determine which domain on USP24 was essential for PD-1 interaction, we generated a USP24 C-terminal deletion construct (USP24-ΔC, amino acids 1 to 2050) and an N-terminal deletion construct (USP24-ΔN, amino acids 2050 to 2620) (Fig. 3D). USP24-ΔC abolished its binding to PD-1 in HEK293 cells (Fig. 3E), suggesting the critical region of USP24 (amino acids 2050 to 2620) for interaction with PD-1. Given that the PD-1 protein contains an extracellular domain (ECD), a transmembrane domain (TM), and an intracellular domain (ICD) (*23*), we generated domain deletion constructs to investigate the essential domain for USP24 interaction (Fig. 3F). Deletion of ICD, but not the extracellular domain or transmembrane domain on PD-1, abolished USP24 interaction in Jurkat T cells (Fig. 3G). In addition, USP24 failed to reduce ubiquitin levels of PD-1 when its ICD was deleted (fig. S3C), indicating that USP24 binds to the intracellular region of PD-1 to facilitate deubiquitination.

Next, we validated that PD-1 is a substrate of USP24. Overexpression of USP24-WT, but not USP24-CA, reduced polyubiquitination on PD-1 (Fig. 3H). In vitro deubiquitination assay confirmed USP24 catalytic activity on PD-1 (Fig. 3I), supporting the notion that USP24 is a PD-1 deubiquitinase. In contrast, USP24 knockdown strongly elevated ubiquitinated levels of PD-1 in Jurkat T cells and PBMCs (Fig. 3, J and K). In addition, splenic CD8^+^ T cells from *Usp24^C1695A^* mice showed increased PD-1 ubiquitination (Fig. 3L). Because PD-1 protein could be modified by K48-linked polyubiquitin to undergo proteasome degradation (*10*), we speculated that USP24 cleaved K48-linked polyubiquitin on PD-1. IP-Western assay showed that USP24 predominantly removed K48-linked and partially K63-linked ubiquitin on PD-1 (fig. S3D). Moreover, the proteasome inhibitor MG132 but not the lysosomal inhibitor chloroquine treatment completely rescued PD-1 protein expression in USP24-knockdown Jurkat T cells (fig. S3E), indicating that USP24 protects PD-1 from K48 polyubiquitin linkage–mediated degradation. c-Cbl is a membrane-associated E3 ligase that ubiquitinates PD-1 for proteasome degradation (*12*). We further investigated whether USP24 competed with c-Cbl interacting with PD-1. USP24 overexpression reduced the binding between PD-1 and c-Cbl (fig. S3F), while knockdown of USP24 increased c-Cbl interacting with PD-1 (fig. S3G), suggesting that USP24 deubiquitinates and stabilizes PD-1 in part by counteracting c-Cbl–mediated ubiquitination.

### IL-6 increases USP24 expression to enhance PD-1 stability

**Fig. 4.**
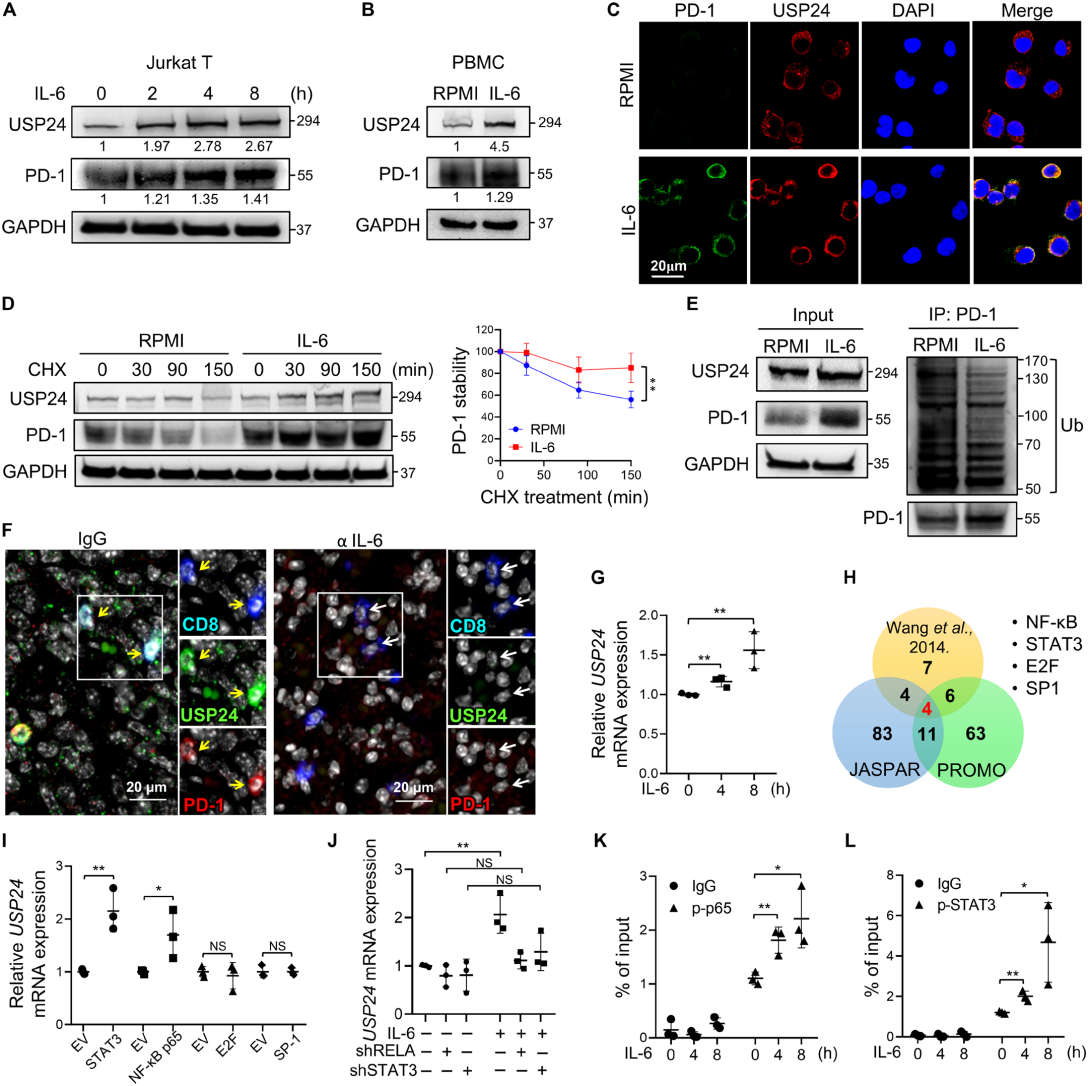
IL-6 up-regulates USP24 expression to enhance PD-1 stability in T cells. (**A** and **B**) Immunoblots of PD-1 and USP24 in Jurkat T cells with IL-6 (50 ng/ml) stimulation for the indicated times (A) and in PBMC for 4-hour stimulation (B). (**C**) IF staining of USP24 and PD-1 in Jurkat T cells with IL-6 for 4 hours. (**D**) Jurkat T cells were treated with IL-6 for 6 hours and subjected to CHX chase assay to detect the PD-1 protein half-life. (**E**) PD-1 ubiquitination levels by IP assay were measured in Jurkat T cells with MG132 treatment together with or without IL-6 stimulation for 6 hours. (**F**) IF-IHC staining of infiltrating exhausted USP24^+^PD-1^+^CD8^+^ T cells (yellow arrows) and active CD8^+^ T cells (white arrows) in tumor specimens from IgG- or α-IL-6–treated tumor-bearing mice. (**G**) RT-qPCR for detecting *USP24* mRNA expression in Jurkat T cells with IL-6 treatment at the indicated times. (**H**) Venn diagram showing the overlapped potential USP24-targeting transcriptional factor predicted from PROMO, JASPAR software, and the literature. (**I**) RT-qPCR for detecting *USP24* mRNA expression in Jurkat T cells transfected with indicated plasmids. NS, not significant. (**J**) *USP24* mRNA expression in shRELA or shSTAT3 Jurkat T cells with IL-6 treatment for 8 hours. (**K** and **L**) ChIP-qPCR assay was performed using p-p65 (K) or anti-p-STAT3 antibody (L) in Jurkat T cells treated with IL-6 at the indicated times. IgG was used as the negative control. Data are the means ± SEM (*n* = 3). *P < 0.05; **P < 0.01. Student’s *t* test [(D), (G), (I), (K), and (L)] and one-way ANOVA (J).

Persistent antigen and inflammatory cytokine signal exposures in the TME are often associated with T cell dysfunction (*24*). Multiple studies support the finding that IL-6 levels are elevated in the TME and correlate with T cell dysfunction (*25*, *26*). We previously demonstrated that TAM-derived IL-6 transcriptionally activates *PDCD1* expression via STAT3 signaling in T cells (*25*). We observed increased STAT3 activity followed by up-regulated *PD-1* mRNA levels within 2 hours after IL-6 stimulation in Jurkat T cells (fig. S4, A and B). Upon IL-6 stimulation, PD-1 levels and USP24 protein expression were continuously up-regulated and colocalized in Jurkat T cells, suggesting that IL-6 promoted PD-1 protein expression (Fig. 4, A to C, and fig. S4C). CHX assay showed that PD-1 protein became more stable upon IL-6 stimulus (Fig. 4D). IP-Western results further confirmed that IL-6 treatment increased interaction between USP24 and PD-1 (fig. S4D). In addition, PD-1 ubiquitinated levels were modestly reduced after IL-6 treatment (Fig. 4E). These findings suggested that IL-6 induced PD-1 abundance not only through transcriptional regulation but also via PTM modifications. To verify IL-6–promoted PD-1 stability via USP24, the knockdown of USP24 attenuated IL-6–induced PD-1 expression and cell surface level in Jurkat T cells (fig. S4, E and F). Notably, IL-6–promoted Tex features (PD-1^+^Tim-3^+^) were attenuated by USP24 inhibition (fig. S4G). Furthermore, treatment with an IL-6 neutralizing antibody (α-IL-6) reduced PD-1 abundance in tumor-infiltrating CD8^+^ T cells (Fig. 4F). Together, IL-6 transcriptionally up-regulates PD-1 expression and stability mediated by USP24 in T cells within the TME.

*USP24* mRNA expression was up-regulated upon IL-6 treatment (Fig. 4G). Next, we investigated which transcriptional factor controlled USP24 expression. By using PROMO and JASPAR prediction software, along with a literature search (*27*), the interaction of these three databases showed four potential transcriptional factors, NF-κB, STAT3, PAX-5, and SP1 on the *USP24* promoter (Fig. 4H and fig. S4H). Overexpression of NF-κB and STAT3, but not E2F and SP1, up-regulated *USP24* mRNA expression in Jurkat T cells (Fig. 4I). Knockdown of RELA or STAT3 attenuated IL-6–induced *USP24* mRNA expression (Fig. 4J and fig. S4, I and J). Chromatin IP-quantitative polymerase chain reaction (ChIP-qPCR) showed significantly increased binding of p-NF-κB and p-STAT3 to the *USP24* promoter upon IL-6 treatment (Fig. 4, K and L, and fig. S4K), suggesting that IL-6 signaling drives USP24 transcriptional activity to promote PD-1 abundance in T cells. RNA-seq analysis (GSE199047) revealed that IL-6 receptor knockout down-regulated *USP24* expression, but not other USP family genes, in TILs (fig. S4L), further supporting the notion that USP24 plays a critical role in maintaining PD-1 surface expression, which is transcriptionally activated by IL-6 stimulation.

### USP24-i-101 blocks USP24 enzyme activity to decrease PD-1 protein stability

**Fig. 5.**
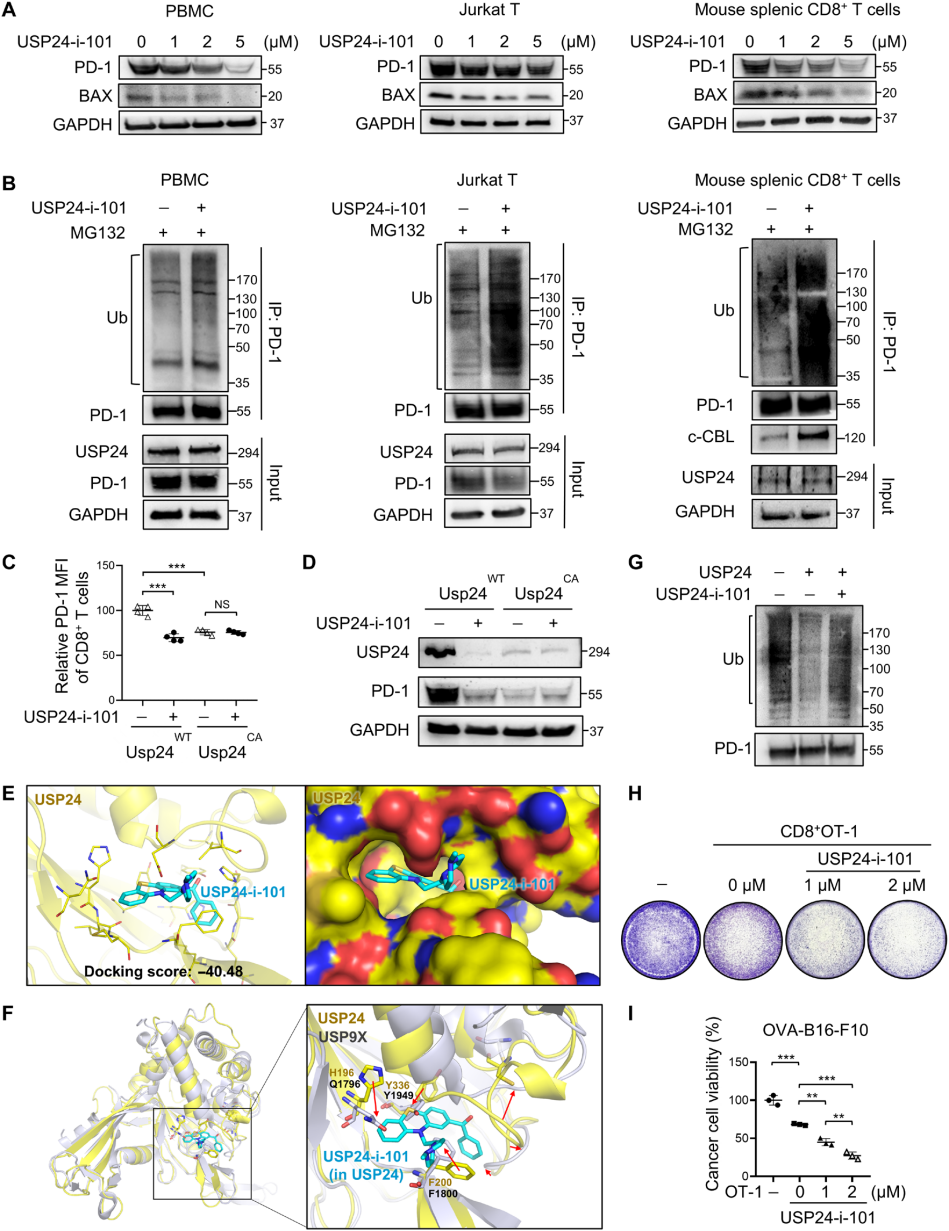
USP24-i-101 treatment down-regulates PD-1 protein stability in vitro. (**A**) PBMCs and mouse splenic CD8^+^ T cells with anti-CD3/CD28 antibody stimulation were treated with USP24-i-101 for 48 hours and Jurkat T cells were treated for 72 hours, followed by immunoblotting of the indicated proteins. (**B**) PD-1 ubiquitination levels were measured in indicated cells with USP24-i-101 treatment. (**C** and **D**) PD-1 surface levels and protein expression in splenic CD8^+^ T cells from *Usp24^WT^* or *Usp24^C1695A^* mice with or without USP24-i-101 treatment for 48 hours. MFI, mean fluorescence intensity. (**E**) Docking pose of the compound in USP24. (**F**) Binding site comparison between USP24 and USP9X, highlighting differences in residue composition, orientation, and loop shifts. (**G**) In vitro deubiquitination assays. USP24 recombinant protein was pretreated with or without USP24-i-101 for 2 hours and incubated with ubiquitinated PD-1 for an additional 2 hours. (**H** and **I**) In vitro T cell–mediated killing assays. (H) CD8^+^OT-1^+^ T cells were pretreated with indicated doses of USP24-i-101 for 48 hours and then cocultured with OVA-B16-F10 cancer cells (effector cell–to–target cell ratio, 5:1) for 24 hours. (I) Crystal violet staining and quantification results of cancer cell viability measurement. Data are the means ± SEM (*n* = 3). **P < 0.01; ***P < 0.001 (one-way ANOVA).

Our previous reports revealed that the small-molecule inhibitor USP24-i-101 specifically inhibited USP24 enzyme activity to reverse chemotherapy resistance (*20*, *28*). However, the immunomodulatory effects of USP24-i-101 remain undiscovered. To this end, we first examined whether USP24-i-101 treatment down-regulated PD-1 expression in T cells. As shown in Fig. 5A, expression of PD-1 and a well-known USP24 substrate, BAX, was down-regulated in PBMCs, Jurkat T cells, and mouse splenic CD8^+^ T cells, while *PDCD1* mRNA expression was barely affected (fig. S5A). Moreover, ubiquitinated PD-1 was elevated in various T cell lines after USP24-i-101 treatment and enhanced c-Cbl interaction with PD-1 (Fig. 5B and fig S5B), whereas USP24-i-101 failed to increase PD-1 ubiquitination (fig. S5C) and down-regulate PD-1 protein expression (Fig. 5, C and D) in CD8^+^ T cells from *Usp24^C1695A^* mice, confirming its specificity.

To further validate the specificity of USP24-i-101 on USP24, we docked compound USP24-i-101 into the binding site of USP24 and USP9X (Protein Data Bank code: 5WCH) using the software LeadIT. The docking score revealed a higher binding affinity of USP24-i-101 with the catalytic motif of USP24 than USP9X (Fig. 5, E and F, and fig. S5D). In vitro deubiquitination confirmed that USP24-i-101 inhibited the catalytic activity of purified USP24 protein (Fig. 5G). These data indicated that USP24-i-101 specifically blocks USP24 activity to decrease PD-1 stability.

We then investigated whether USP24-i-101 increased T cell anticancer activity (fig. S5E). Activated OT-1 CD8^+^ cells were pretreated with low-dose USP24-i-101, resulting in reduced PD-1 surface levels (fig. S5F). USP24-i-101 pretreatment enhanced OT-1 CD8^+^ cell activity (fig. S5G) and reduced OVA-B16-F10 cancer cell viability in a dose-dependent manner (Fig. 5, H and I). To verify that the suppressive effect of USP24-i-101 is directly attributed to decreased PD-1 levels on OT-1 T cells, we cocultured OT-1 CD8^+^ T cells with PD-L1–deficient OVA-B16-F10 cells. We observed increased tumor-killing effects when OT-1 cells were cocultured with cancer cells harboring shPD-L1. However, USP24-i-101 did not enhance the cytotoxicity of OT-1 cells against shPD-L1 cancer cells. These findings supported the notion that USP24-i-101 down-regulates PD-1 expression to enhance T cell cytotoxicity (fig. S5, H and I). In line with mouse OT-1 cells, activated human PBMCs with USP24-i-101 pretreatment also showed strong cytotoxicity against various cancer cells (fig. S5, J to M). T cells treated with USP24-i-101 displayed enhanced T cell effector functions (fig. S5N), while USP24-i-101 failed to promote T cell activity in PD-1–overexpressed T cells in the presence of PD-L1 Fc protein (fig. S5O), suggesting that USP24-i-101 increases T cell activity through inhibition of PD-1/PD-L1 signaling. Notably, USP24-i-101 exerted no apparent cytotoxicity against T cell lines but showed killing effects in both human and mouse lung cancer cell lines, albeit to different extents (fig. S5, P and Q). Together, USP24-i-101 destabilizes PD-1 and therefore enhances T cell–mediated cytotoxicity.

### USP24-i-101 boosts antitumor immunity and synergizes with anti-CTLA therapy

To examine whether USP24-i-101 provoked antitumor efficacy in vivo, we performed a Lewis lung carcinoma (LLC)-allograft model. As shown in fig. S6 (A to C), USP24-i-101 reduced tumor growth in a dose-dependent manner without altering the body weight of mice. We next investigated whether USP24-i-101 exerted immune stimulation to remodel TME. USP24-i-101 treatment decreased PD-1^+^ and exhausted CD8^+^ T cells (fig. S6, D and E) with an increase in T cell activity (fig. S6, F and G). Notably, USP24-i-101 also reduced PD-1 levels in CD4^+^ T cells (fig. S6H). The proportion of total infiltrating CD4^+^ or T_regs_ showed no difference (fig. S6, I and J), whereas the ratio of M1/M2 macrophages was increased after USP24-i-101 treatment (fig. S6K). Blood biochemistry and histological examination of major organs showed no obvious difference between dimethyl sulfoxide (DMSO)– and USP24-i-101–treated mice (fig. S6, L and M), suggesting that USP24-i-101 is a potent drug to interrupt PD-1–driven immunosuppression and provoke antitumor immunity. To verify the effects of immunomodulation by USP24-i-101, we established A549 xenograft in NOD-SCID immunodeficient mice (fig. S6N). Analysis of the end point tumors showed no significant change in tumor volume after USP24-i-101 treatment in immunodeficient mice (fig. S6, O and P), supporting the notion that USP24-i-101 inhibits tumor growth mainly by boosting antitumor immunity.

**Fig. 6.**
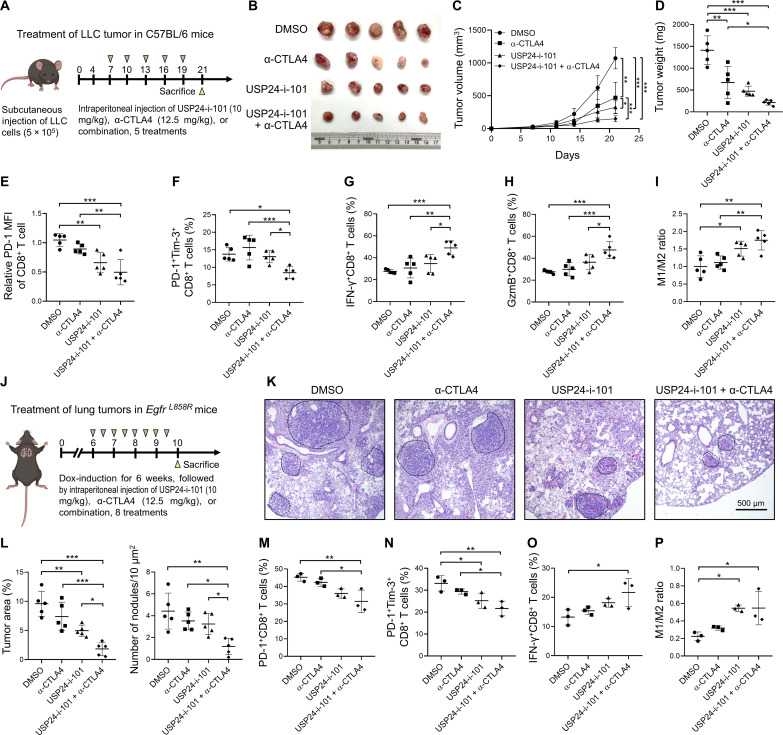
Combination of USP24-i-101 with α-CTLA4 exerts synergistic antitumor effects. (**A**) LLC-bearing C57BL/6 mice were intraperitoneally injected with DMSO, α-CTLA4 (12.5 mg/kg), USP24-i-101 (10 mg/kg), or USP24-i-101 combined with α-CTLA4 twice every week (six mice per group). (**B**) Representative end point tumor images. (**C** and **D**) The changes in tumor volume during the experimental period (C) and the final tumor weight (D) were measured. (**E** to **I**) The PD-1 mean fluorescence intensity of tumor-infiltrating CD8^+^ T cells (E) and percentage of PD-1^+^Tim-3^+^ (F), IFN-γ^+^ (G), and GzmB^+^CD8^+^ T cells (H) and population of M1/M2 ratio of TAMs (I) were analyzed in the end point tumors by flow cytometry. (**J**) *Egfr^L858R^* mice harboring lung tumors induced for 6 weeks were intraperitoneally injected with DMSO, α-CTLA4 (12.5 mg/kg), USP24-i-101 (10 mg/kg), or USP24-i-101 combined with α-CTLA4 twice every week (five mice per group). (**K**) Representative H&E staining of lung tissue images at the end point of *Egfr^L858R^* mice with indicated treatment. (**L**) Quantification of lung tumor area and tumor nodule numbers. (**M** to **P**) The proportion of tumor-infiltrating PD-1^+^ (M), PD-1^+^Tim-3^+^ (N), and IFN-γ^+^ (O) CD8^+^ T cells and M1/M2 ratio (P) were assessed by flow cytometry. Data are the means ± SEM. *P < 0.05; **P < 0.01; ***P < 0.001 (one-way ANOVA).

Because USP24-i-101 blocked PD-1/PD-L1 signaling, we then evaluated the therapeutic effects in combination therapy with anti-CTLA4 (α-CTLA4) in LLC-bearing mice (Fig. 6A). Monotherapy with α-CTLA4 modestly inhibited tumor volume, while combination therapy with α-CTLA4 and USP24-i-101 induced more potent tumor growth inhibition (Fig. 6, B to D). TIL profiling demonstrated a strong reduction of exhausted CD8^+^ T cells (Fig. 6, E and F) and enhanced recruitment of activated T cells along with increased M1/M2 macrophage ratio in tumors by combination therapy (Fig. 6, G to I), suggesting that combination therapy exhibited better immune stimulation. Populations of T_regs_, total CD8^+^ cells, and CD4^+^ T cells showed no significant changes, but a decrease in PD-1^+^CD4^+^ T cells was observed (fig. S7, A to D). Body weight, blood biochemistry parameters, and pathologic examination showed no obvious toxicity between mice with combination therapy and DMSO treatment (fig. S7, E to G).

We further evaluated the therapeutic effects of USP24-i-101 in *Egfr^L858R^*-driven lung tumors (Fig. 6J). Monotherapy with USP24-i-101 or α-CTLA4 slightly reduced *Egfr^L858R^*-driven tumor development. However, combined treatment with USP24-i-101 and α-CTLA4 significantly retarded lung tumor growth (Fig. 6, K and L, and fig. S7H). Consistent with the LLC-allograft model, combination therapy significantly decreased exhausted CD8^+^ T cells (Fig. 6, M and N) and induced activated CD8^+^ T cells and M1 macrophages (Fig. 6, O and P, and fig. S7I). However, T_reg_ population and body weight remain unchanged (fig. S7, J to L). These results indicated USP24-i-101 as a promising drug that synergizes the efficacy of α-CTLA4 immunotherapy.

### USP24^+^PD-1^+^Lag-3^+^CD8^+^ T cells predict lung cancer prognosis

**Fig. 7.**
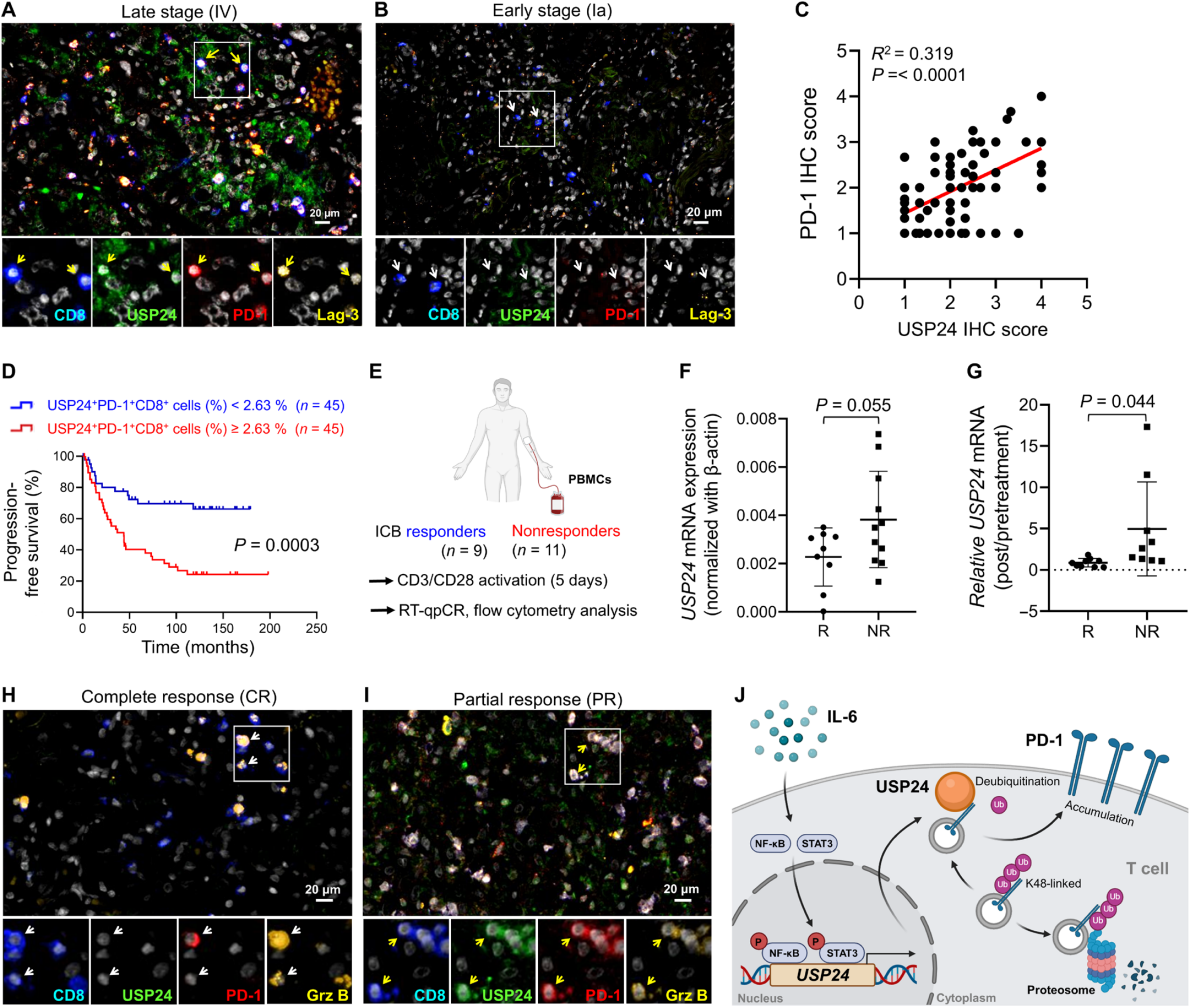
Patients with high USP24^+^ tumor-infiltrating CD8^+^ T cells show worse clinical outcomes and a poor ICB response. (**A** and **B**) IF-IHC staining of intratumoral CD8^+^ T cells (blue) with USP24 (green), PD-1 (red), Lag-3 (yellow), and DAPI (white) in tumor specimens from a patient with early-stage lung cancer (A) or a patient with late-stage lung cancer (B). (**C**) Correlation between USP24 and PD-1 expression levels in 90 lung cancer specimens. (**D**) Progression-free survival in 90 patients with lung cancer exhibiting highly or low infiltrating USP24^+^PD-1^+^CD8^+^ T cells. (**E**) Diagram showing PBMCs collected from patients with lung cancer representing R or NR. (**F**) *USP24* mRNA expression in PBMCs from R or NR patients with lung cancer (R, *n* = 9; NR, *n* = 11). (**G**) Ratio of *USP24* expression (posttreatment versus pretreatment of immunotherapy) in PBMCs from patients with lung cancer (R, *n* = 9; NR, *n* = 9). (**H** and **I**) Multiplex IF-IHC staining of tumor species from patients with lung cancer showing a complete response (CR) (H) or partial response (PR) (I) to immunotherapy (CR, *n* = 5; PR, *n* = 5). (**J**) Model illustrating that tumor environmental IL-6 up-regulates STAT3/NF-κB signaling to increase USP24 expression, thereby enhancing PD-1 deubiquitination and stability, which results in impaired T cell anticancer activity. Data are the means ± SEM. *P < 0.05; **P < 0.01; ***P < 0.001 (Student’s *t* test).

To investigate the clinical significance of USP24-promoted PD-1 expression, we performed multiplex IF-immunohistochemistry (IF-IHC) staining in surgically resected tumor specimens of 90 patients with lung cancer. We observed USP24 expression almost overlapping with PD-1 abundance in tumor-infiltrating CD8^+^ T cells (Fig. 7A). Notably, late-stage lung cancer specimens were highly infiltrated with USP24^+^PD-1^+^Lag-3^+^CD8^+^ dysfunctional T cells (Fig. 7A), while USP24 and Tex markers were barely expressed in patients with early-stage lung cancer (Fig. 7B). On the basis of multiplex IF-IHC score analysis, USP24 positively correlated with PD-1 in tumor-infiltrating CD8^+^ T cells (Fig. 7C), providing clinical validation of USP24-mediated PD-1 expression.

Furthermore, we examined whether infiltration of USP24^+^PD-1^+^CD8^+^ T cells was associated with cancer progression in lung cancer. The patients in our cohort were classified into two groups, low and highly infiltrating USP24^+^PD-1^+^CD8^+^ T cells, which depended on the respective medium value of all individuals. By Kaplan-Meier analysis, high tumor infiltration of USP24^+^PD-1^+^CD8^+^ T cells showed worse progression-free survival and overall survival rates (Fig. 7D and fig. S8A) compared to patients with low infiltrating USP24^+^PD-1^+^CD8^+^ T cells. Meanwhile, the three-parameter profile showed an increased predictive power of overall survival than the two-parameter profile (PD-1^+^CD8^+^) (fig. S8B). Cox regression analyses revealed a correlation of high intratumoral USP24^+^PD-1^+^CD8^+^ T cells, advanced tumor stage, larger primary tumor size, lymph node, or distant organ metastasis with poor survival outcomes (Table 1). Multivariate analysis revealed that a highly infiltrating USP24^+^PD-1^+^CD8^+^ T signature exhibited an increased risk of death (hazard ratio, 2.860; *P* = 0.001), even after adjusting for other clinical parameters (Table 1). In addition, highly infiltrating USP24^+^PD-1^+^CD8^+^ T cells occurred in young patients and patients with adenocarcinoma and were associated with advanced tumor stage, larger primary tumor size, and lymph node metastasis (table S1). Together, high infiltration of USP24^+^PD-1^+^CD8^+^ T signature indicates T cell dysfunction and worse clinical outcomes in lung cancer.

**Table 1.** Cox regression analysis of risk factors for cancer-related death in 90 patients with lung cancer.

Characteristics	Univariate analysis		Multivariate analysis	
	HR* (95% CI†)	*P* value‡	HR (95% CI)	*P* value‡
USP24^+^/PD-1^+^/CD8^+^ (%)§				
Low	1		1	
High	2.904 (1.528–5.519)	**0.001**	2.860 (1.501–5.447)	**0.001**
PD-1^+^/CD8^+^ (%)§				
Low	1		1	
High	1.792 (1.003–3.201)	0.049	0.882 (0.390–1.731)	0.606
Age				
<65	1		–**	
≥65	0.946 (0.533–1.681)	0.851	–**	–**
Gender				
Female	1		–**	
Male	1.843 (1.043–3.254)	0.035	–**	–**
Tumor type¶				
ADC	1		–**	
SCC	1.445 (0.647–3.225)	0.369	–**	–**
Stage				
I–II	1		1	
III–IV	2.850 (1.598–5.082)	**0.001**	1.607 (0.729–3.544)	0.240
T status#				
T1–T2	1		1	
T3–T4	2.461 (1.265–4.788)	**0.008**	1.382 (0.652–2.929)	0.398
N status#				
≤N1	1		1	
>N1	2.391 (1.326–4.312)	**0.004**	0.994 (0.308–3.224)	0.996
M status#				
M0	1		1	
≥M1	3.298 (1.460–7.451)	**0.004**	2.212 (0.913–5.360)	0.079

*HR, hazard ratio.

^†^CI, confidence interval.

^‡^Bold values indicate statistical significance (*P* < 0.05).

^§^A patient with USP24^+^/PD-1^+^/CD8^+^ cells (%) ≥2.63% was defined as high expression.

^¶^ADC, adenocarcinoma; SCC, squamous cell carcinoma.

^#^T status, primary tumor; N status, lymph node metastasis; M status, distant metastasis.

^**^The variables without significant HR in the univariate analysis were not included in the multivariate analysis.

### Elevated *USP24* mRNA in T cells predicts poor immunotherapy response

Patients with exhausted immunotype peripheral blood CD8^+^ T cells are less likely to benefit from ICB therapy (*29*, *30*). We collected PBMCs from 20 patients with lung cancer before and after receiving ICB therapy. These patients were divided into responder (R) and NR patients (Fig. 7E). As shown in Fig. 7F, the basal *USP24* mRNA level was up-regulated in PBMCs of NR patients compared to R patients. Moreover, *USP24* expression was further elevated in NR patients after ICB treatment (Fig. 7G) and highly correlated with exhaustion features (fig. S8C), suggesting that lower *USP24* levels in PBMCs benefit from ICB treatment. In addition, tumor tissue from patients with lung cancer who exhibited a complete response to ICB showed low infiltration of USP24^+^CD8^+^ T cells (Fig. 7H and fig. S8D) in comparison to partial response patients (Fig. 7I and fig. S8D). Together, elevated USP24 levels indicate PD-1 abundance and T cell dysfunction, which contributed to worse clinical outcomes and poor ICB responses in patients with lung cancer.

## DISCUSSION

Our study identifies USP24 as a novel PD-1 deubiquitinase in T cells. USP24 stabilizes PD-1 by removing K48-linked polyubiquitin and preventing proteasome-mediated degradation. Moreover, the cytokine IL-6 drives STAT3/NF-κB signaling to increase USP24 expression, which contributed to PD-1 abundance and impaired T cell anticancer effects (Fig. 7J).

Accumulated evidence underscores the pathological role of USP24 in regulating cancer cell stemness, sustained proliferation, metastasis, inhibition of ferroptosis, and drug resistance (*16*, *28*). However, its contribution to the immunomodulation within the TME remains unclear. Recently, several studies have highlighted the importance of USP24 in modulating immune activity within the TME. For instance, USP24 controls PD-L1 stability in lung cancer cells (*20*), suggesting its role in repressing T cell activity in USP24 highly expressed cancers. Moreover, we previously demonstrated that USP24 stabilizes p300 to increase IL-6 transcriptional activity in the TAMs and accelerate cancer malignancy (*19*). TAM-derived IL-6 transcriptionally activates *PDCD1* expression via STAT3 signaling in T cells (*25*). Here, we demonstrate that environmental IL-6 promotes USP24 expression, suggesting a positive loop between IL-6 and USP24. Collectively, USP24 activity predominantly establishes an immunosuppressive TME to accelerate cancer progression.

PD-1 is mainly expressed in activated T cells and various immune cells, including natural killer cells, B cells, macrophages, and dendritic cells (*2*). The PD-1/PD-L1 axis dampens CD8^+^ T cell proliferation, cytokine production, and cytotoxicity capacity by recruiting SHP-1/2 phosphatase to antagonize the T cell receptor (TCR) and CD28 costimulation signaling pathways (*31*). In CD4^+^ T helper cells, PD-1 signaling activates SHP-1/2, leading to STAT1 dephosphorylation in T helper 1 cells, which facilitates their conversion into T_regs_ (*32*) while also promoting T_reg_ proliferation and enhancing their immunosuppressive effects (*33*). In TAMs, PD-1 up-regulation impairs their phagocytic capability against tumor cells, while PD-1 blockade restores macrophage phagocytosis to reduce tumor growth (*34*). Our study demonstrated that USP24 promotes PD-1 protein stability in both CD4^+^ and CD8^+^ T cells and also increases levels on TAMs, suggesting USP24 as a universal regulator in PD-1 expression. Whether USP24 plays an additional role in regulating the phagocytic capacity of TAMs and T_reg_ immunosuppressive function to modulate TME is worthy of further investigation.

The ubiquitin-proteosome system tightly controls PD-1 at different stages after protein synthesis. Potential ubiquitination sites on PD-1 have been identified at lysine-78 (K78), K210, and K233. Several E3 ligases such as MDM2, KLHL22, FBXO38, FBW7, and c-Cbl mediate the ubiquitination of PD-1 (*35*). Augmenting the expression or catalytic efficacy of these E3 ligases targeting PD-1 potentially boosts T cell activity against malignancies (*36*). In addition to ubiquitination, other PTMs, such as N-linked glycosylation, mediate PD-1 stability and reinforce binding affinity for ligand PD-L1 (*37*). Targeting this modification through oligosaccharyltransferase inhibitors, like NGI-1, reduces PD-L1 or PD-1 glycosylation and thus increases sensitivity to immunotherapy (*38*). Phosphorylation is another crucial PTM for PD-1 function and T cell activity. Src-family kinases phosphorylate PD-1 at tyrosine residues Y223 and Y248, facilitating SHP-2 recruitment, which impairs TCR activation and CD28 signaling, ultimately causing T cell dysfunction and immunotherapy failure (*39*). SHP-2 inhibition by SHP099 augments antitumor immunity and shows a higher therapeutic efficacy with α-PD-1 (*40*). A recent study identifies that UFMylation of PD-1 by UFL1 antagonizes ubiquitination-mediated degradation, stabilizing PD-1 and resulting in CD8^+^ T cell dysfunction (*41*). Moreover, palmitoylated PD-1 promotes trafficking to the recycling endosome for greater presentation, thus preventing lysosome-dependent degradation (*42*). Recently, USP5 has been reported to stabilize PD-1 by extracellular signal–regulated kinase–mediated T234 phosphorylation of PD-1. Inhibition of ERK interrupts USP5-mediated PD-1 stability, enhancing the efficacy of anti-PD-1 and anti-CTLA therapy in cancer treatment (*15*). However, specific drugs targeting USP5-mediated deubiquitination remain elusive.

Accumulating evidence suggests that targeting USPs confers a susceptibility to anticancer drugs (*17*). We previously generated the parental USP24-i (NCI677397), which specifically inhibits the enzyme activity of USP24 but not other similar USPs, USP9X and USP7, determined by in vitro enzyme assay (*18*). We have used a structure-based docking assay, based on NCI677397, to screen the better analog, USP24-i-101 (NCI67708), with stronger binding affinity to the USP24 catalytic motif and lower affinity to USP9X (*20*). The difference in residue composition, orientation, and loop shifts generated steric hindrance to reduce the interaction of the compound with USP9X. As shown in Fig. 5D, USP24-i-101 treatment decreases USP24 protein expression in T cells. However, USP24 expression is not down-regulated by USP24-i-101 in the presence of the proteasome inhibitor MG132 (Fig. 5B). These findings suggest that USP24-i-101 may impair USP24 protein stability, implying that USP24 could control its own protein stability. We previously reported that USP24 stabilizes the bromodomain-containing protein BRG1, which, in turn, enhances USP24 transcriptional activity, forming a positive feedback loop (*22*). Therefore, further in-depth studies are required to address whether USP24 regulates its own protein stability.

Our study introduces an innovative therapeutic approach in which USP24-i-101 synergizes the efficacy of α-CTLA4 immunotherapy. Lower side effects are evident by in vitro cell viability assay in T cells and pathological examination of major organs from USP24-i-101–treated mice in vivo. These results suggest that USP24-i-101 is a potent agent for cancer immunotherapy. USP24-i-101 inhibits PD-1 in T cells and PD-L1 in cancer cells (*20*) and subsequently inflames antitumor immunity within the TME. Exploring the combination treatment of USP24-i-101 and anti-PD-1/PD-L1 therapy, or with other immune checkpoint inhibitors, is valuable to elucidate the therapeutic potential of USP24-i-101.

The treatment results of α-IL-6 reducing PD-1 expression in tumor-infiltrating CD8^+^ T cells (Fig. 4F) demonstrated the therapeutic potential of α-IL-6. IL-6 has been shown to play a predominant role in dampening anticancer immunity. For example, high plasma levels of IL-6 in patients with advanced kidney, breast, and bladder cancer indicate impaired effector differentiation of CD8^+^ cytotoxic T cells and a poor response to atezolizumab (α-PD-L1) therapy (*43*). Further evidence shows that IL-6 neutralization improves tumor rejection when combined with ICB and simultaneously alleviates ICB-induced immune-related adverse events (*44*). The potential of α-IL-6 for enhancing T cell function warrants further investigation.

In conclusion, we reveal that IL-6/STAT3/NF-κB transcriptionally activates USP24 to drive USP24-mediated deubiquitinating machinery to stabilize PD-1 and suppress T cell activity. We develop the inhibitor USP24-i-101 that perturbs PD-1/PD-L1 signaling to reactivate T cell functions in the TME. In addition, USP24 expression in PBMCs or tumor specimens from patients with lung cancer serves as a potential biomarker to facilitate the selection of R patients before receiving ICB therapy.

## MATERIALS AND METHODS

### Cell lines and culture condition

Human lung cancer cells H1299, A549, and H460; human embryonic kidney cells (HEK293); and mouse lung cancer cells LLC purchased from American Type Culture Collection were maintained in Dulbecco’s modified Eagle’s medium (Gibco, Grand Island, NY). OVA-B16-F10 melanoma cells were a gift from P.-C. Ho (Department of Oncology, University of Lausanne, Ludwig Institute for Cancer Research, Lausanne, Switzerland) and were maintained in DMEM. The human Jurkat T cell line and mouse OT-1 CD8^+^ T cells provided by C.-P. Chang (Department of Microbiology and Immunology, College of Medicine, National Cheng Kung University, Taiwan) and K.-J. Liu (Institute of Cancer Research, National Health Research Institutes, Taiwan) were maintained in RPMI media (Gibco). Human PBMCs were isolated from the blood of healthy donors or patients with lung cancer using Histopaque solution (no. 10771; Sigma-Aldrich, Louis, MO) and activated with anti-CD3/CD28 antibody (1 μg/ml; nos. 566685 and 555725; BD Bioscience, Franklin Lakes, NJ) for 72 hours in RPMI media. All culture media were supplemented with 10% fetal bovine serum (Gibco) and 1% penicillin/streptomycin (Gibco). All cells were incubated at 37°C in a humidified incubator containing 5% CO_2_ in air.

### Animal models

To generate C57BL/6J-NarI-*USP24^C1695A^* catalytic mutant mice, we used the CRISPR/Cas9 system to create one mutation residue from cysteine to alanine at the 1695th amino acid on the *Usp24* gene. The detailed methods have been described in a previous report (*20*). Lung-specific Usp24^C1695A^ mice were generated by crossing Tet-on *EGFR^L858R^* B6 transgenic mice (*Scgb1a1-rtTA/TetO-EGFR^L858R^*) with *Usp24^C1695A^* mice. The triple transgenic mice *(Scgb1a1-rtTA/EGFR^L858R^/Usp24^C1695A^*) were fed with doxycycline (0.5 g/liter) for 10 weeks to induce lung adenocarcinoma formation. The tumor growth was monitored using micro-CT (microcomputed tomography; SKYSCAN1276, Bruker, Middlesex, MA). For the allograft model, 6-week-old male B6 mice (obtained from National Laboratory Animal Center) were subcutaneously implanted with 5 × 10^5^ LLC cells. When the tumor volume reached around 50 mm^3^, the mice were randomly assigned six mice per group and injected with DMSO, USP24-i-101, or α-CTLA-4 (no. BE0131; BioXCell, Lebanon, NH) by intraperitoneal injection. The mouse weight and the volume of the allograft were measured and quantified during the experiment. Mice were euthanized at the end point of the experiment, and tumor tissues were resected, fixed, and embedded in paraffin for histologic hematoxylin and eosin (H&E) staining. To obtain a single-cell suspension, the tissues were digested with collagenase (0.1 mg/ml; Sigma-Aldrich) and dispase II (1 mg/ml; Sigma-Aldrich) in serum-free DMEM for 30 min at 37°C and then crushed through the mesh. The single-cell suspension was then subjected to flow cytometry (CytoFLEX, Beckman Coulter, Brea, CA) to analyze the profiling of TILs.

### Flow cytometry analysis

To determine the presentation of PD-1 and the activity of tumor-infiltrating lymphocytes, tumor tissues were stained with anti-mouse CD8, CD4, and PD-1 antibodies. All antibodies were diluted as 1:200 with staining buffer. Cells were stained and incubated on ice for 30 min. For nuclear protein, cells were fixed with 2% paraformaldehyde for 5 min and incubated in the permeabilized buffer for 10 min. Cells were then stained with granzyme B antibodies (1:200) in a permeabilized buffer for an additional 30-min incubation on ice. After washing once, cells were resuspended in staining buffer and subjected to flow cytometry analysis (CytoFLEX). The details of antibodies used for flow cytometry analysis are listed in table S2.

### Method of USP24 inhibitor synthesis

The synthesis of USP24-i-101 (NCI 677-08) was entrusted to W.-C. Wang and H.-Y. Lee (Institute of Biotechnology and Pharmaceutical Research, National Health Research Institutes). Commercially available (4-chloro-3-nitrophenyl)(phenyl)methanone (compound 1) was processed in 10 steps with an 11% overall yield. The detailed synthesis method is described here. 2-Fluorothiolphenol and *N*,*N*-diisopropylethylamine were added to compound 1 in ethanol and stirred at 80°C for 1 hour to obtain [4-(2-fluorophenyl)sulfanyl-3-nitrophenyl]-phenylmethanone (compound 2) at a yield of 94%. Na_2_S_2_O_4_ was added to compound 2 in tetrahydrofuran/H_2_O/methanol and stirred at room temperature for 16 hours to obtain [3-amino-4-(2-fluorophenyl)sulfanylphenyl]-phenylmethanone (compound 3) at a yield of 97%. Next, formic acid was added to compound 3 and stirred at 97°C for 17 hours to give *N*-[5-benzoyl-2-(2-fluorophenyl) sulfanylphenyl]formamide (compound 4) at a yield of 99%. K_2_CO_3_ in *N*,*N*′-dimethylformamide was added to a solution of compound 4 at 105°C and stirred for 9 hours to give 10*H*-phenothiazin-2-yl(phenyl)methanone (compound 5) at a yield of 78%. 2-(2-Phenyl-1,3-dioxolan-2-yl)-10*H*-phenothiazine (compound 6) was synthesized from compound 5 by Dean-Stark condensation at 130°C for 19 hours with ethanediol and toluene-4-sulfonic acid in toluene. The mixture was washed with aqueous NaOH, water, and brine, then dried over MgSO_4_, concentrated, and purified for a yield of 66%. Then, 2-chloroacetyl chloride was added to compound 6 in toluene at 0°C, stirred at 80°C for 16 hours, cooled to room temperature, concentrated, and purified to obtain 2-chloro-1-(2-(2-phenyl-1,3-dioxolan-2-yl)-10*H*-phenothiazin-10-yl)ethan-1-one (compound 7) at a yield of 84%. Borane-tetrahydrofuran complex solution was added to compound 7 in dry tetrahydrofuran at 0°C, gradually warmed to room temperature, and stirred for 19 hours. After the mixture was cooled to 0°C, methanol was added dropwise and stirred for 10 min. The mixture was concentrated and purified, giving 10-(2-chloroethyl)-2-(2-phenyl-1,3-dioxolan-2-yl)-10*H*-phenothiazine (compound 8) at a yield of 58%. Compound 8 was dissolved in chlorohydric acid in dioxane and stirred at room temperature for 22 hours. The mixture was concentrated and diluted with dichloromethane, washed with water and sodium bicarbonate, dried over MgSO_4_, and concentrated to dryness to give crude (10-(2-chloroethyl)-10*H*-phenothiazin-2-yl)(phenyl)methanone (compound 9) at a yield of 113%. Crude compound 9, KI, and 1-methylpiperazine in *N*,*N*′-dimethylformamide were stirred at 110°C for 22 hours. The reaction was quenched by adding NH_4_Cl(aq) under ice, diluted with ethyl acetate, washed with brine, dried over MgSO_4_, concentrated, and purified to give (10-(2-(4-methylpiperazin-1-yl)ethyl)-10*H*-phenothiazin-2-yl)(phenyl)methanone (compound 10) at a yield of 50%. Oxalic acid was added to a solution of compound 10 in ether and stirred at room temperature for 3 hours. The solid was filtered, washed with ether, and dried by vacuum to give (10-(2-(4-methylpiperazin-1-yl)ethyl)-10*H*-phenothiazin-2-yl)(phenyl) methanone oxalate (compound 11, NCI677-08) at a yield of 99%.

### Plasmid transfection and lentivirus infection

Plasmids used in the study are listed in table S3. For Jurkat T cells, the plasmid was transduced using IT-J reagent (Mirus Bio, Madison, WI) according to the manufacturer’s protocol. For lentivirus preparation, HEK293T cells were seeded into a 6-cm plate, transfected with DNA mixture (pCMV-ΔR8.91: 2.25 μg per plate; pMDG: 0.25 μg per plate; shRNA: 2.5 μg per plate) (RNAi core facility of Academia Sinica, Taiwan) using Turbofect reagent (Thermo Fisher Scientific, Waltham, MA) for 24 hours, then replaced with fresh medium, and incubated for 24 hours. Lentivirus-containing supernatants were collected and infected Jurkat T cells or PBMCs in 1 ml of RPMI medium containing 10 μg of polybrene (no. H9268, Sigma-Aldrich). After 48-hour infection, cells were selected with puromycin (0.5 μg/ml) for an additional 48 hours, and knockdown efficiency was measured by Western blot assay.

### Western blot assay

Cells were lysed using the 1× radioimmunoprecipitation assay buffer and centrifuged at 13,200 rpm at 4°C for 15 min. Equal amounts of protein (50 μg of total protein) were separated by 8% SDS–polyacrylamide gel electrophoresis and electroblotted onto Immobilon-P membranes (Millipore, Bedford, MA) in a transfer buffer. Membranes were blocked with 5% fat-free milk in 1× tris-buffered saline with 0.1% Tween 20 for 1 hour at room temperature. The protein was identified by incubating the membrane with primary antibodies followed by horseradish peroxidase–conjugated secondary antibodies and enhanced chemiluminescence solution (Millipore). Antibodies used for Western blotting are listed in table S2.

### Immunoprecipitation

Cells were lysed with 1× IP buffer (50 mM tris-HCl, pH 7.5, 150 mM NaCl, 20 mM α-glycerol-phosphate, 1% NP-40, and 5 mM EDTA), 500 μg of protein lysates was incubated with 2 μg of the indicated antibodies at 4°C for 1 hour, 40 μl of Protein G PLUS/Protein A mixture magnetic beads (no. LSKMAGAG10, Merck, Rahway Valles, NJ) was added to the protein-antibody mixture, and the mixture was incubated at 4°C overnight. Immune complexes were washed three times with 1× IP buffer. Proteins were eluted using 1× loading buffer, separated by 8% SDS–polyacrylamide gel electrophoresis, and then immunoblotted with indicated antibodies (table S2).

### RNA extraction and reverse transcription qPCR assay

Total RNA was extracted using a Quick-RNA Miniprep Kit (ZYMO Research, Irvine, CA). Purified RNA was converted into cDNA, followed by reverse transcription qPCR (RT-qPCR) analysis using SYBR Green Master Mix. Results were normalized to the expression level of housekeeping gene β-*actin*. Primers used for RT-qPCR analysis are described in table S4.

### Multiplex IF

A total of 1 × 10^4^ Jurkat T cells was concentrated on a slide using cytospin at 700 rpm for 5 min and then fixed with 2% formaldehyde for 15 min. The Opal stain kit (no. NEL810001KT, Akoya Biosciences, Marlborough, MA) was used according to the manufacturer’s instructions. Antigen retrieval was conducted using the microwave at 100% power for 3 to 4 min, followed by 20% power for 20 min. Samples were allowed to cool down at room temperature for 20 min. After blocking for 10 min, samples were covered with a primary antibody and incubated at 4°C overnight or for 1 hour depending on the antibody. The next day, samples were incubated with the polymer horseradish peroxidase for 10 min and then Opal fluorophore for 10 min at room temperature. For staining of another primary antibody, the microwave steps were repeated. 4′,6-Diamidino-2-phenylindole (DAPI) was then applied for nucleus staining. The antibody conditions are described in table S2.

### PD-L1 binding assay

shC or shUSP24 Jurkat T cells were stimulated with phorbol 12-myristate 13-acetate (PMA)/ionomycin for 6 hours followed by concentrating on the slide using cytospin and then fixing with 2% formaldehyde for 15 min. Cells were incubated with human PD-L Fc chimera protein (5 μg/ml; no. 156-B7; R&D Systems, Minneapolis, MN) for 2 hours at room temperature. After washing with tris-buffered saline with 0.1% Tween 20, cells were subsequently incubated with anti-human immunoglobulin G (IgG) antibody (no. GTX27149; Gentex) for 2 hours at room temperature. The green signal was visualized and captured using an Olympus BX43 microscope.

### CHX chase assay

shC or shUSP24 Jurkat T cells activated with PMA/ionomycin for 4 hours and HEK293 cells transfected with GFP-EV, GFP-USP24(WT), or GFP-USP24(C1698A) plasmids for 24 hours were treated with the protein synthesis inhibitor CHX (50 μg/ml) for the indicated time points. Cell lysates were subject to Western blotting for analyzing the PD-1 protein half-life.

### In vitro deubiquitination assay

To enrich ubiquitinated PD-1, Jurkat T cells were treated with PMA/ionomycin and MG132 (10 μM) for 6 hours simultaneously. PD-1 protein in cell lysate was immunoprecipitated with anti-PD-1 antibody for 4 hours followed by incubating with magnetic beads for 2 hours. Isolated PD-1 proteins were reacted with or without human USP24 recombinant protein (50 μg/ml) (Origene) in deubiquitination buffer (50 mM tris-HCl, pH 8, 150 mM NaCl, 5 mM MgCl_2_, and protease inhibitor) for 6 hours at 37°C. The reaction was stopped by adding 1× sample buffer, and PD-1 ubiquitination levels were measured by Western blotting.

### In vitro T cell killing assay

OT-1 CD8^+^ T cells were isolated from spleens of OT-1 TCR transgenic mice using a biotin mouse CD8 T lymphocyte enrichment set (51-9000830, BD Bioscience) and stimulated with anti-CD3/CD28 antibodies (1 μg/ml) and IL-2 (10 ng/ml) for 24 hours. Subsequently, OT-1 CD8^+^ T cells (1 × 10^5^) were infected with lentiviruses expressing shUsp24 or shC in RPMI medium containing polybrene (10 μg/ml) for 48 hours. OT-1 CD8^+^ T cells were incubated with preseeded OVA-B16-F10 cancer cells in 24-well plates at a ratio of 5:1 (effector to target cells) for 24 hours. Cancer cells were fixed with 2% paraformaldehyde, stained with 0.1% crystal violet, and then photographed and quantified to determine cancer cell viability using ImageJ software. For the human PBMC coculture system, PBMCs (1 × 10^5^) activated with anti-CD3/CD28 antibodies (1 μg/ml) were cocultured with H1299, H460-luc, and A549-luc lung cancer cells (2 × 10^4^) for 24 hours. For H1299 cells, cells were fixed with 2% paraformaldehyde, stained with 0.1% crystal violet, and then photographed and quantified to determine cancer cell viability using ImageJ software. For H460-luc and A549-luc cells, cell viability was determined by luciferase assays using the Dual-Glo Luciferase Assay System (Promega, Madison, WI).

### Cell viability assay

Human and mouse lung cancer cells, as well as human T cells, were seeded in 96-well plates and treated with the indicated dose of USP24-i-101 in 100 μl of culture medium. After 48-hour incubations, cells were incubated in medium containing CCK-8 buffer (no. CK04; Dojindo Laboratories, Kumamoto, Japan) according to the manufacturer’s protocol for 1 hour, and absorbance at 450 nm was measured.

### ChIP assay

Jurkat T cells (5 × 10^6^) were cross-linked with 1% formaldehyde followed by the preparation of nuclear lysates using a Magna ChIP Protein G Kit (Millipore). Nuclear lysates were sonicated to shear DNA to around 500 base pairs followed by IP for 16 hours at 4°C using IgG and anti-p-p65 or anti-p-STAT3 antibody. The levels of targeted genes in ChIP products were determined using RT-qPCR. The primers used are listed in table S4.

### RNA-seq and GSEA

Total RNA was extracted from lung tumors of *Egfr^L858R^/Usp24^WT^* or *Egfr^L858R^/Usp24^C1695A^* mice and purified using a Quick-RNA MiniPrep Kit (Zymo Research, Irvine, CA). For RNA-seq analysis, 3 μg of isolated total RNA was qualified and sequenced by Biotools Biotech Co., Ltd. (Taipei City, Taiwan). The detailed methods were described in a previous report (*28*), and the raw sequence data have been deposited in the Gene Expression Omnibus database under the accession code GSE281983. Differentially expressed genes (DEGs) were identified using the DESeq R package version 1.12.0, with the significance of the gene expression difference indicated by an adjusted *P* value <0.05. Subsequently, DEGs were further annotated using Gene Ontology and Kyoto Encyclopedia of Genes and Genomes databases. For GSEA analysis, the DEG data were subjected to the Molecular Signature Database (MSigDB; version 5.0) and pathways were filtered and displayed when the *P* value <0.01.

### Clinical samples of patients with lung cancer

To detect *USP24* expression in patient-derived PBMCs, 24 patients with lung cancer were recruited from the National Cheng Kung University Hospital. Patient PBMCs were isolated from peripheral blood by density gradient centrifugation using Histopaque solution and activated by the anti-CD3/CD28 antibody (1 μg/ml) for 5 days in RPMI medium. Cells were used for RT-qPCR analysis of *USP24* expression or detection of PD-1 and Tim-3 using flow cytometry.

To measure the level and localization of USP24^+^ dysfunctional CD8^+^ T cells in tumor specimens, 90 surgically resected patients with lung cancer and 10 patients with lung cancer exhibiting a complete or partial response to ICB treatment were recruited from National Cheng Kung University Hospital. The clinical features of the patients are listed in tables S5 to S7. Multiplex IF-IHC was performed to detect the protein level of USP24, PD-1, and Lag-3 and localization of CD8^+^ T cells in tumor specimens. Quantification of IF-IHC was defined by an average of immunoreactive positive cells per three regions of interest (695 by 695 nm). The value of 2.63% was used as a cutoff for correlation analyses. The images were captured using an Olympus BX43 microscope to analyze the immunoreactivity and colocalization signals. The antibodies and experimental conditions are listed in table S2. Overall survival was calculated from the day of surgery to the date of death or the last follow-up. Tumor typing and disease staging were performed according to the World Health Organization and TNM classifications, respectively. Information on the age, sex, and smoking history of the patients was obtained from hospital records.

### Statistics

All in vitro experiments were performed with at least three biological replicates, and the animal experiments were performed with at least two biological replicates. Two-tailed Student’s *t* test or one-way analysis of variance (ANOVA) using GraphPad Prism 8.0.1 was performed in cell and animal studies. The statistical analyses of the level of intratumoral CD8^+^ T cells with patients’ survival time were performed using Statistical Package for the Social Sciences (SPSS) version 26.0 (SPSS Inc., Headquarters Chicago, IL). Overall survival was calculated according to the Kaplan-Meier method by the log-rank test. Correlations were examined using Pearson’s correlation test. Cox regression comparison was performed to analyze the relative risk for poor outcomes in patients. Data represent the means and SEM. The levels of statistical significance were shown as *P* values: *P < 0.05; **P < 0.01; ***P < 0.001.

### Study approval

All animal experiments were performed in compliance with National Cheng Kung University institutional guidelines for the use and care of animals (permit no. 112004). Human specimens were supplied by National Cheng Kung University Hospital after obtaining appropriate institutional review board permission and informed consent from the patients (permit nos. B-ER-110-104, B-ER-106-028, and B-ER-105-406).
