# Supplementary material for: Deubiquitinase USP24 activated by IL-6/STAT3 enhances PD-1 protein stability and suppresses T cell antitumor response
Source: Sci Adv. 2025 Apr 16;11(16):eadt4258. doi: 10.1126/sciadv.adt4258 (PMC12002121; doi:10.1126/sciadv.adt4258)

Science Advances
AAAS

Supplementary Materials for

Deubiquitinase USP24 activated by IL-6/STAT3 enhances PD-1 protein stability and suppresses T cell antitumor response

Hung-Chia Hsieh *et al.*

Corresponding author: Jan-Jong Hung, petehung@mail.ncku.edu.tw; Yi-Ching Wang, ycw5798@mail.ncku.edu.tw

Sci. Adv. **11**, eadt4258 (2025)
DOI: 10.1126/sciadv.adt4258

This PDF file includes:

Figs. S1 to S8
Tables S1 to S7
Data S1

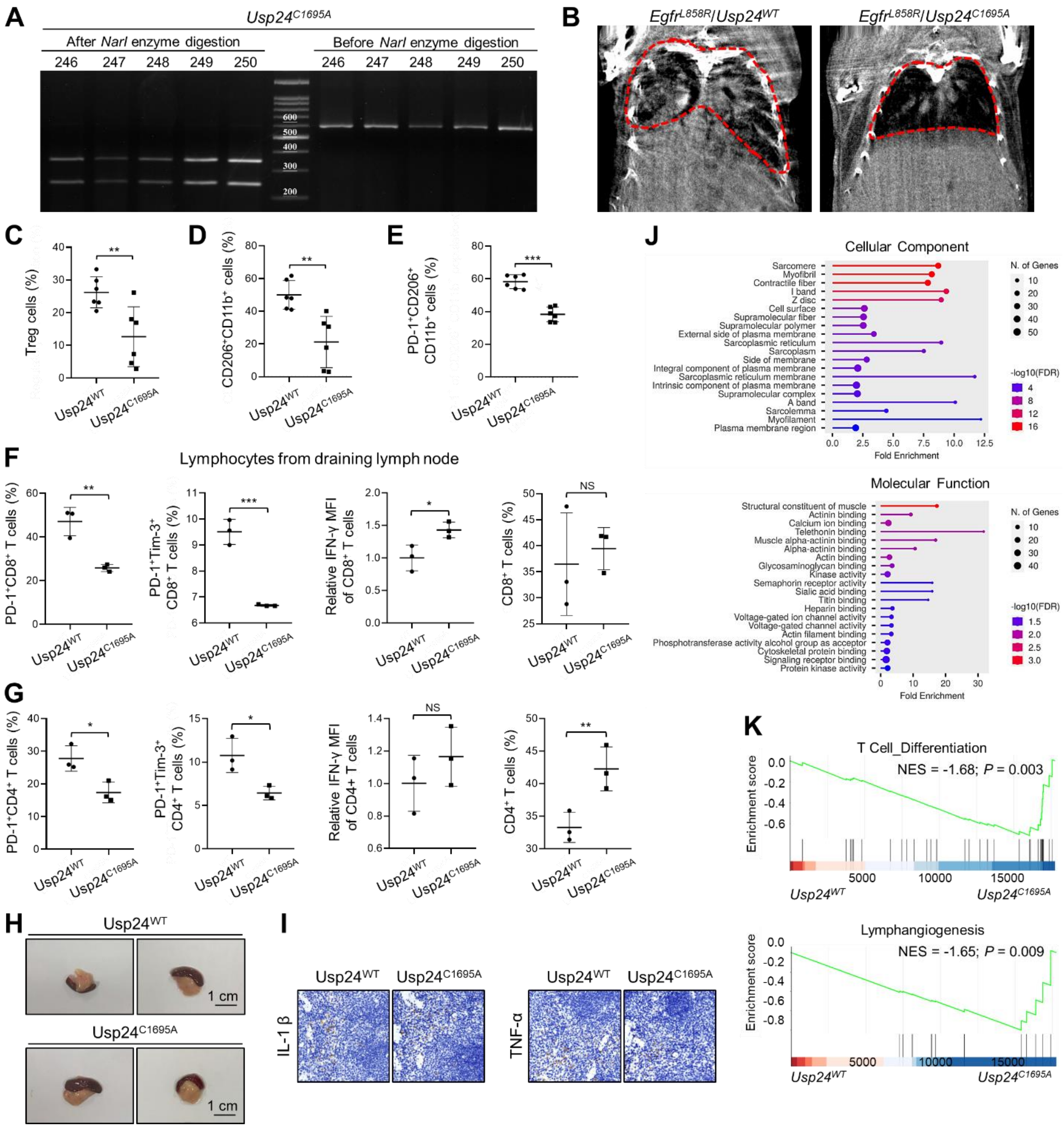

Figure S1. The effects of USP24 on tumor formation and T cell development. (A) The *Usp24* gene was edited using CRISPR-Cas9 to mutate the 1695th amino acid (cysteine to alanine). A new restriction site, *NarI,* was added for genotyping *Usp24^{C1695A}* mice. **(B)** Represented images of lung tumors. **(C to E)** Population of immunosuppressive tumor-infiltrating regulatory T cells (Tregs, $CD4^+CD25^+Foxp3^+$) (C) and PD-1$^+$ tumor-associated macrophages ($CD206^+$ $CD11b^+$) (D and E) from *Egfr^{L858R}/Usp24^{WT}* or *Egfr^{L858R}/Usp24^{C1695A}* mice with 10 weeks of doxycycline induction. **(F and G)** Draining lymph nodes of *Egfr^{L858R}/Usp24^{WT}* or *Egfr^{L858R}/Usp24^{C1695A}* tumor-harboring mice were isolated and stimulated with anti-CD3/CD28 for 24 h. (F) The percentages of the PD-1$^+$, PD-1$^+$ Tim-3$^+$, and relative IFN-γ MFI of CD8$^+$ or (G) CD4$^+$ T cells were determined by flow cytometry. **(H and I)** Representative spleen images (H) and IHC staining of IL-1 β and TNF-α in the spleen (I) from *Usp24^{WT}* or *Usp24^{C1695A}* mice. **(J)** The top 20 enriched cellular components and molecular functions were analyzed by the ShinyGO 0.80 website. **(K)** GSEA analysis shows upregulation of lymph node development, T cell differentiation, and lymphangiogenesis in lung tumors of *Egfr^{L858R}/Usp24^{C1695A}* mice. Data are mean ± s.e.m. *, $P < 0.05$; **, $P < 0.01$; ***, $P < 0.001$ (Student's t-test).

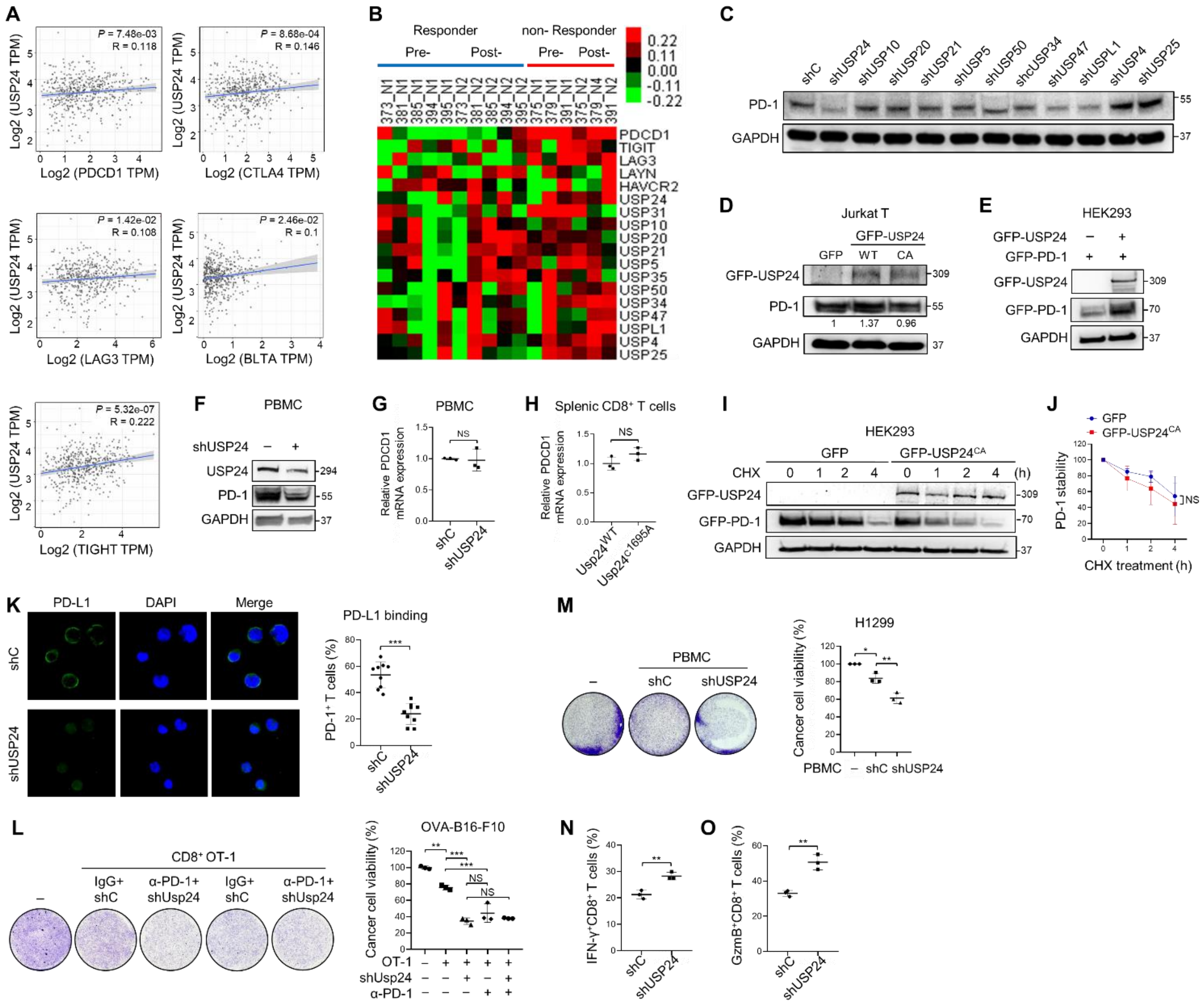

Figure S2. USP24 inhibition decreases PD-1-mediated immunosuppression. (A) Association of exhaustion T cell gene signatures with USP24 in lung cancer patients using TIMER2.0 database. **(B)** Heatmap depicting expression level of T cell exhaustion markers and USP family genes in PD-1 blockade responder and non-responder patients. Data were from the GSE111414 database. **(C)** PD-1 protein levels in Jurkat T cells with sh-knockdown of the indicated USP family members. **(D)** Jurkat T cells were transfected with USP24-WT or USP24-C1698A plasmid and analyzed for PD-1 expression by Western blotting. **(E)** HEK293 cells were ectopically expressed with GFP-USP24 and GFP-PD-1. **(F and G)** Immunoblotting (F) or RT-qPCR (G) to measure PD-1 protein and mRNA expression in shC or shUSP24 transduced peripheral blood mononuclear cells (PBMCs). **(H)** *PDCD1* mRNA expression in anti-CD3/28 stimulated splenic $CD8^{+}$ T cells from $Usp24^{WT}$ or $Usp24^{C1695A}$ mice. **(I and J)** CHX chase assay was performed in HEK293 cells ectopically expressing GFP-PD-1 along with GFP or GFP-USP24-CA (I). Quantification of GFP-PD-1 protein expression in cells with indicated time points of CHX treatment (J). **(K)** shC and shUSP24 Jurkat T cells were incubated with PD-L1 Fc protein for 1 h and then with anti-human Alexa Fluor 488 dye for 1 h. Cells were subjected to IF assays to detect PD-L1 binding intensity. **(L)** *In vitro* T cell-mediated killing assays. $CD8^{+}OT\text{-}1^{+}$ T cells were introduced with shC or shUsp24 lentiviruses and treated with IgG or α-PD-1 (#BE0146; 2 μg/ml) for 48 h, followed by co-culturing with OVA-B16F10 cancer cells (E:T ratio = 3:1) for an additional 24 h. Crystal violet staining for cancer cell viability measurement (*left*). Quantification using image J software (*right*). **(M)** PBMCs were transduced with shC or shUSP24 lentiviruses and then co-cultured with H1299 lung cancer cells (E:T ratio = 5:1) for 24 h. Crystal violet staining (*left*) and cancer cell viability measurement (*right*). **(N and O)** Detection of IFN-γ^{+} (N), $GzmB^{+}$ (O) $CD8^{+}$ T cells in shC or shUSP24 PBMCs by flow cytometry after co-culturing with H1299 cells. Data are mean ± s.e.m. (n=3). *, $P < 0.05$; **, $P < 0.01$; ***, $P < 0.001$ (one-way ANOVA).

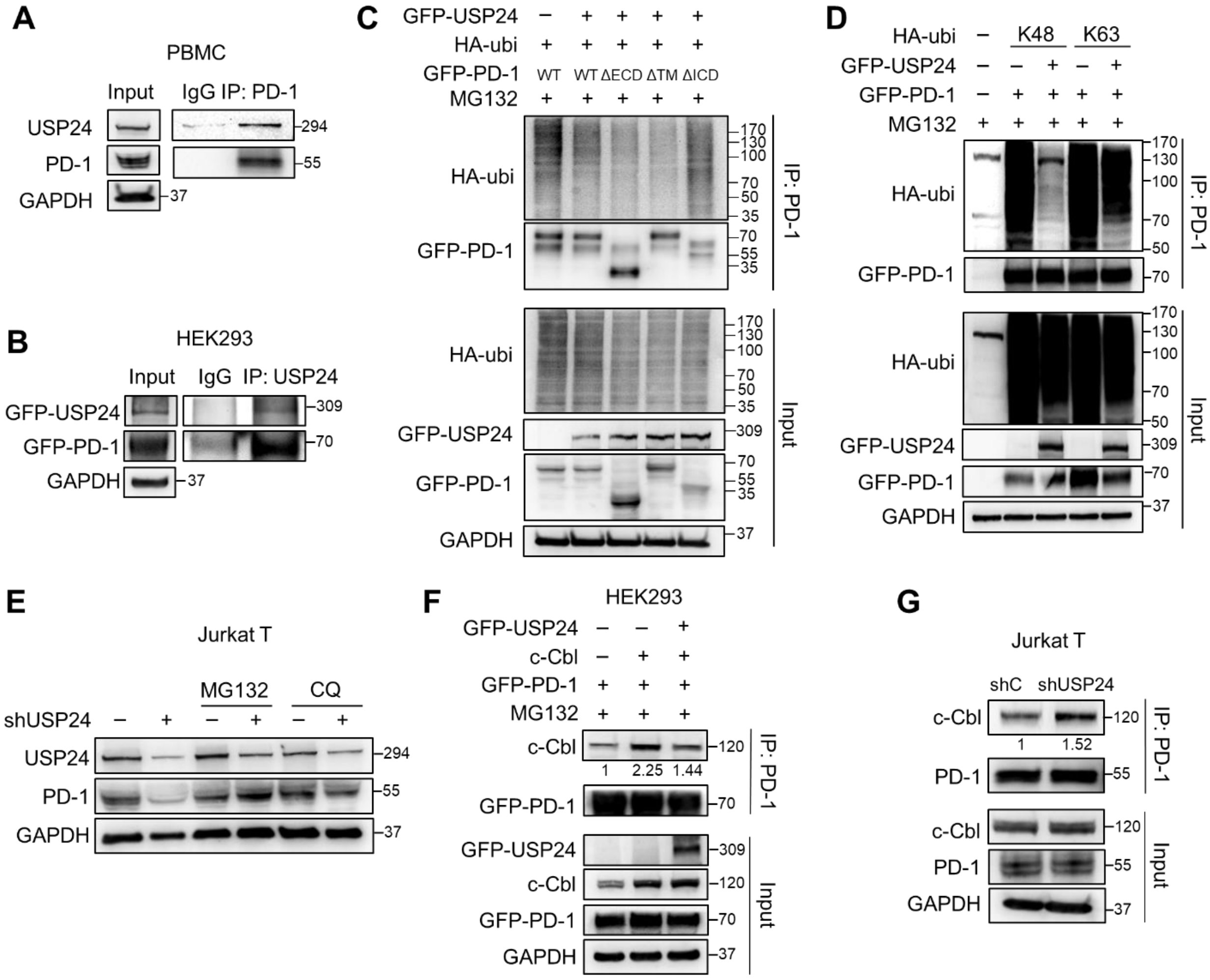

Figure S3. USP24 counteracts with the interaction of c-Cbl and PD-1. (A and B) IP of endogenous PD-1 in PBMCs (A) and exogenous USP24 in HEK293 cells with ectopically overexpressed GFP-USP24 and GFP-PD-1 (B). **(C)** HEK-293 cells were transduced with HA-ubiquitin and WT-USP24, along with indicated PD-1 constructs, followed by the detection of PD-1 ubiquitination by IP-Western analysis. **(D)** HEK-293 cells were transfected with PD-1 and K48 or K63 ubiquitin with or without WT-USP24 and analyzed for PD-1 ubiquitination by IP analysis. **(E)** Immunoblotting of PD-1 in shC or shUSP24 Jurkat T cells treated with 10 μM of MG132 or 20 μM of chloroquine (CQ) for 6 h. **(F)** IP of PD-1 followed by immunoblotting with c-Cbl in HEK-293 cells transfected with empty vector/PD-1, empty vector/c-Cbl/PD-1, or USP24/c-Cbl/PD-1. **(G)** IP of PD-1 followed by immunoblotting with c-Cbl in shC or shUSP24 Jurkat T stimulated with PMA/Ionomycin for 6 h.

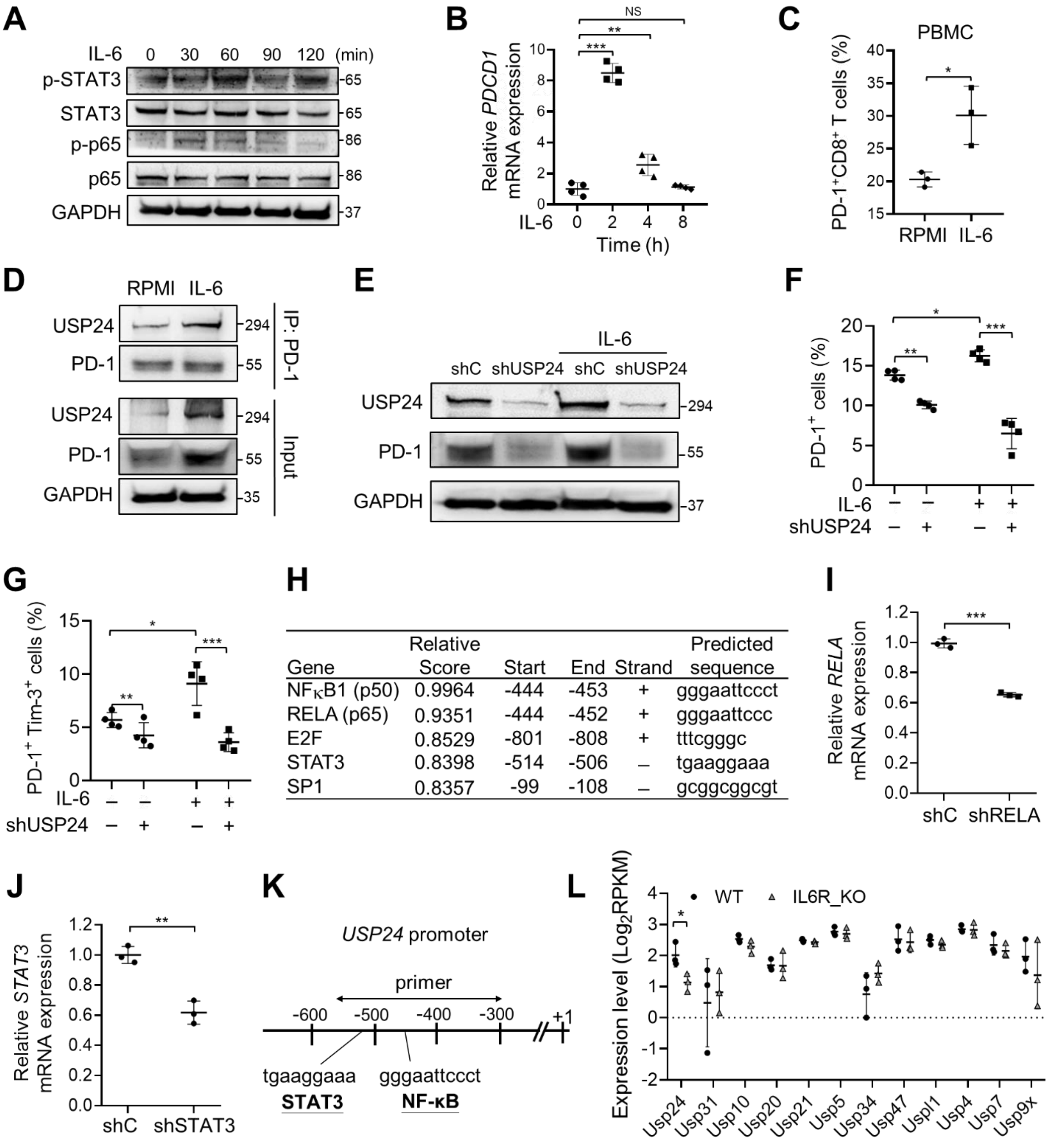

Figure S4. IL-6 increases the transcriptional level of PD-1 and USP24 and promotes deubiquitination of PD-1. (A) Immunoblots of p-p65 (NF-κB) and p-STAT3 in Jurkat T cells with IL-6 (50 *n*g/ml) stimulation for the indicated times**. (B)** RT-qPCR for detecting *PDCD1* mRNA expression in Jurkat T cells with IL-6 stimulation at the indicated times**. (C)** PBMCs were treated with IL-6 for 6 h followed by PD-1$^+$CD8$^+$ T cells detection. **(D)** IP of PD-1 in Jurkat T cells stimulated with IL-6 for 8 h. **(E and F)** PD-1 protein expression (E) and surface levels (F) in shC and shUSP24 Jurkat T cells with or without IL-6 stimulation for 6 h. **(G)** PD-1$^+$Tim-3$^+$ populations in shC and shUSP24 Jurkat T cells with IL-6 treatment for 16 h. **(H)** Predicted binding score and sequence of the indicated transcription factor on *USP24* promoter using JASPAR software. **(I and J)** RT-qPCR to examine knockdown efficiency of shRELA and shSTAT3 in Jurkat T cells. **(K)** Diagrams showing the predicted binding sequence of STAT3 and NF-κB on the USP24 promoter. **(L)** Gene expression of Usp24 in CD8$^+$ TILs from mice with knockout of IL-6 receptor (IL6R_KO). Data were from the GSE199047 database. Data were mean ± s.e.m. *, $P < 0.05$; **, $P < 0.01$; ***, $P < 0.001$ (one-way ANOVA).

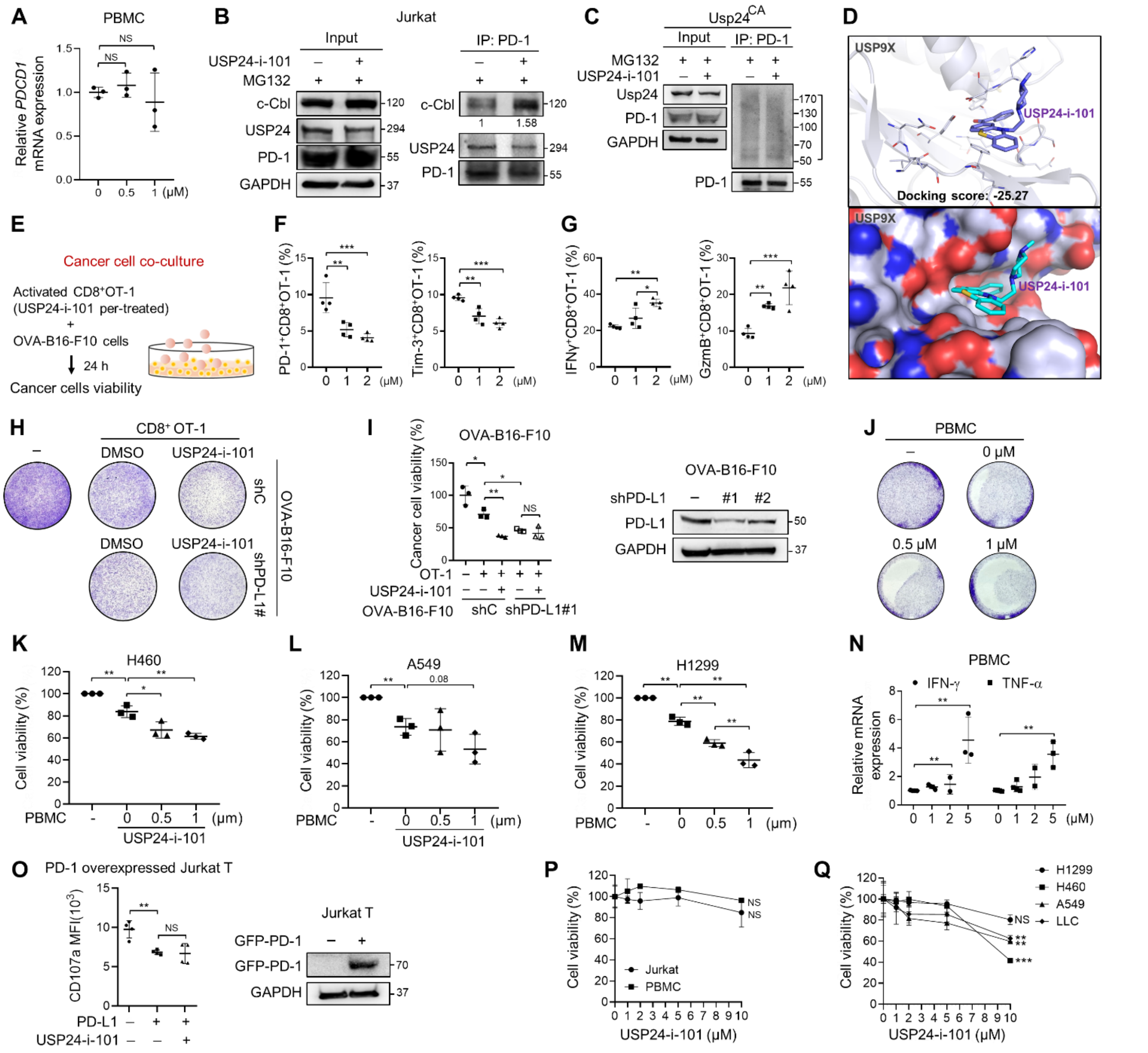

Figure S5. USP24-i-101 suppresses T cell dysfunction without T cell cytotoxicity. (A) RT-qPCR to measure *PDCD1* mRNA expression in anti-CD3/CD28 antibodies stimulated PBMCs with different doses of USP24-i-101 treatment for 48 h. **(B)** IP of PD-1 in anti-CD3/CD28 antibodies stimulated Jurkat T cells with USP24-i-101 and MG132 treatment for 24 h, and then subjected to detect the interaction between PD-1, c-Cbl, and USP24.**(C)** PD-1 ubiquitination levels in *Usp24*^{C1695A} mice derived splenic CD8^{+} T cells with USP24-i-101 treatment for 48 h. **(D)** Docking pose of the compound in USP9X. **(E)** The schematic diagram of *in vitro* T cell-mediated killing assays. **(F and G)** Populations of PD-1^{+}, Tim-3^{+} (F), IFN-γ^{+}, and GzmB^{+} CD8^{+}OT-1^{+} T cells (G) after co-culturing with OVA-B16F10 cancer cells. (**H and I**) (H) CD8^{+}OT-1^{+} T cells were pre-treated with USP24-i-101 (1 μm) for 48 h and then co-cultured with shC or shPD-L1 OVA-B16F10 cells (E:T ratio = 5:1) for an additional 24 h, followed by crystal violet staining to measure cancer cell viability. (I) Quantification using image J software (*left*). Immunoblotting shows the knockdown efficiency of shPD-L1 harboring OVA-B16-F10 cells (*right*). **(J to M)** PBMCs pre-treated with indicated doses of USP24-i-101 for 48 h were co-cultured with lung cancer cells H460, A549, and H1299 cells (E:T ratio = 5:1) for an additional 24 h. **(N)** RT-qPCR for detecting *IFN-γ* and *TNF-α* expression in PBMCs after co-culturing with cancer cells. **(O)** Jurkat T cells ectopically expressed with GFP-PD-1 were treated with PD-L1 Fc protein (2 μg/ml) and USP24-i-101 (1 μm) for 24 h. T cell functional marker CD107a was assessed by flow cytometry analysis (*left*). Immunoblots show the transfection efficiency in Jurkat T cells ectopically expressing GFP-PD-1 (*right*). **(P and Q)** CCK-8 assay was conducted to determine the cytotoxic effect of USP24-i-101 treatment for 48 h in Jurkat T cells and PBMCs (K) or various lung cancer cells (L). Data were mean ± s.e.m. (n=3). *, $P < 0.05$; **, $P < 0.01$; ***, $P < 0.001$ (one-way ANOVA).

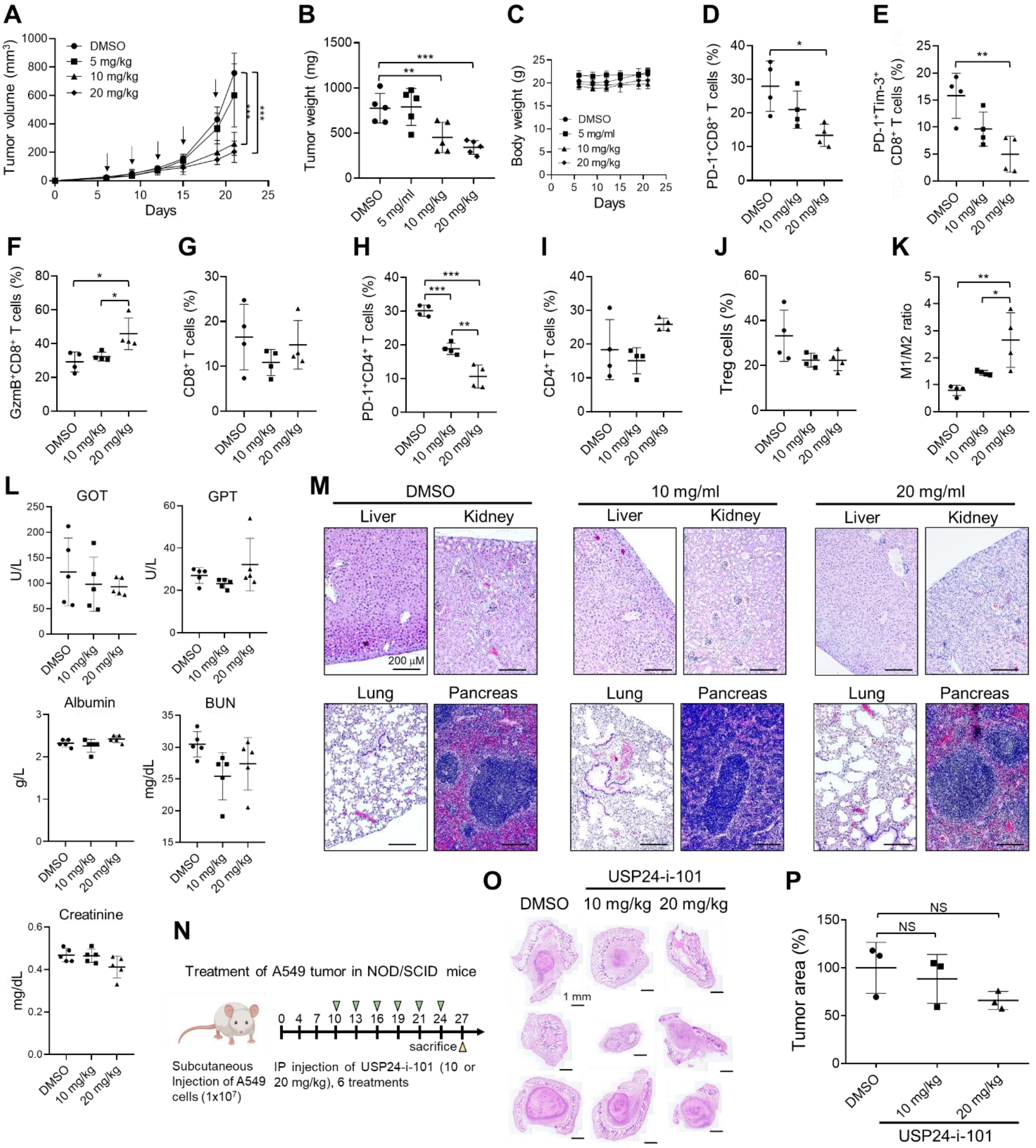

Figure S6. USP24-i-101 increases T cell anti-tumor immunity to suppress tumor growth *in vivo*. The allograft model was established using Lewis lung carcinoma (LLC) and treated with DMSO, 5 mg/kg, 10 mg/kg, or 20 mg/kg of USP24-i-101 twice every week by intraperitoneal injection (5 mice per group). **(A to C)** The changes in tumor volume (A), tumor weight (B), and body weight (C) during the *in vivo* experimental period. **(D to I)** Populations of tumor-infiltrating PD-1^+ (D), PD-1^+Tim-3^+ (E), GzmB$^+$ (F), and total CD8^+ T cells (G), as well as PD-1^+ (H) and total CD4^+ T cells (I) were assessed in the endpoint tumors by flow cytometry. **(J and K)** Tregs (J) and M1/M2 ration of tumor-associated macrophages (CD86$^+$/CD206$^+$) (K) were analyzed by flow cytometry. **(L and M)** Blood biochemistry analysis (L) and H&E staining (M) of tissue section from major organs in endpoint LLC-bearing mice with DMSO or indicated dose of USP24-i-101 treatment. **(N)** NOD-SCID mice implanted with A549 lung cancer cells were injected with the indicated dose of USP24-i-101. **(O, P)** Represented H&E staining of tumor images using TissueFAXs microscope (O) and tumor volume quantification at the end of the experiment (P). Data were mean ± s.e.m. *, $P < 0.05$; **, $P < 0.01$; ***, $P < 0.001$ (one-way ANOVA).

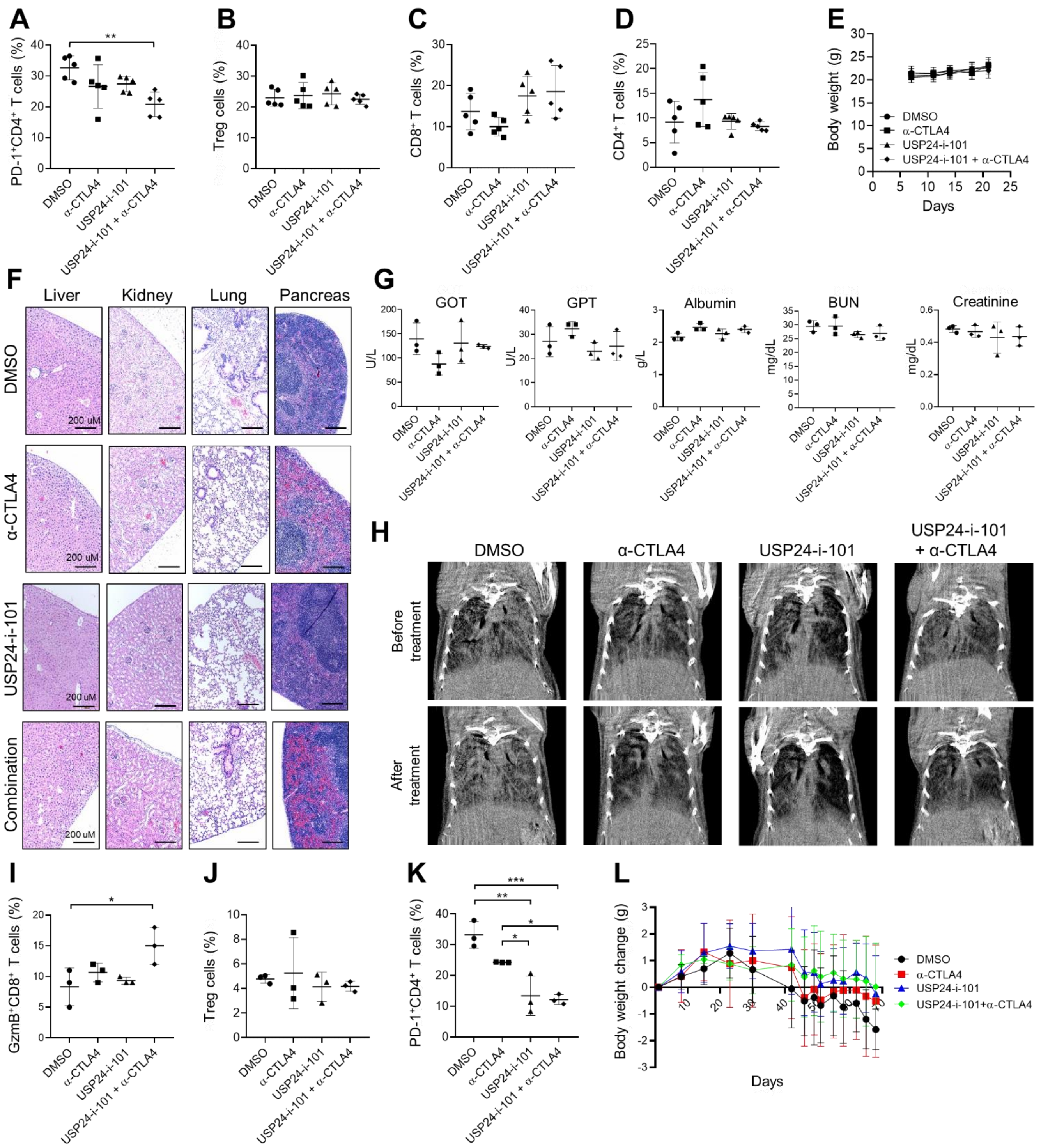

Figure S7. Pathological and flow cytometric examinations in mice with USP24-i-101 and α-CTLA4 combination therapy. (A to D) Percentage of tumor-infiltrating PD-1$^+$ CD4$^+$ T cells (A), Tregs (B), total CD8$^+$ T cells (C), and total CD4$^+$ T cells (D) were measured by flow cytometry. **(E)** The body weight of mice with LLC-allograft was assessed during the experimental period. **(F to G)** H&E staining of tissue sections from major organs (F) and blood biochemistry analysis (G) were performed at the endpoint. **(H)** Representative images of lung tumors by micro-CT. Data were taken before and after indicted treatment**. (I to L)** The percentage of GzmB+ CD8+ T cells (I), Tregs (J), and PD-1$^+$CD4$^+$ T cells (K) in *Egfr*^{L858R}-driven lung tumors and body weight during the experiment (L) were assessed. Data were mean ± s.e.m. *, $P < 0.05$; **, $P < 0.01$; ***, $P < 0.001$ (one-way ANOVA).

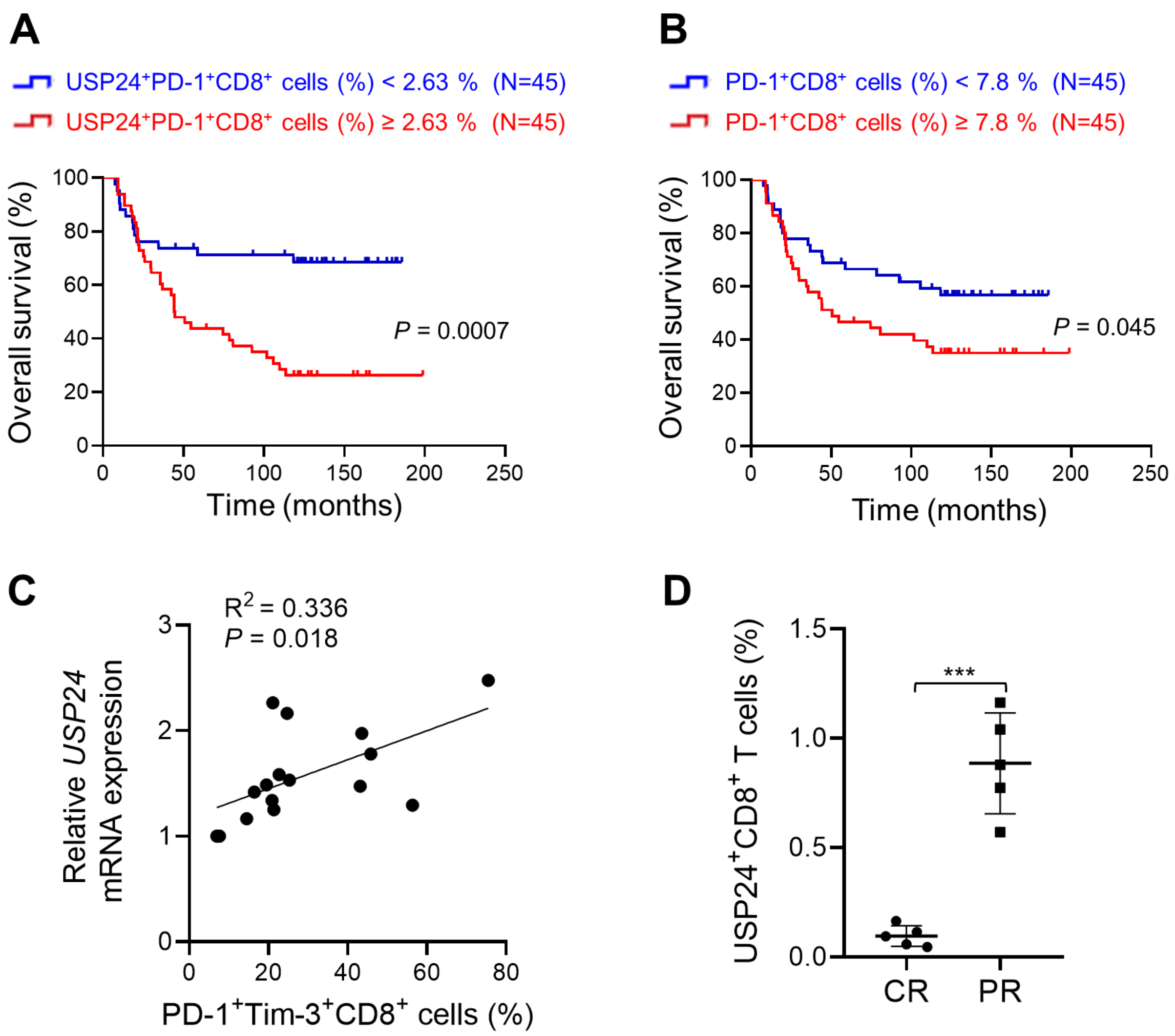

Figure S8. Elevated USP24 expression in T cells is associated with impaired T cell anti-tumor activity. (A and B) Overall survival curve in lung cancer patients with high or low infiltrating USP24$^+$PD-1$^+$CD8$^+$ T cell (A) or PD-1$^+$CD8$^+$ T cells (B). **(C)** Scatter plot showing the correlation between the levels of *USP24* mRNA expression and the percentage of PD-1$^+$Tim-3$^+$CD8$^+$ cells in PBMCs derived from lung cancer patients (n=16). Pearson correlation coefficient, R square, and P-value are shown. **(D)** Quantitative results of USP24$^+$ CD8$^+$ T cells of Fig. 7 H and I. Data are mean ± s.e.m. *, $P < 0.05$; **, $P < 0.01$; ***, $P < 0.001$ (Student's t-test).

Table S1. Alteration of USP24^{+}/PD-1^{+}/CD8^{+} cells in relation to clinicopathological parameters in 90 lung cancer patients' tumor specimens.

Clinical Parameters		Total Patients	USP24^{+}/PD-1^{+}/CD8^{+} (%) Protein Expression		*P*-value [a]
		**90**	**N=45 Low expression [b]**	**N=45 High expression [b]**	
**Age**	<65	52	19 (36.5 %)	33 (63.5 %)	**0.021**
	≥65	38	23 (60.5 %)	15 (39.5 %)	
**Sex**	Male	41	21 (51.2 %)	20 (48.3 %)	0.281
	Female	49	21 (42.8 %)	28 (57.2 %)	
**Tumor type [c]**	ADC	73	27 (36.0 %)	46 (64.0 %)	**<0.001**
	SCC	17	15 (88.2 %)	2 (11.8 %)	
**Tumor stage**	I-II	57	41 (71.9 %)	16 (28.1 %)	**<0.001**
	III-IV	33	1 (3.0 %)	32 (97 %)	
**T stage [d]**	T1-T2	74	40 (54.0 %)	34 (46.0 %)	**0.004**
	T3-T4	15	2 (13.3 %)	13 (86.7 %)	
**N stage [d]**	≤N1	47	37 (78.7 %)	10 (21.3 %)	**<0.001**
	>N1	43	5 (11.6 %)	38 (88.4 %)	
**M stage [d]**	M0	83	41 (49.3 %)	42 (50.7 %)	0.079
	≥M1	7	1 (14.2 %)	6 (85.8 %)	
**Differentiation grade**	Well	16	6 (37.5 %)	10 (62.5 %	0.425
	Moderate	61	28 (45.9 %)	33 (54.1 %)	
	Poor	13	8 (61.5 %)	5 (38.5 %)	

[a]. The data were analyzed by Pearson $\chi 2$ test. P values are shown as superscripts.

[b]. Patient with USP24^{+}/PD-1^{+}/CD8^{+} cells (%) ≥ 2.63 % was defined as high expression.

[c]. ADC, adenocarcinoma; SCC, squamous cell carcinoma; Other, adenosquamous carcinoma.

[d]. T stage: primary tumor; N stage: lymph node metastasis; M stage: distant metastasis.

Table S2. Antibodies and their reaction conditions used in the current study.

Target	KD	Raised in	Application	Dilution	Source	Catalog no.
USP24	294	Rabbit	Western blot	1:1000	Proteintech	13126-1-AP
	--[a]		Immunofluorescence	1:2000		
PD-1	55	Rabbit	Western blot	1:1000	Cell Signaling	86163S
	--[a]	Rabbit	Immunofluorescence	1:1000		
		Mouse	Flow cytometry (BB515)	1:200	BD Bioscience	565936
c-Cbl	120	Rabbit	Western blot	1:200	Santa Cruz	sc-170
GFP-tag	--[a]	Rabbit	Western blot	1:1000	GeneTex	GTX113617
HA-tag	--[a]	Rabbit	Western blot	1:1000	Invitrogen	PA1-985
Ubiquitin	--[a]	Mouse	Western blot	1:200	Santa Cruz	sc-8017
GAPDH	37	Mouse	Western blot	1:1000	Santa Cruz	sc-32233
Calnexin	90	Rabbit	Western blot	1:1000	GeneTex	GTX109669
Na^+/K^+ATPase	113	Rabbit	Western blot	1:100000	Abcam	ab76020
Rab11b	24	Rabbit	Western blot	1:1000	GeneTex	GTX119095
p65	65	Rabbit	Western blot	1:1000	Cell Signaling	8242S
p-p65	65	Rabbit	Western blot	1:1000	Cell Signaling	3033S
STAT3	79	Rabbit	Western blot	1:1000	Cell Signaling	9139S
p-STAT3	79	Rabbit	Western blot	1:1000	Cell Signaling	9145S
BAX	20	Rabbit	Western blot	1:1000	Cell Signaling	#2774

CD8	--[a]	Rabbit	Immunofluorescence	1:1000	Abcam	ab108343
		Mouse	Flow cytometry (APC)	1:200	BD Bioscience	561952
Tim-3	--[a]	Mouse	Flow cytometry (BV421)	1:200	BD Bioscience	565562
Lag-3	--[a]	Rabbit	Immunofluorescence	1:1000	Abcam	ab237720
IFN-γ	--[a]	Mouse	Flow cytometry (BV421)	1:200	BD Bioscience	562988
CD107a	--[a]	Mouse	Flow cytometry (PE-Cy7)	1:200	BD Bioscience	561348
Ki-67	--[a]	Mouse	Flow cytometry (BV510)	1:200	BD Bioscience	563462
Mouse CD8	--[a]	Rabbit	Immunofluorescence	1:1000	Abcam	ab217344
		Rat	Flow cytometry (FITC)	1:200	BD Bioscience	553031
Mouse CD4	--[a]	Rat	Flow cytometry (FITC)	1:200	BD Bioscience	553047
Mouse PD-1	55	rabbit	Western blot	1:1000	Cell Signaling	84651T
	--[a]		Immunofluorescence	1:1000		
		Rat	Flow cytometry (APC)	1:200	Biolegend	135210
Mouse PD-L1	50	Mouse	Western blot	1:1000	Proteintech	66248-1-Ig
Mouse Tim-3	--[a]	Mouse	Flow cytometry (BV421)	1:200	BD Bioscience	747626
Mouse IFN-γ	--[a]	Rat	Flow cytometry (Cy 5.5)	1:200	BD Bioscience	560660
Granzyme B	--[a]	Mouse	Flow cytometry (PE)	1:200	Biolegend	372207
Mouse CD25	--[a]	Mouse	Flow cytometry (PE)	1:200	BD Bioscience	562695
Mouse Foxp3	--[a]	Mouse	Flow cytometry (BV421)	1:200	BD Bioscience	562996
Mouse CD11b	--[a]	Mouse	Flow cytometry (BB515)	1:200	BD Bioscience	563055
Mouse CD86	--[a]	Mouse	Flow cytometry (BB605)	1:200	BD Bioscience	563055

Mouse CD206	--[a]	Mouse	Flow cytometry (APC)	1:200	BD Bioscience	565250
Mouse IL-1β	--[a]	Mouse	Immunohistochemistry	1:250	Cell Signaling	#12242
Mouse TNF-α	--[a]	Rabbit	Immunohistochemistry	1:250	Cell Signaling	#11948
DAPI[b]	--[a]	--[c]	Immunofluorescence	--[b]	Genetex	GTX30920
Opal 520 Reagent	--[a]	--[c]	Florescent immunohistochemistry	1:100	Akoya Biosciences	FP1013
Opal 570 Reagent	--[a]	--[c]	Florescent immunohistochemistry	1:100	Akoya Biosciences	FP1014
Opal 620 Reagent	--[a]	--[c]	Florescent immunohistochemistry	1:100	Akoya Biosciences	FP1495A
Opal 670 Reagent	--[a]	--[c]	Florescent immunohistochemistry	1:100	Akoya Biosciences	FP1117

[a] Molecular weight is not applicable to this antibody in such an application.

[b] DAPI is a commercial product for nuclear staining.

[c] Species is not applicable to this antibody in such an application.

Table S3. The plasmids and their characteristics used in the current study.

Plasmid	Target	Insert (bp)	Function	Source
pEGFP-C3	None	–[a]	Vector control	Homemade
pEGFP-USP24-WT	USP24-WT	7862	Overexpression	Homemade
pEGFP-USP24-ΔC	USP24-ΔC	6150	Overexpression	Homemade
pEGFP-USP24-ΔN	USP24-ΔN	1712	Overexpression	Homemade
pEGFP-USP24-C1698A	USP24-C1698A	7862	Overexpression	Homemade
pcDNA3.1-GFP-PD-1 WT	WT-PD-1	866	Overexpression	Sino Biological Inc
pcDNA3.1-GFP-PD-1-ΔECD	PD-1-ΔECD	351	Overexpression	Homemade
pcDNA3.1-GFP-PD-1-ΔTM	PD-1-ΔTM	804	Overexpression	Homemade
pcDNA3.1-GFP-PD-1-ΔICD	PD-1-ΔICD	573	Overexpression	Homemade
c-Cbl	c-Cbl	2720	Overexpression	Homemade
pCMV-HA	None	–[a]	Vector control	Homemade
pCMV-HA-ubiquitin	Total ubiquitin	220	Overexpression	Dr. Hui-Kuan Lin
pCMV-HA-K-48 ubiquitin	K-48 ubiquitin	220	Overexpression	Dr. Hui-Kuan Lin
pCMV-HA-K-63 ubiquitin	K-63 ubiquitin	220	Overexpression	Dr. Hui-Kuan Lin
pCMV6-XL4-STAT3-WT	STAT3	3384	Overexpression	Origene
pEGFP-NF-κB-p65	p65	2500	Overexpression	Dr. Yen-Ni Teng
pCDNA3-HA-SP1	SP1	2358	Overexpression	Homemade
pCMV-SPORT6-E2F1	E2F1	1314	Overexpression	Homemade

[a] The plasmid is used as a backbone vector and, therefore, has no inserted fragment.

Table S4. The primers used in the current study.

Gene	Primer	Sequences (5′→ 3′)	Application[a]	PCR size (bp)	Tm (°C)
Human *Actin* mRNA	Forward<br>Reverse	GGC GGC ACC ACC ATG TAC CCT<br>AGG GGC CGG ACT CGT CAT ACT	RT-qPCR	180	60
Human *USP24* mRNA	Forward<br>Reverse	CAG TTG TGC TCT CCT GTG GA<br>AGG GAT TTC TCC TGC TCC AT	RT-qPCR	236	60
Human *PCCD1* mRNA	Forward<br>Reverse	GGC CAG GAT GGT TCT TAG ACT<br>GGT ACC AGT TTA GCA CGA AGC T	RT-qPCR	147	60
Human *IFN-r* mRNA	Forward<br>Reverse	GTC CAA CGC AAA GCA ATA CAT G<br>CTC GAA ACA GCA TCT GAC TCC TT	RT-qPCR	100	60
Human *TNF-a* mRNA	Forward<br>Reverse	CCC AGG GAC CTC TCT CTA ATC A<br>AGC TGC CCC TCA GCT TGA G	RT-qPCR	116	60
Human *STAT3* mRNA	Forward<br>Reverse	CTT TGA GAC CGA GGT GTA TCA CC<br>GGT CAG CAT GTT GTA CCA CAG G	RT-qPCR	133	60
Human *RELA* mRNA	Forward<br>Reverse	TGA ACC GAA ACT CTG CAG CTG<br>CAT CAG CTT GCG AAA AGG AGC C	RT-qPCR	134	60
Mouse *Actin* mRNA	Forward<br>Reverse	GGC TCT TTT CCA GCC TTC CT<br>GTC TTT ACG GAT GTC AAC GTC ACA	RT-qPCR	100	60
Mouse *PDCD1* mRNA	Forward<br>Reverse	CGT CCC TCA GTC AAG AGG AG<br>GTC CCT AGA AGT GCC CAA CA	RT-qPCR	243	60
Mouse *Usp24* mRNA	Forward<br>Reverse	GAA CTG GGC AGA GGT GTT TG<br>TGG AGT TTG GCT TGG ATT GC	RT-qPCR	147	60
USP24 promoter-ChIP	Forward<br>Reverse	GCA TTT CAG GCC GGG GTG<br>CTG AAC GTC GCG GAA ATC C	RT-qPCR	198	60

[a] RT-qPCR, quantitative reverse-transcriptase polymerase chain reaction.
[b] Information is not applicable.

Table S5. Clinical information for human lung cancer tissue microarray. Tumor stages are described as tumor (T), lymph node (N), or metastasis (M).

Patient number	Sex	Age	Tumor type	Tumor stage
NSCLC-1	Female	54	ADC	T1N0M0
NSCLC-2	Female	66	ADC	T1N0M0
NSCLC-3	Male	44	ADC	T1N0M0
NSCLC-4	Female	61	ADC	T2N0M0
NSCLC-5	Female	65	ADC	T2N0M0
NSCLC-6	Male	77	ADC	T2N0M0
NSCLC-7	Male	69	ADC	T2N0M0
NSCLC-8	Female	66	ADC	T2N0M0
NSCLC-9	Female	62	ADC	T2N0M0
NSCLC-10	Male	56	ADC	T2N0M0
NSCLC-11	Female	71	ADC	T2N0M0
NSCLC-12	Female	58	ADC	T2N0M0
NSCLC-13	Female	69	ADC	T2N0M0
NSCLC-14	Male	73	ADC	T2N0M0
NSCLC-15	Female	70	ADC	T2N0M0
NSCLC-16	Female	56	ADC	T1N0M0
NSCLC-17	Male	76	ADC	T1N0M0
NSCLC-18	Female	54	ADC	T1N0M0
NSCLC-19	Male	67	ADC	T1N0M0
NSCLC-20	Female	70	ADC	T1N0M0
NSCLC-21	Female	52	ADC	T1N0M0
NSCLC-22	Female	59	ADC	T1N0M0
NSCLC-23	Female	56	ADC	T1N0M0
NSCLC-24	Female	60	SCC	T2N0M0
NSCLC-25	Male	80	SCC	T2N0M0
NSCLC-26	Male	57	SCC	T1N0M0
NSCLC-27	Male	76	SCC	T2N0M0
NSCLC-28	Male	77	SCC	T3N0M0

NSCLC-29	Male	69	SCC	T2N0M0
NSCLC-30	Male	71	SCC	T2N0M0
NSCLC-31	Female	61	SCC	T2N0M0
NSCLC-32	Male	60	SCC	T1N0M0
NSCLC-33	Male	75	SCC	T1N0M0
NSCLC-34	Male	83	SCC	T1N0M0
NSCLC-35	Female	74	ADC	T1N0M0
NSCLC-36	Male	55	ADC	T1N0M0
NSCLC-37	Male	71	ADC	T2N0M0
NSCLC-38	Male	59	ADC	T2N0M0
NSCLC-39	Male	65	ADC	T2N0M0
NSCLC-40	Female	69	ADC	T2N0M0
NSCLC-41	Female	61	SCC	T1N0M0
NSCLC-42	Female	73	SCC	T1N0M0
NSCLC-43	Female	64	SCC	T1N0M0
NSCLC-44	Female	55	SCC	T1N0M0
NSCLC-45	Male	70	ADC	T2N0M0
NSCLC-46	Male	62	ADC	T1N0M0
NSCLC-47	Female	52	ADC	T2N1M0
NSCLC-48	Female	53	ADC	T2N1M0
NSCLC-49	Male	59	ADC	T1N1M0
NSCLC-50	Male	56	ADC	T3N0M0
NSCLC-51	Female	62	ADC	T1N1M0
NSCLC-52	Male	64	ADC	T2N2M0
NSCLC-53	Male	61	ADC	T1N1M0
NSCLC-54	Male	67	ADC	T1N1M0
NSCLC-55	Male	78	ADC	T3N0M0
NSCLC-56	Female	49	ADC	T2N1M0
NSCLC-57	Female	56	ADC	T1N1M0
NSCLC-58	Male	52	ADC	T3N0M0
NSCLC-59	Male	64	ADC	T1N1M1

NSCLC-60	Male	49	SCC	T2N2M1
NSCLC-61	Female	20	ADC	T2N2M1
NSCLC-62	Male	50	ADC	T4N2M1
NSCLC-63	Female	37	SCC	T2N2M1
NSCLC-64	Female	58	ADC	T3N1M0
NSCLC-65	Female	50	ADC	T4N2M0
NSCLC-66	Male	53	ADC	T2N2M0
NSCLC-67	Female	48	ADC	T2N2M1
NSCLC-68	Female	67	ADC	T2N2M0
NSCLC-69	Female	72	ADC	T2N2M0
NSCLC-70	Female	64	ADC	T1N2M0
NSCLC-71	Male	65	ADC	T2N2M1
NSCLC-72	Male	64	ADC	T1N2M0
NSCLC-73	Female	58	ADC	T4N2M0
NSCLC-74	Female	67	ADC	T2N2M0
NSCLC-75	Female	51	ADC	T4N1M0
NSCLC-76	Female	75	ADC	T4N1M0
NSCLC-77	Female	52	ADC	T3N2M0
NSCLC-78	Male	69	ADC	T4N2M0
NSCLC-79	Male	58	ADC	T2N2M0
NSCLC-80	Male	78	ADC	T4N2M0
NSCLC-81	Male	63	ADC	T3N2M0
NSCLC-82	Female	61	ADC	T2N2M0
NSCLC-83	Female	64	ADC	T2N2M0
NSCLC-84	Male	73	ADC	T2N2M0
NSCLC-85	Female	51	ADC	T1N3M0
NSCLC-86	Female	68	ADC	T1N2M0
NSCLC-87	Female	62	ADC	T2N2M0
NSCLC-88	Female	56	ADC	T3N2M0
NSCLC-89	Male	74	ADC	T2N2M0
NSCLC-90	Female	56	ADC	T2N1M0

Table S6. Clinical information for human lung cancer patients with immunotherapy.

Tumor stages are described as tumor (T), lymph node (N), or metastasis (M).

Patient number	Sex	Age	Tumor type	Tumor stage	Treatment	Response[a]
Imm-1	Male	60	ADC	T2N2M1	Durvalumab	R
Imm-2	Male	41	SSC	T4N2M1	Atezolizumab	R
Imm-3	Male	50	ADC	T4N3M1	Pembrolizumab	R
Imm-4	Male	52	ADC	T4N3M1	Pembrolizumab	R
Imm-5	Male	75	ADC	T2N3M1	Pembrolizumab	R
Imm-6	Male	62	ADC	T4N3M1	Cisplatin + Alimta + Pembrolizumab	R
Imm-7	Female	46	ADC	T4N3M1	Carboplatin + Alimta+ Nivolumab	R
Imm-8	Male	56	ADC	T4N3M1	Pembrolizumab	R
Imm-9	Male	69	ADC	T3N3M1	Pembrolizumab	R
Imm-10	Male	81	ADC	T4N3M1	Pembrolizumab	R
Imm-11	Male	75	ADC	T4N2M1	Pembrolizumab	NR
Imm-12	Male	67	SCC	T4N3M1	Pembrolizumab	NR
Imm-13	Female	69	ADC	T4N3M1	Atezolizumab	NR
Imm-14	Female	73	SCC	T3N0M0	Pembrolizumab	NR
Imm-15	Male	68	SCC	--[b]	Pembrolizumab	NR
Imm-16	Female	52	ADC	T4N3M1	Cisplatin + Alimta + Pembrolizumab	NR
Imm-17	Male	58	ADC	T4N3M1	Carboplatin + Alimta + Pembrolizumab	NR
Imm-18	Male	77	ADC	T1N0M0	Carboplatin + Alimta + Atezolizumab	NR
Imm-19	Male	71	SCC	T1N3M1	Carboplatin + Abraxane + Pembrolizumab	NR
Imm-20	Male	78	ADC	T3N2M1	Pembrolizumab	NR
Imm-21	Male	60	ADC	T3N3M1	Pembrolizumab	NR
Imm-22	Male	78	ADC	T4N3M1	Nivolumab	NR

[a] R, responder; NR, non-responder.

[b] Tumor staging information is not available to this patient.

Table S7. Clinical information for human lung cancer patients with neoadjuvant immunotherapy followed by surgical resection.

Patient number	Sex	Tumor type	Treatment	Tumor stage	Response[a]
NAI-1	Male	SCC	Durvalumab	2B	CR
NAI-2	Female	SCC	Pembrolizumab	3A	CR
NAI-3	Female	ADC	Pembrolizumab	3C	PR
NAI-4	Male	Poorly-differentiated carcinoma	Pembrolizumab	3B	PR
NAI-5	Male	ADC	Nivolumab	1B	CR
NAI-6	Male	ADC	Nivolumab	3A	PR
NAI-7	Male	ADC	Pembrolizumab	3A	CR
NAI-8	Male	ADC	Nivolumab	3B	PR
NAI-9	Male	SCC	Pembrolizumab	3B	PR
NAI-10	Male	SCC	Pembrolizumab	3B	CR

[a] CR, complete responder; PR, partial responder.

Data S1. Unprocessed blots for all Figures

Fig 2A

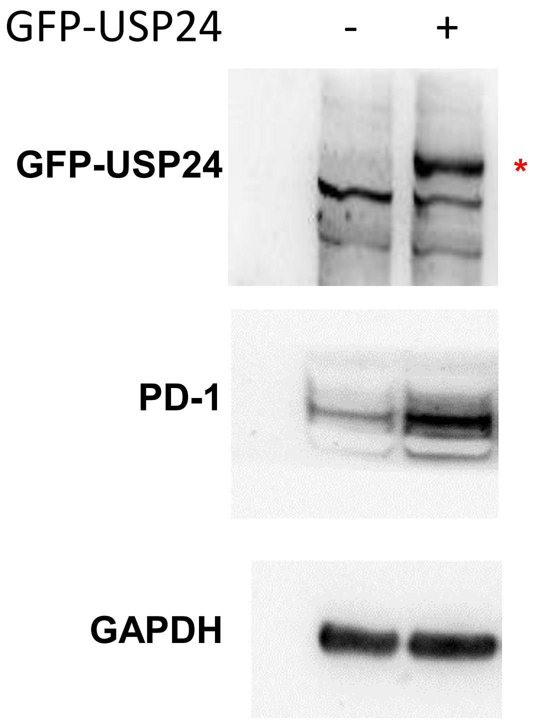

Fig 2B

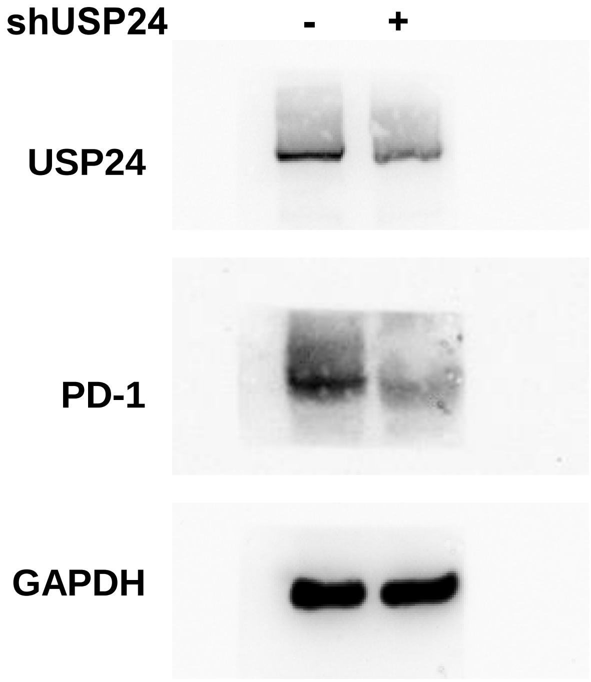

Fig 2D

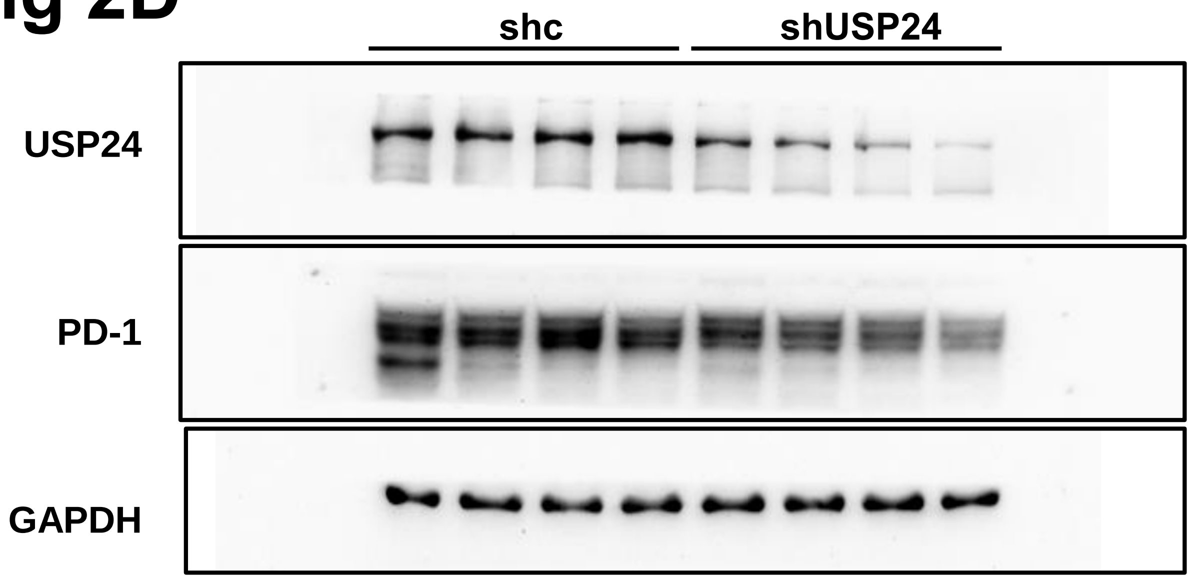
shc
shUSP24
USP24
PD-1
GAPDH

Fig 2E

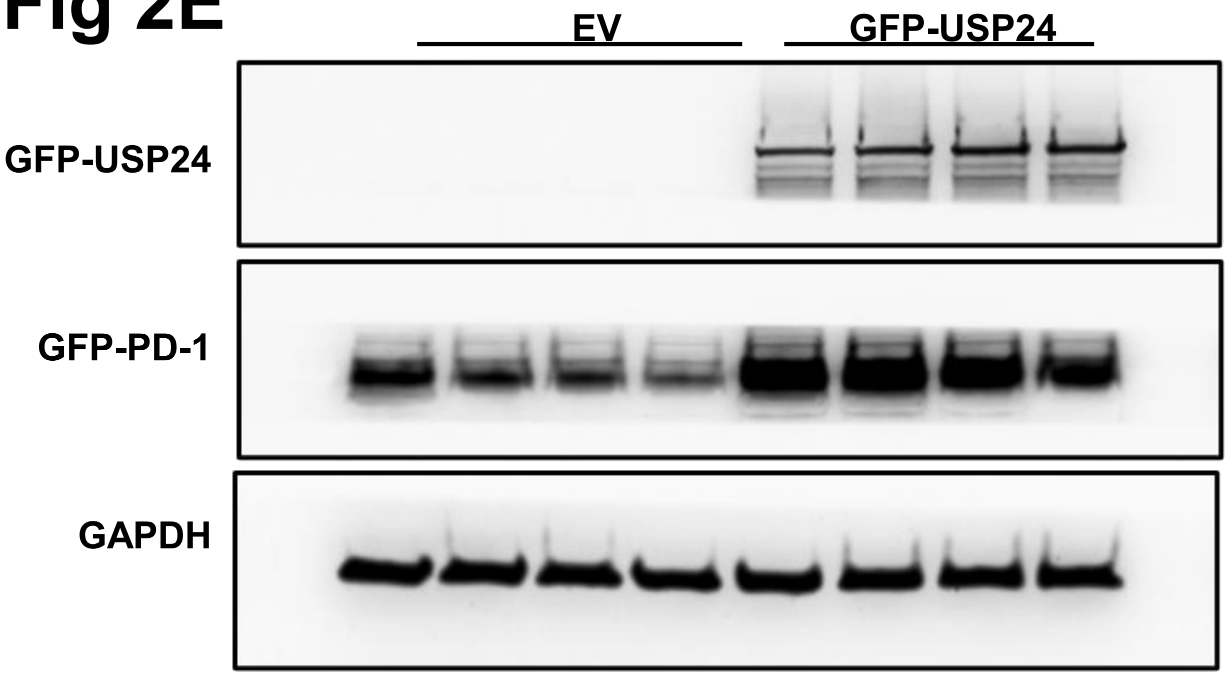
EV
GFP-USP24
GFP-USP24
GFP-PD-1
GAPDH

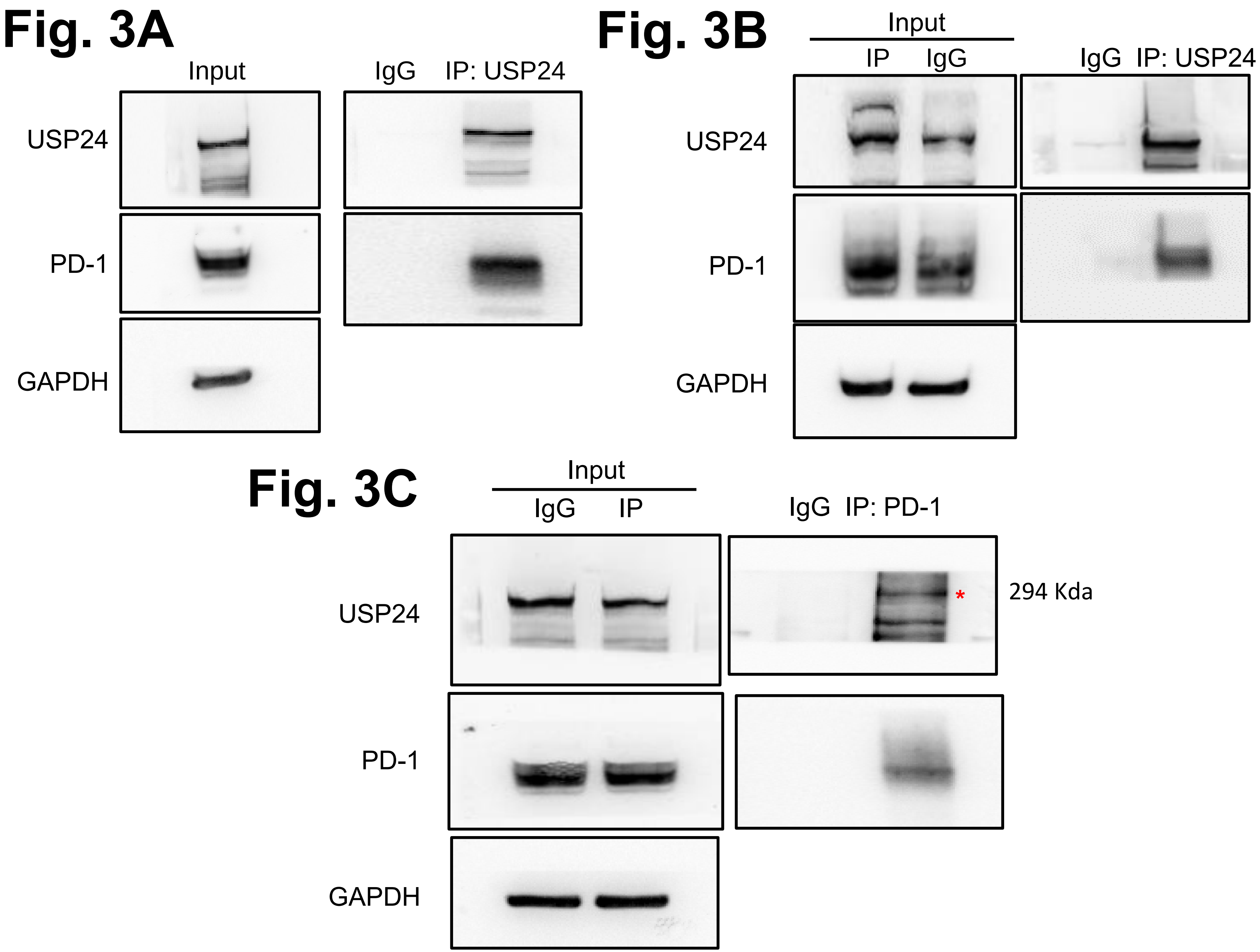
Fig. 3A
Input
IgG
IP: USP24
USP24
PD-1
GAPDH
Fig. 3B
Input
IP
IgG
IgG
IP: USP24
USP24
PD-1
GAPDH
Fig. 3C
Input
IgG
IP
IgG
IP: PD-1
USP24
294 Kda
PD-1
GAPDH

Fig. 3E

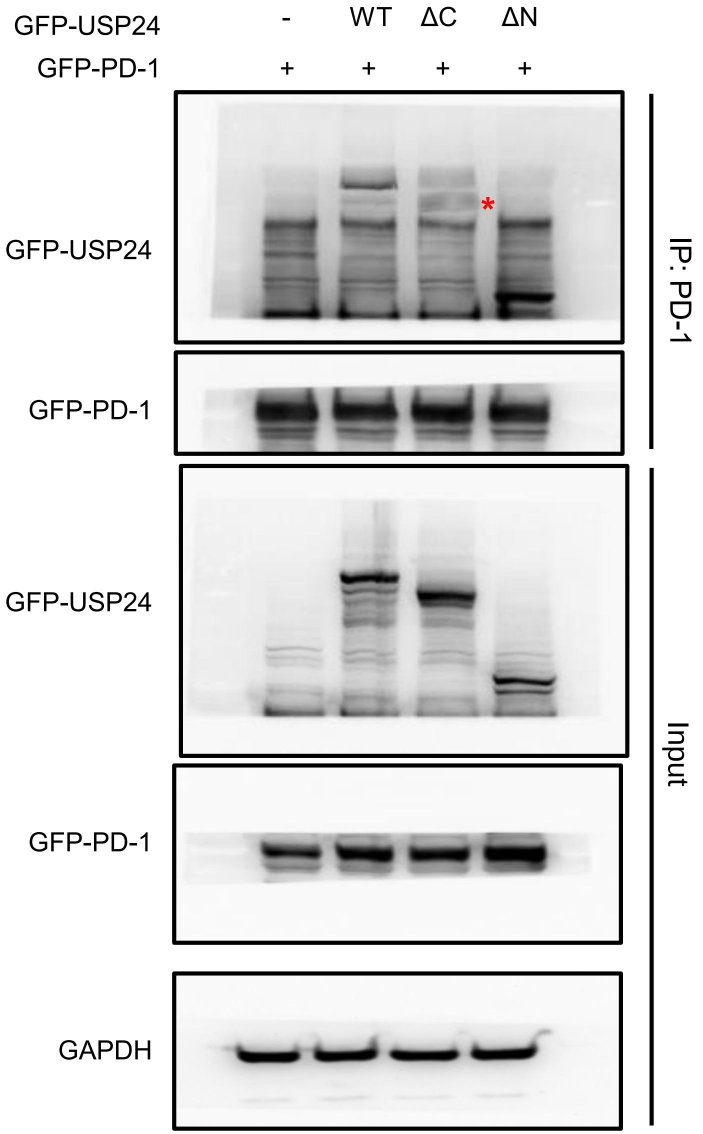

GFP-USP24
-
WT
ΔC
ΔN
GFP-PD-1
+
+
+
+
GFP-USP24
*
GFP-PD-1
IP: PD-1
GFP-USP24
GFP-PD-1
GAPDH
Input

Fig. 3G

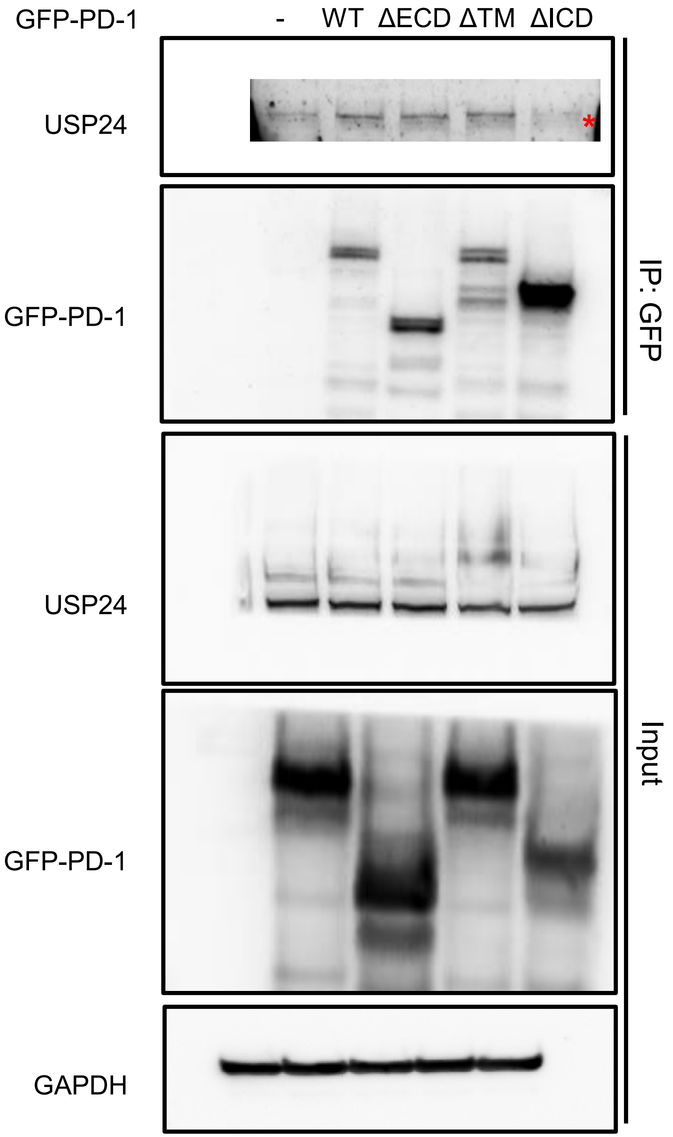

GFP-PD-1
-
WT
ΔECD
ΔTM
ΔICD
USP24
*
GFP-PD-1
IP: GFP
USP24
GFP-PD-1
GAPDH
Input

Fig. 3H

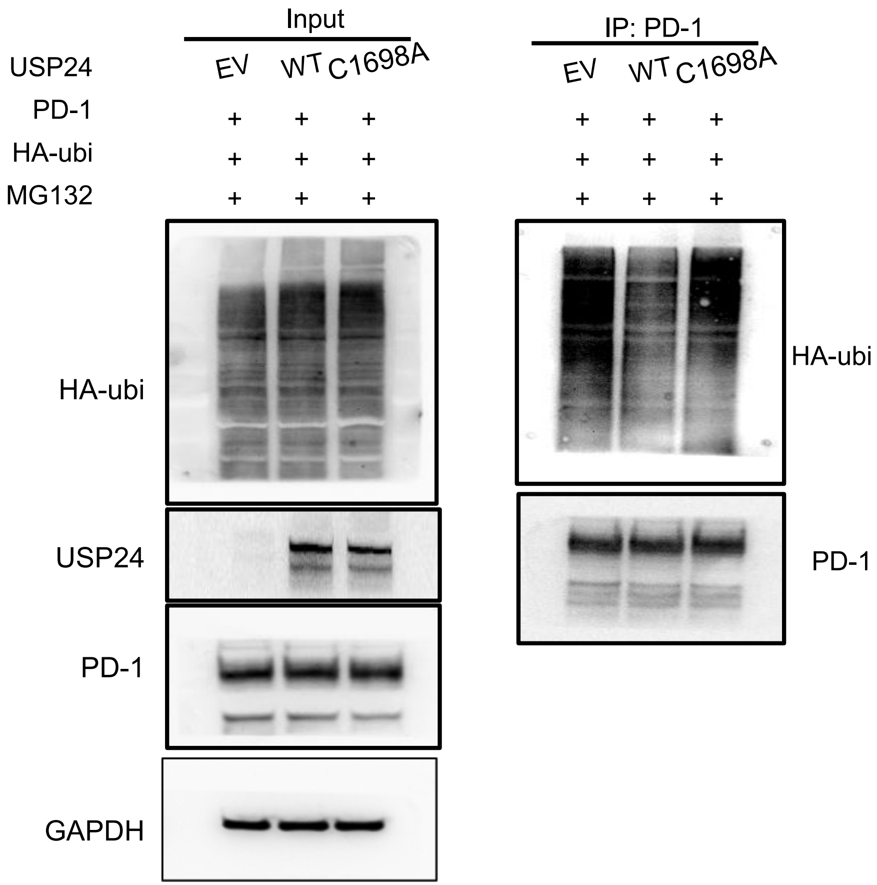
Input
IP: PD-1
USP24
EV
WT
C1698A
PD-1
HA-ubi
MG132
+
HA-ubi
USP24
PD-1
GAPDH

Fig. 3I

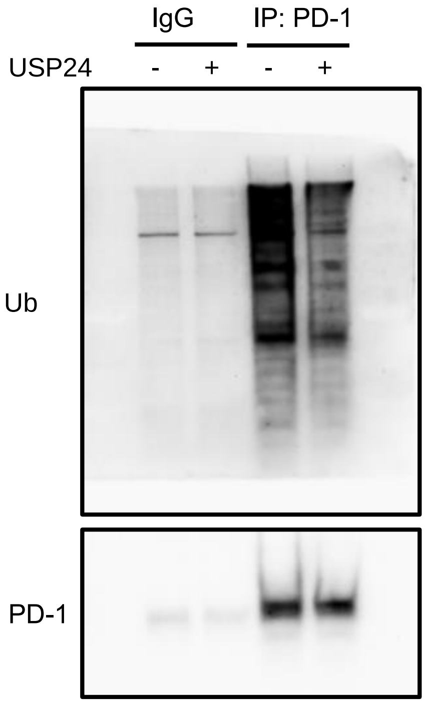
IgG
IP: PD-1
USP24
-
+
Ub
PD-1

Fig. 3J

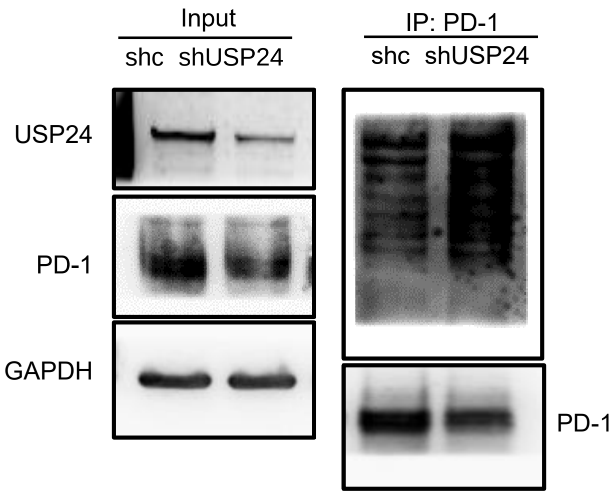

Fig. 3K

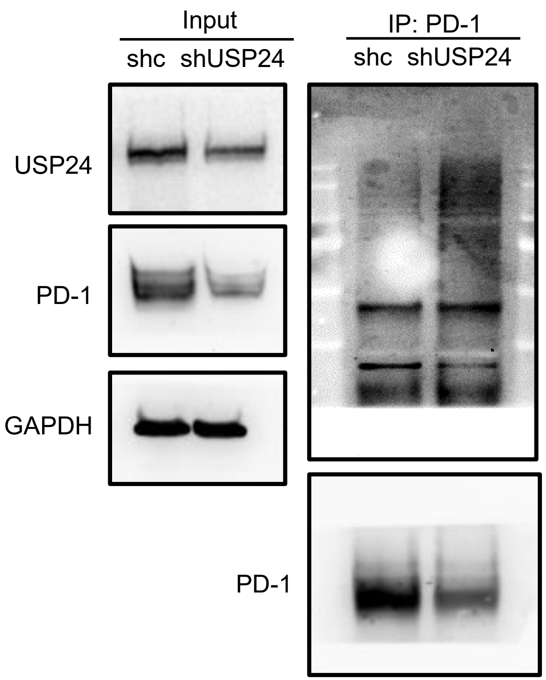

Fig. 3L

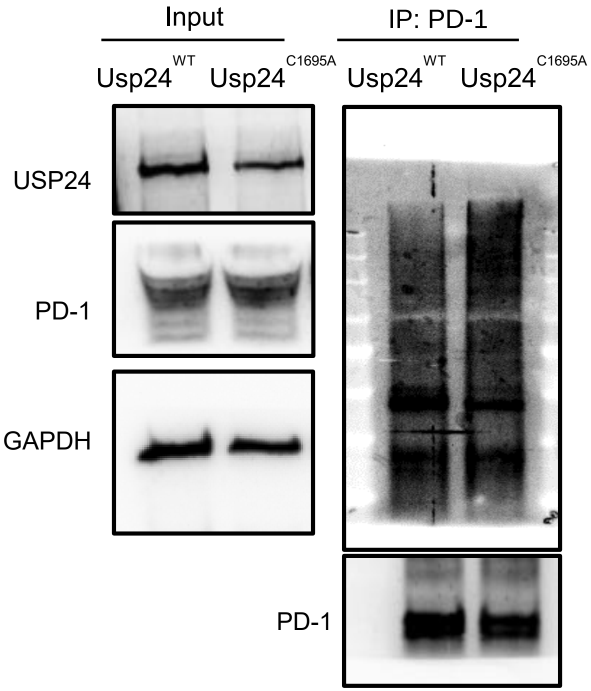

Fig. 4A

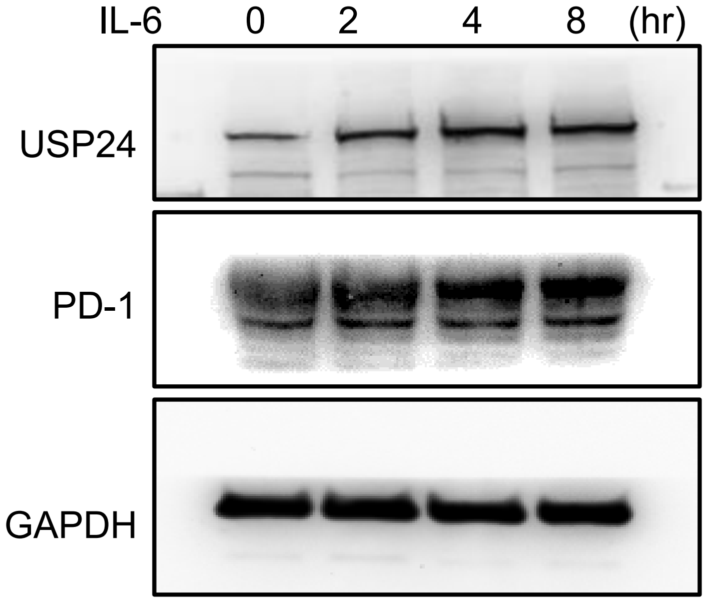

Fig. 4B

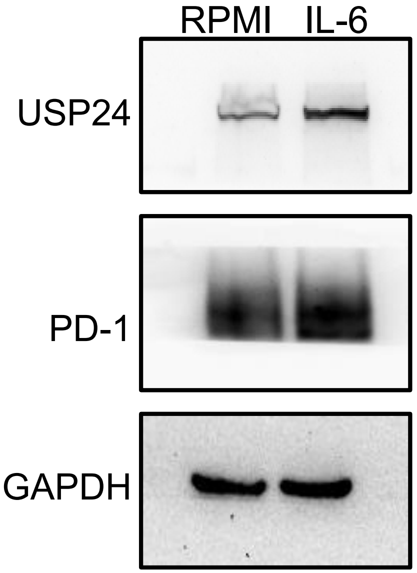

Fig. 4E

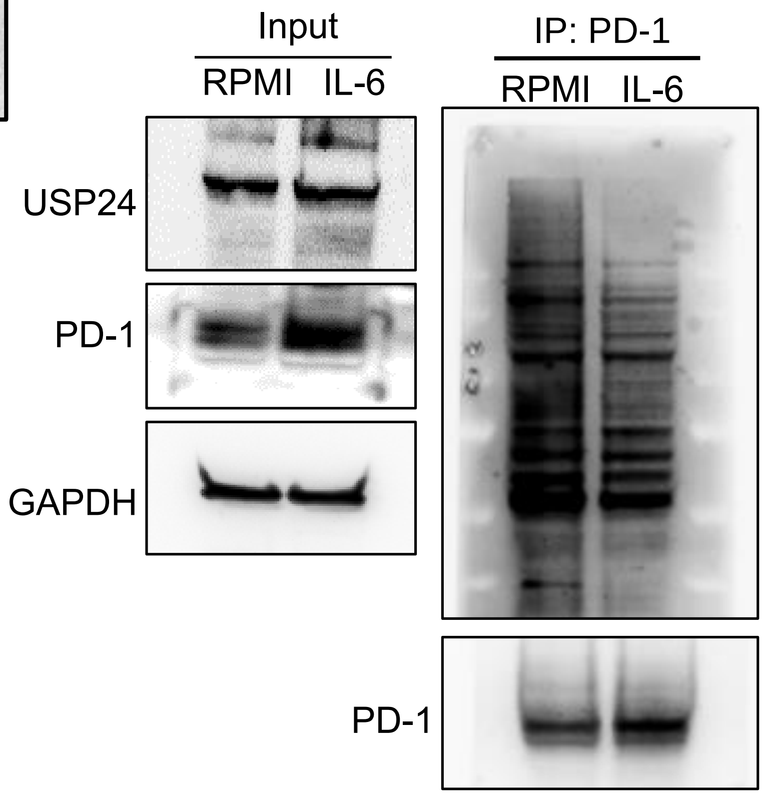

Fig. 4D

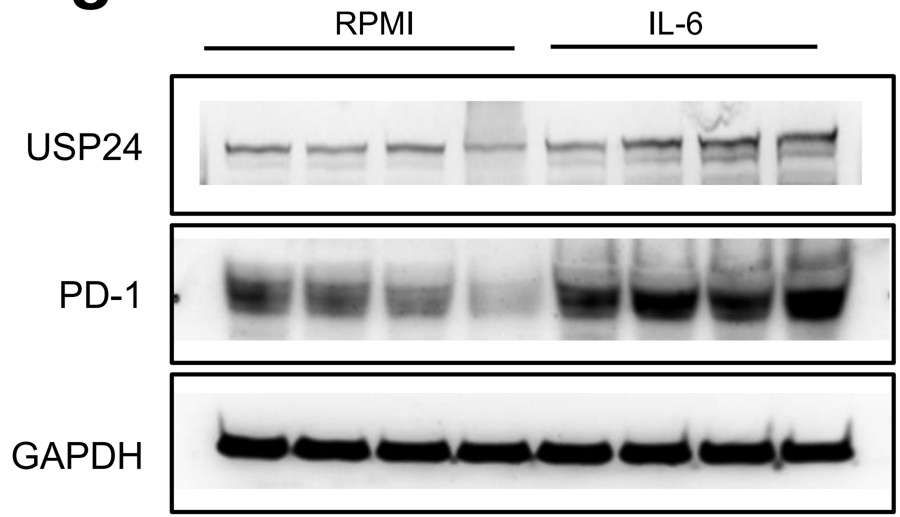

Fig. 5A

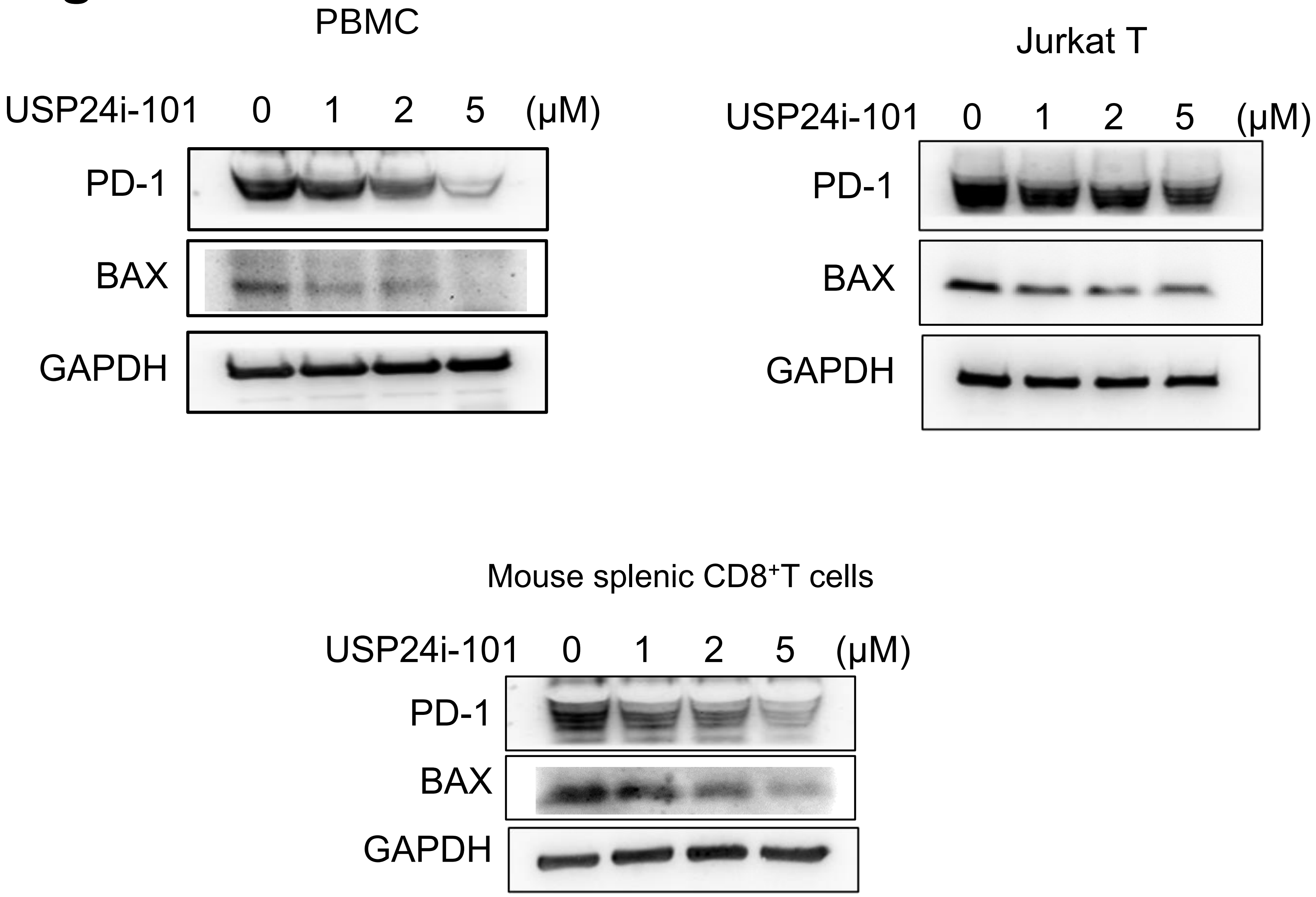
PBMC
USP24i-101 0 1 2 5 (μM)
PD-1
BAX
GAPDH
Jurkat T
USP24i-101 0 1 2 5 (μM)
PD-1
BAX
GAPDH
Mouse splenic CD8+T cells
USP24i-101 0 1 2 5 (μM)
PD-1
BAX
GAPDH

Fig. 5B

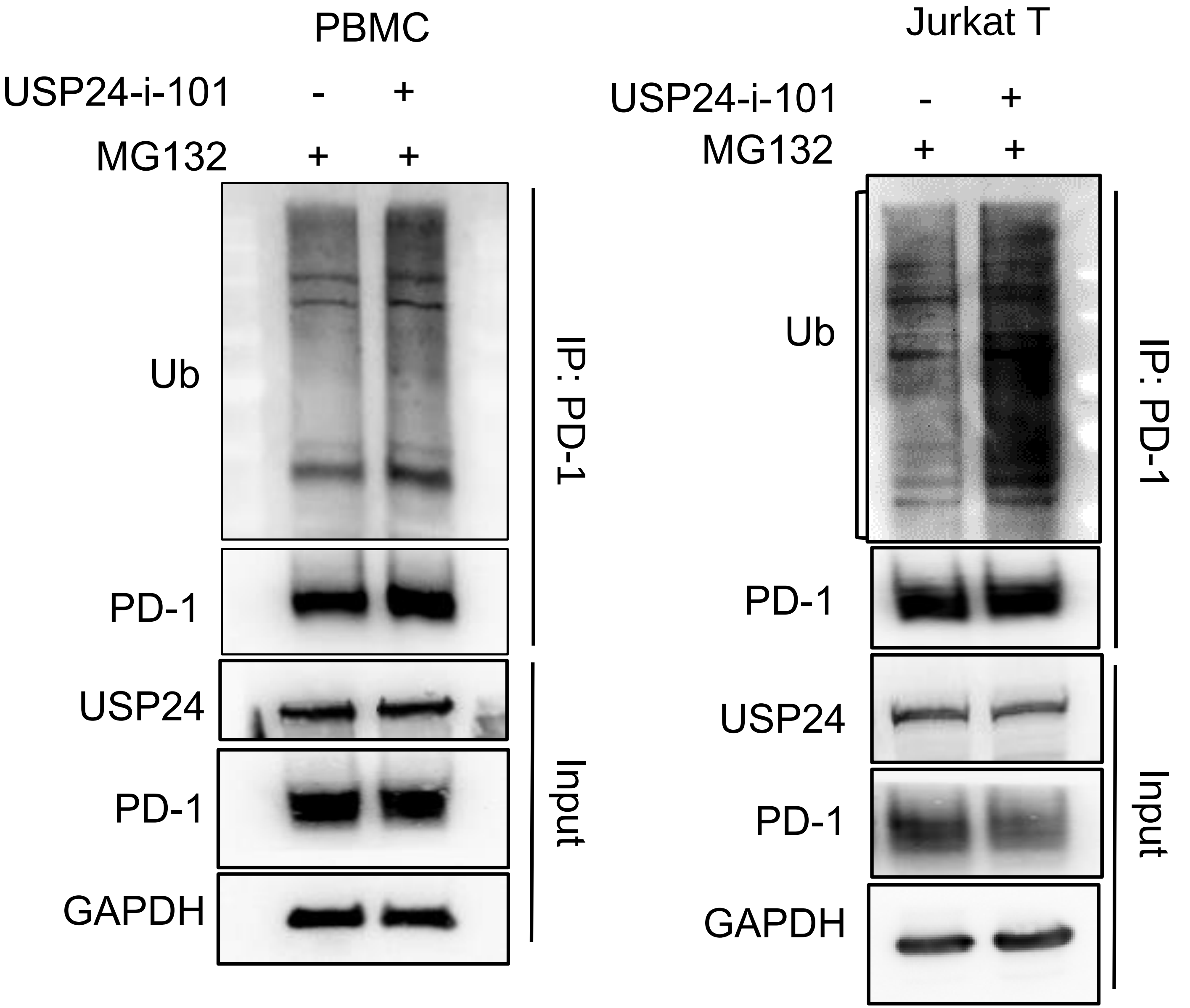

PBMC
USP24-i-101 - +
MG132 + +
Ub
PD-1
USP24
PD-1
GAPDH
IP: PD-1
Input
Jurkat T
USP24-i-101 - +
MG132 + +
Ub
PD-1
USP24
PD-1
GAPDH
IP: PD-1
Input

Fig. 5B

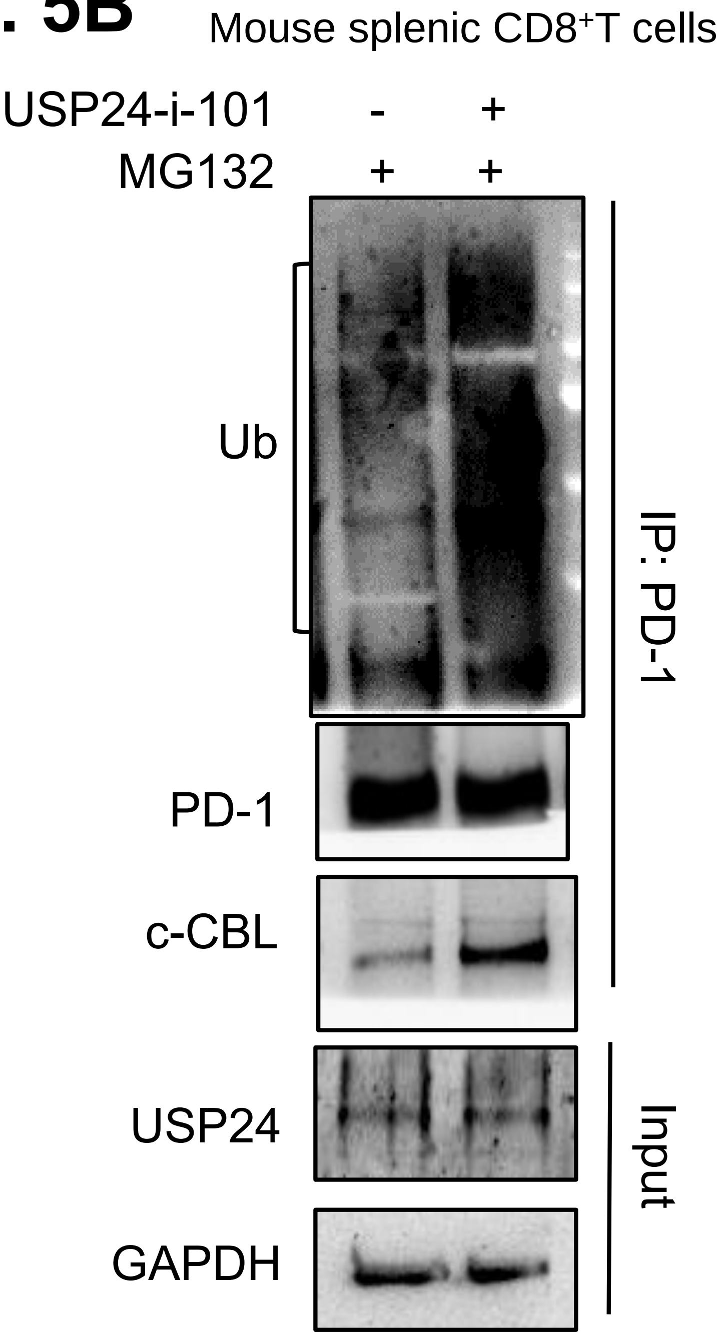

Fig. 5D

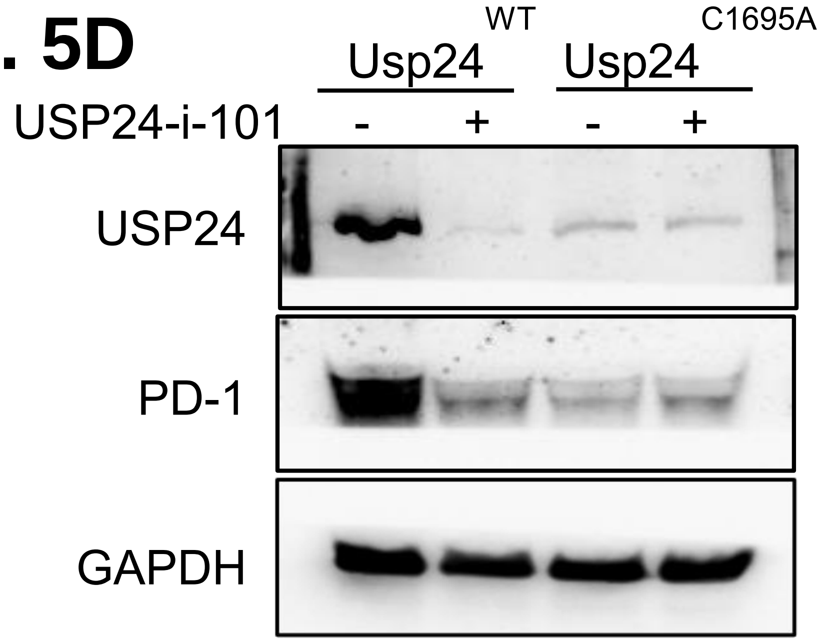

Fig. 5G

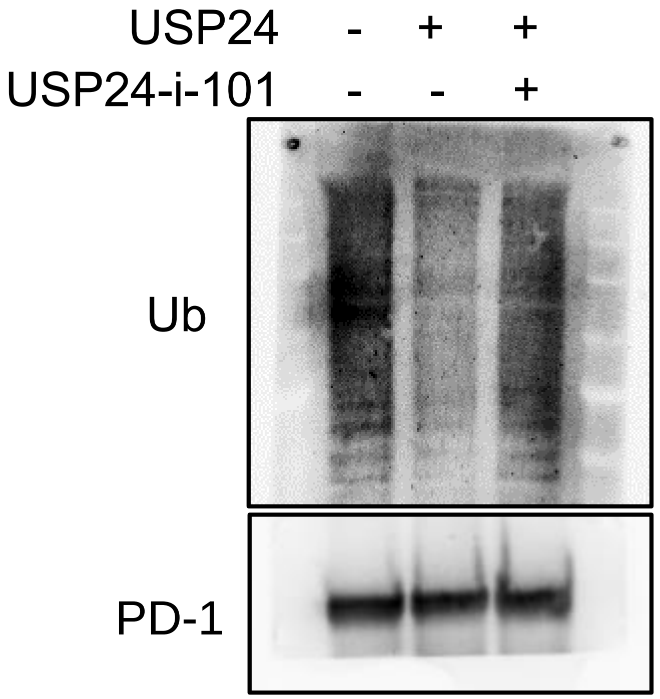

fig S2C

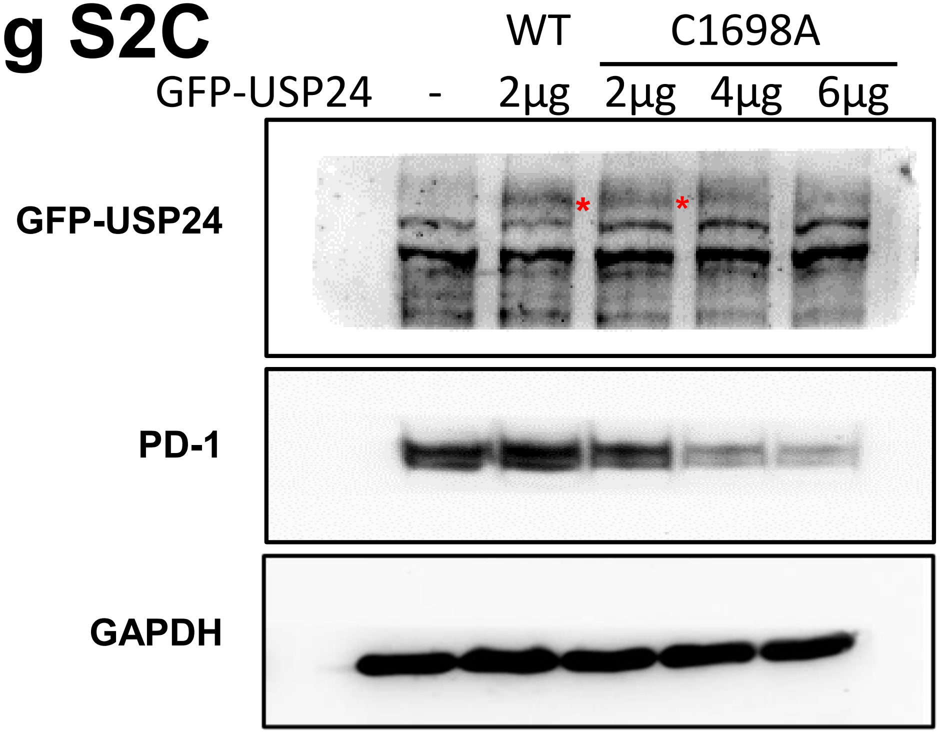

fig S2D

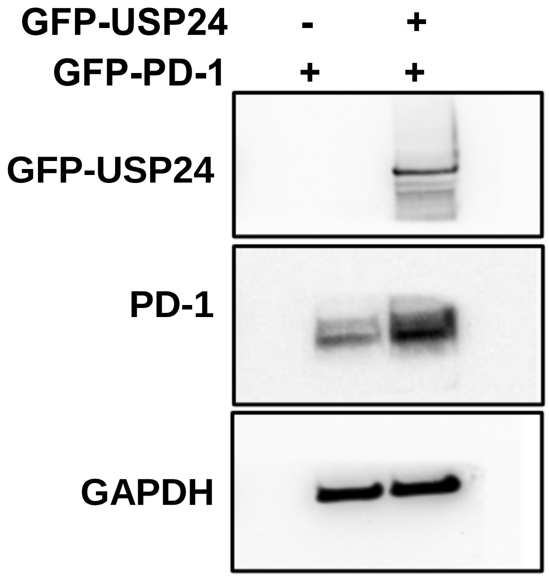

fig S2E

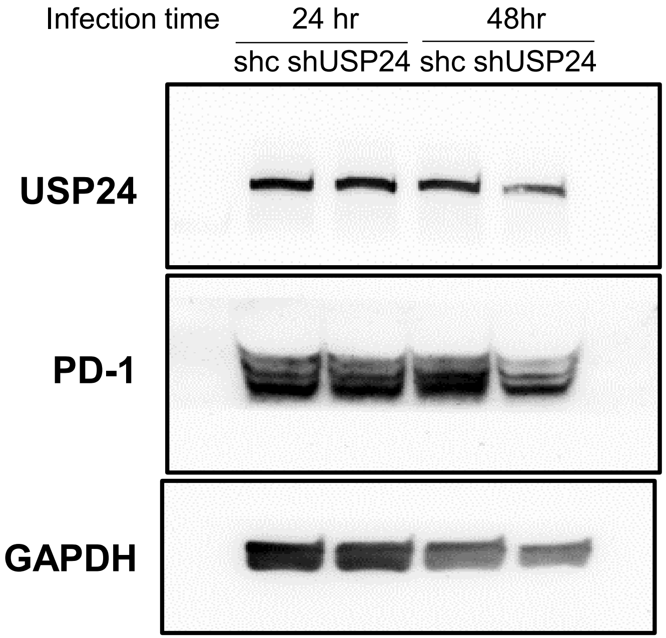

fig S2F

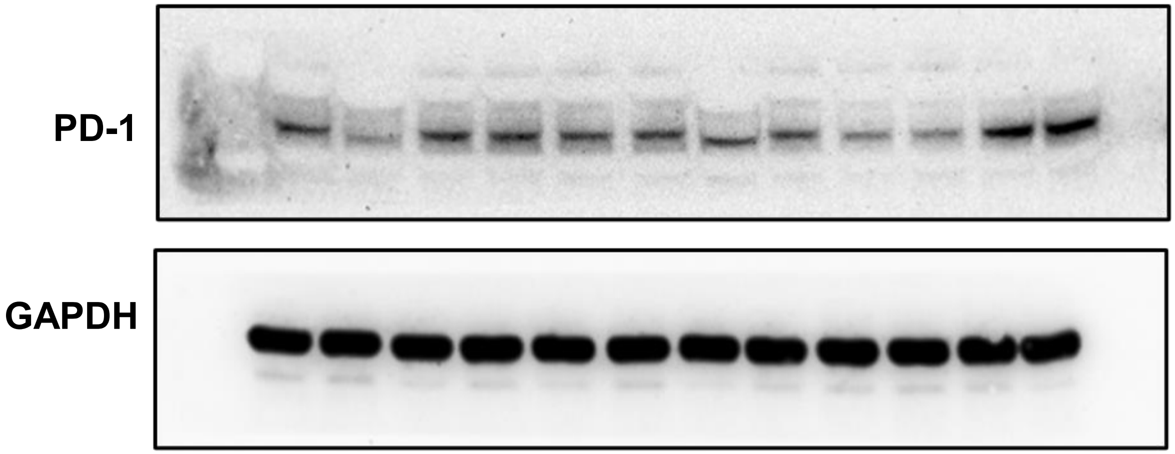

fig S2E

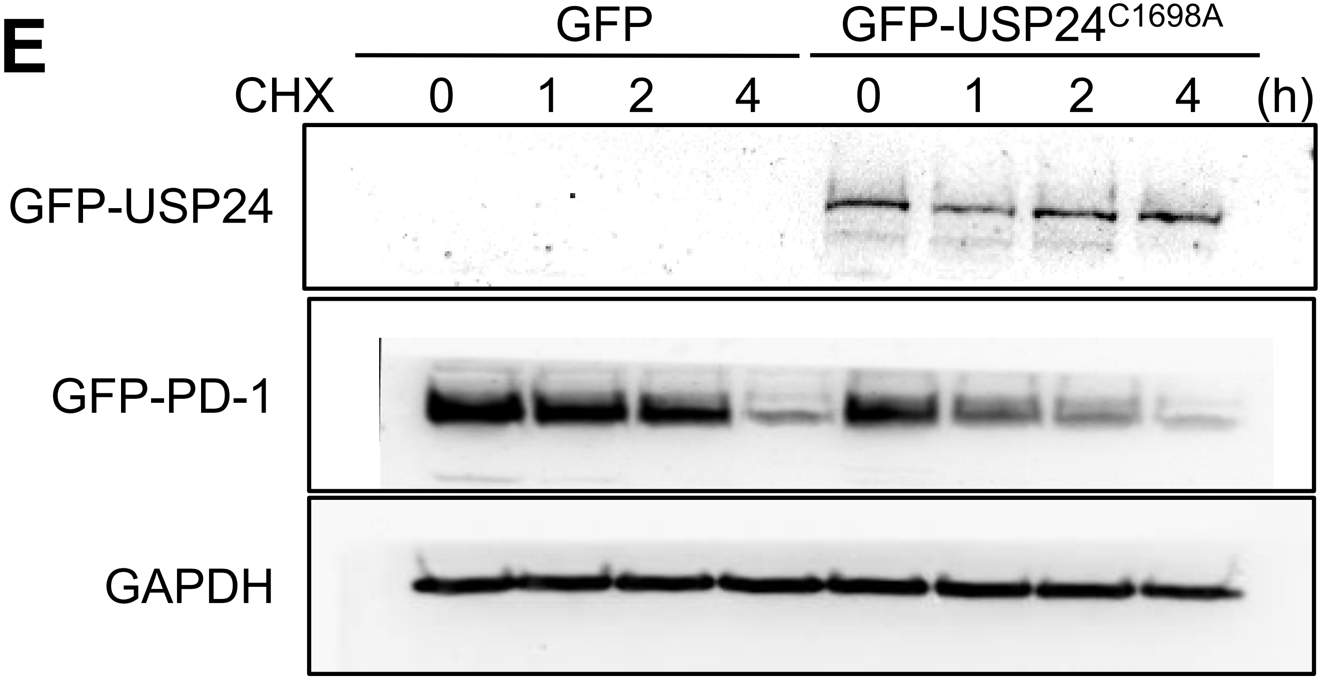

fig. S3A

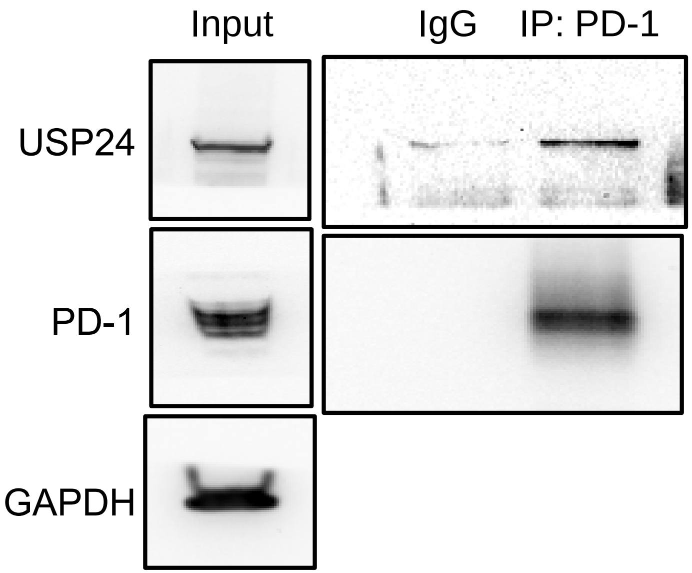

fig. S3B

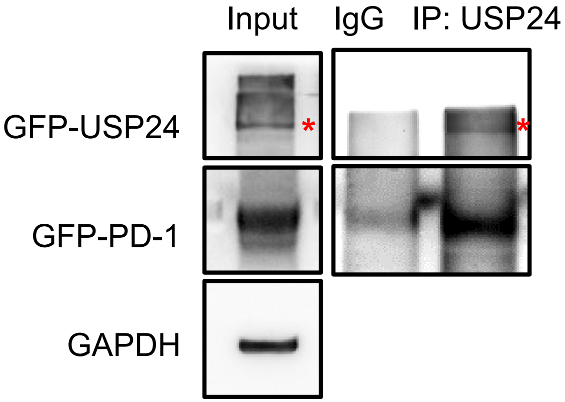

fig. S3C

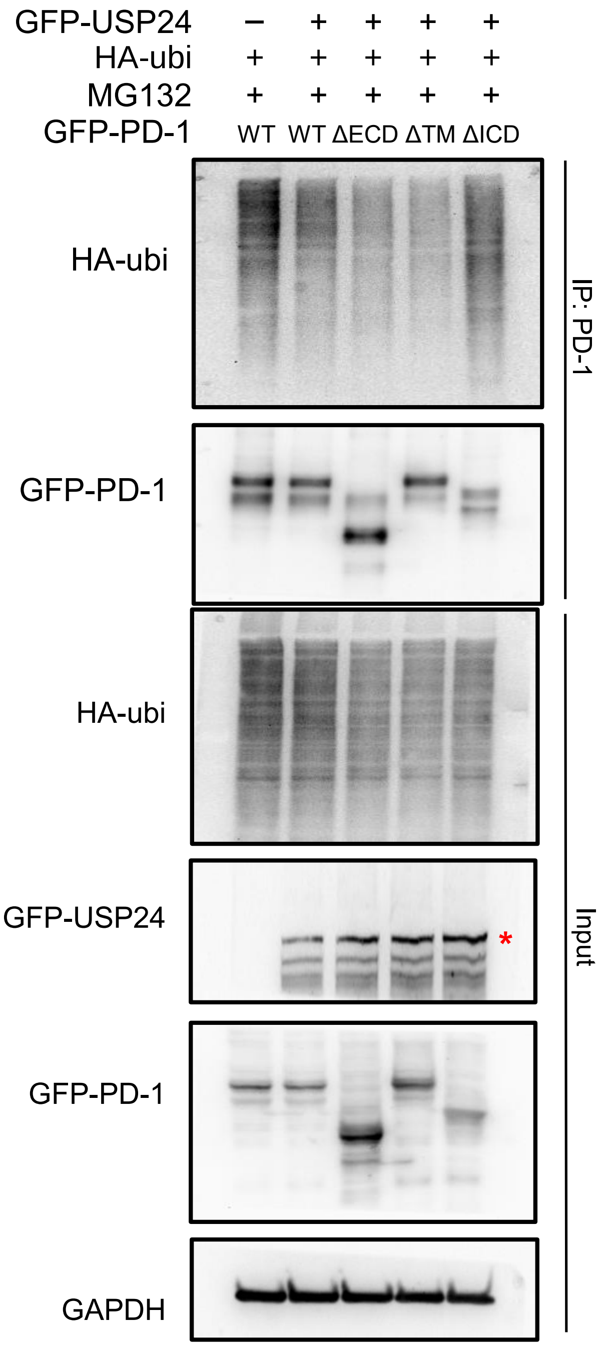

fig. S3D

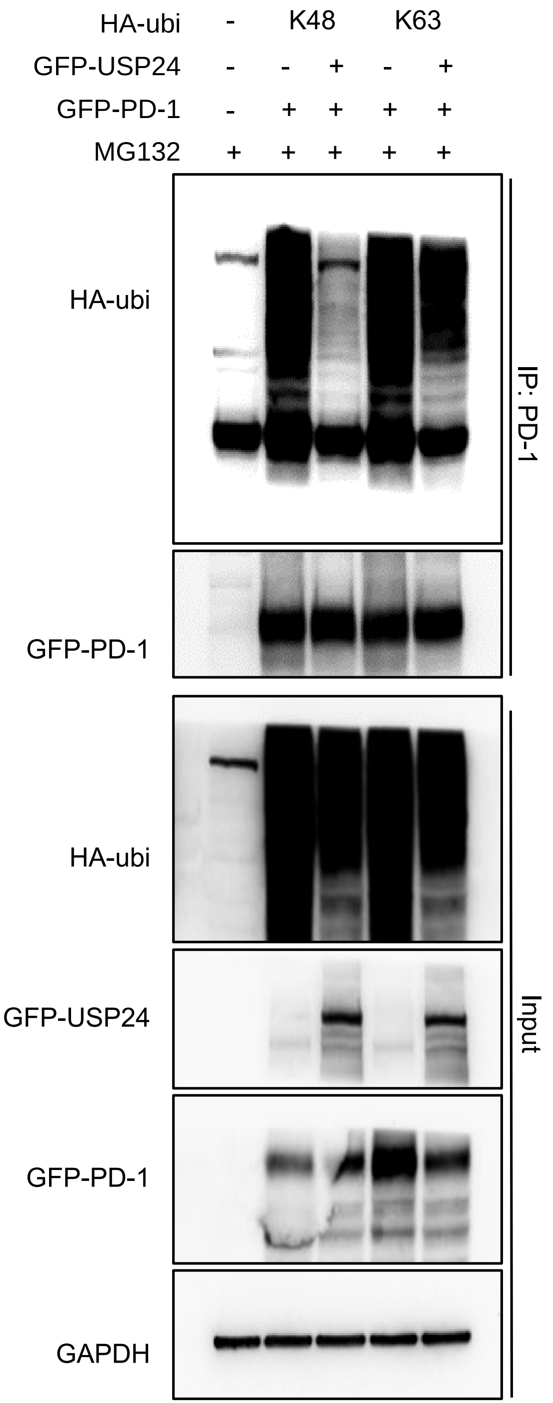

fig. S3E

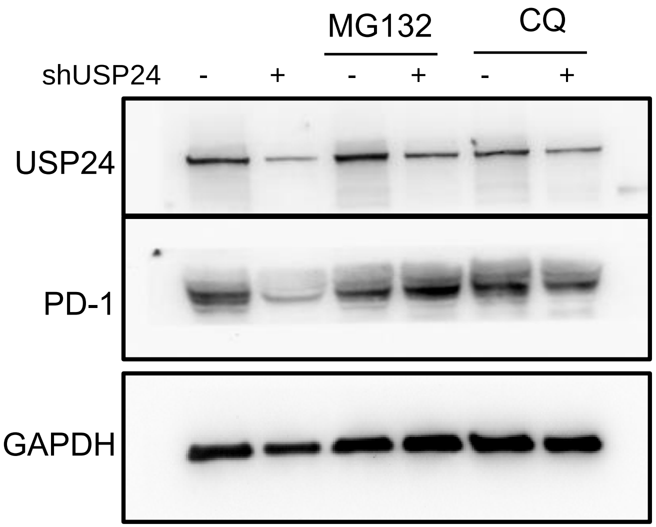

fig. S3F

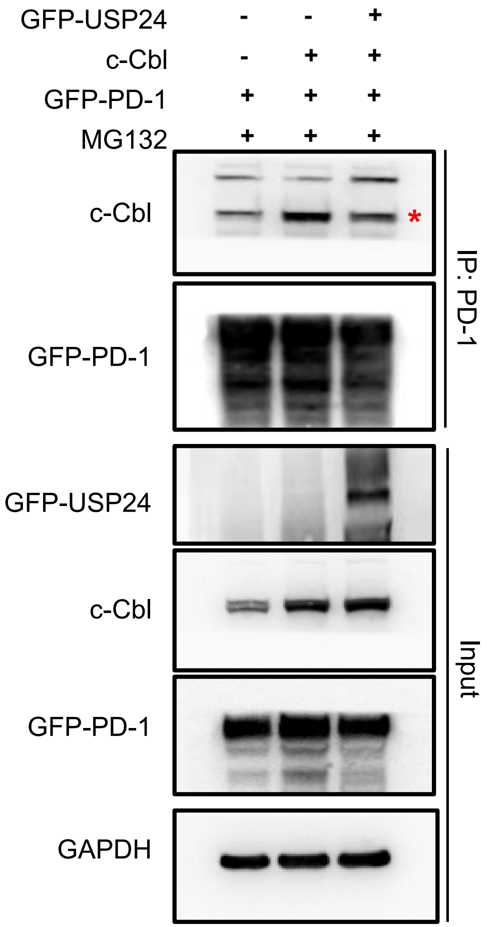

fig. S3G

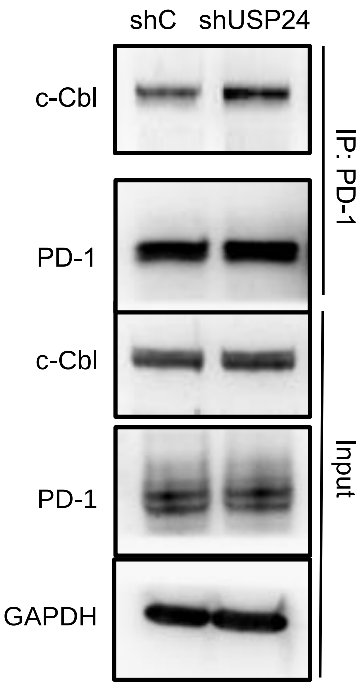

fig. S4A

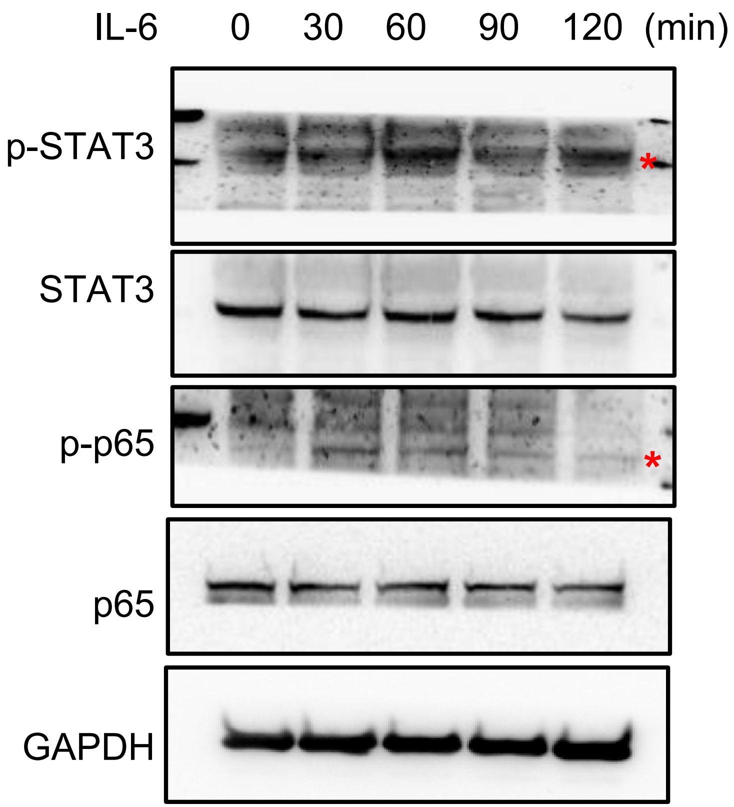

fig. S4D

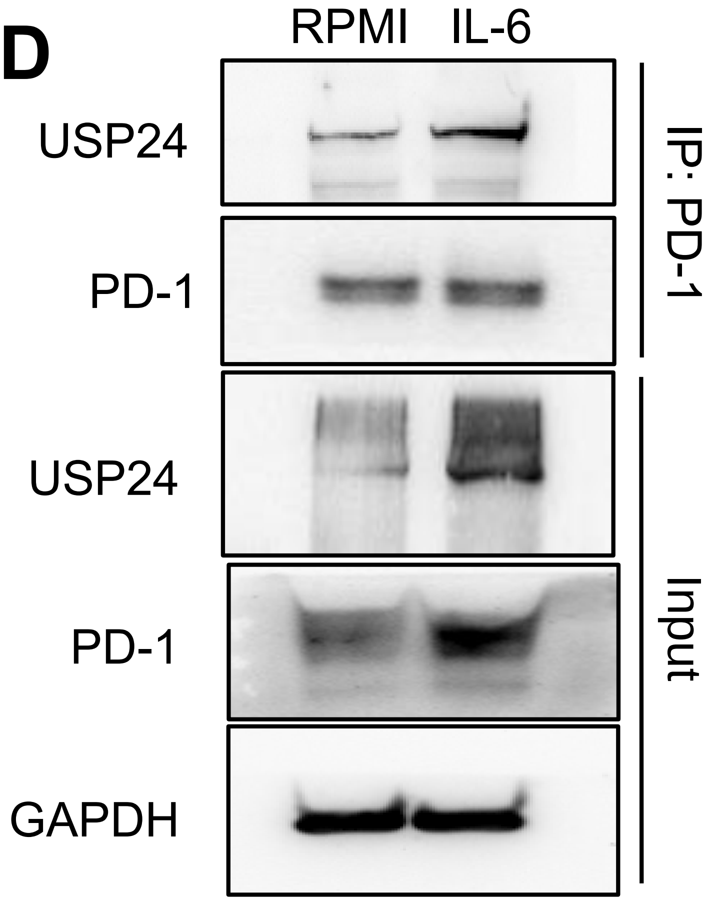

fig. S4E

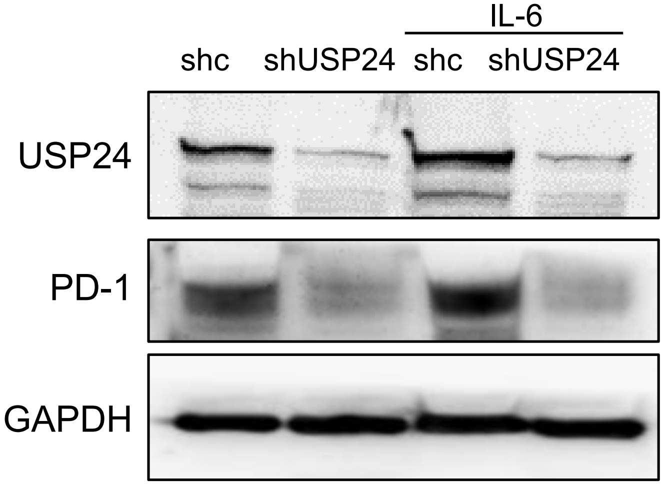

fig. S5B

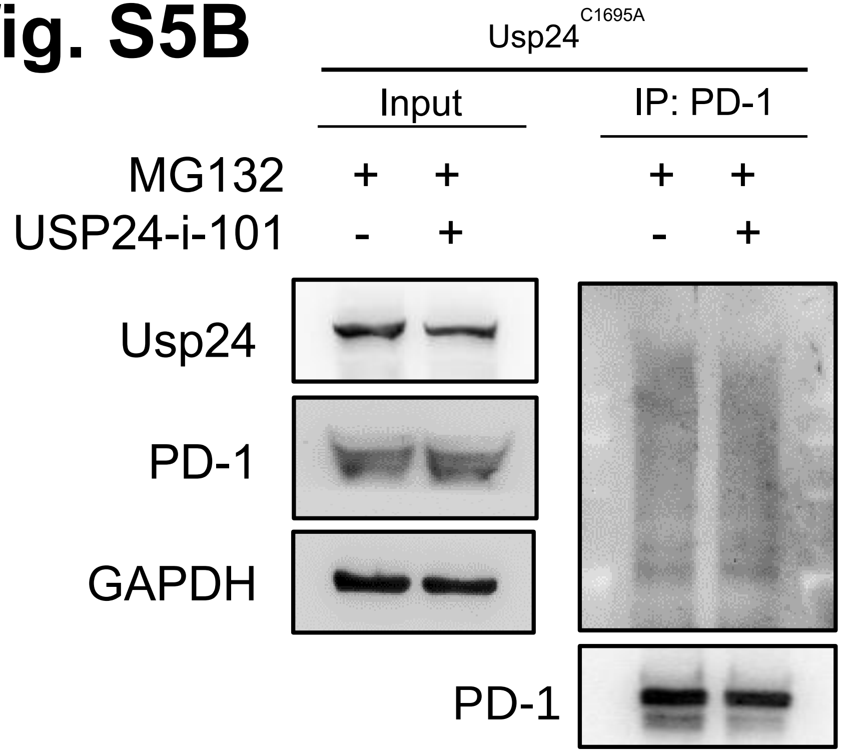

fig. S5C

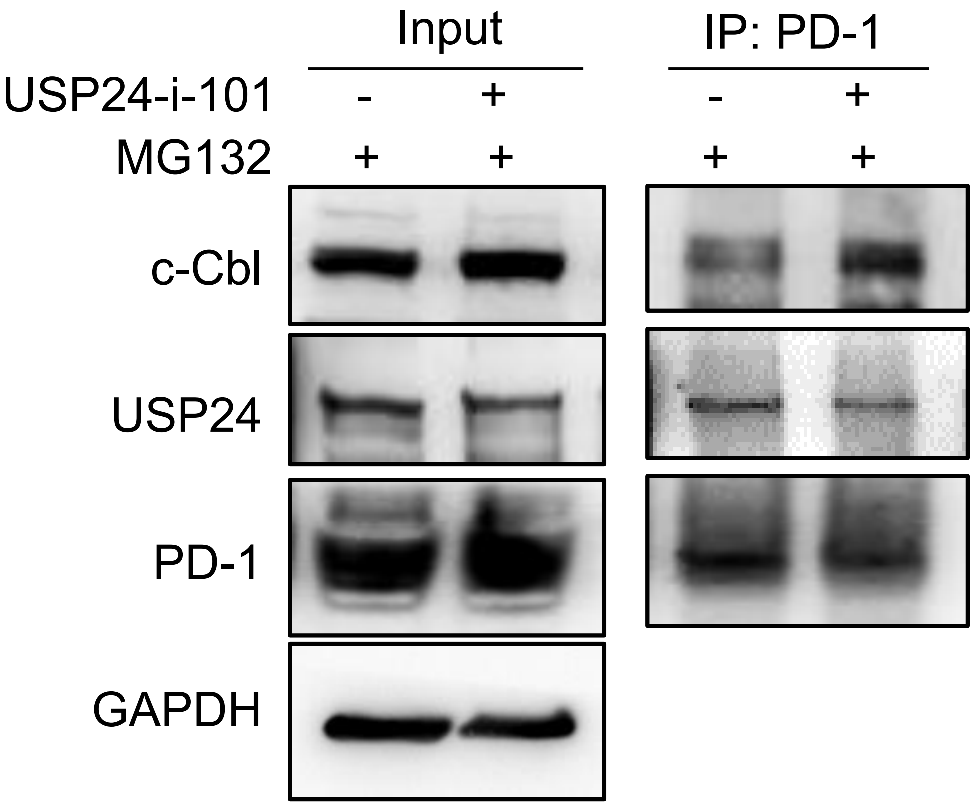

fig. S5I

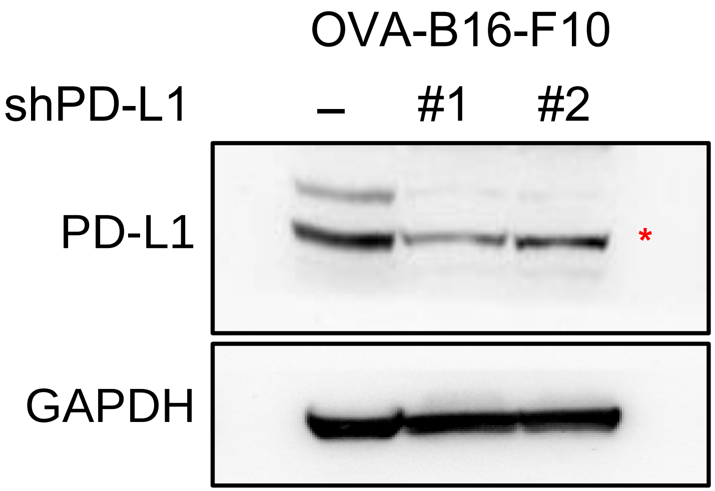

fig. S5O

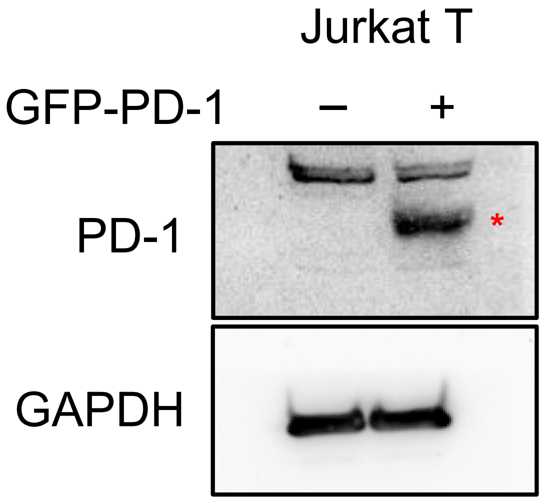

Supplement: Supplementary file 1 — Figs. S1 to S8 Tables S1 to S7 Data S1 [file sciadv.adt4258_sm.pdf]
